# Amino Acid-Coupled Bromophenols and a Sulfated Dimethylsulfonium Lanosol from the Red Alga *Vertebrata lanosa*

**DOI:** 10.3390/md20070420

**Published:** 2022-06-27

**Authors:** Joshua Jacobtorweihen, Marthe Schmitt, Verena Spiegler

**Affiliations:** 1 Institute for Pharmaceutical Biology and Phytochemistry, University of Münster, Corrensstraße 48, 48149 Münster, Germany; j.jacobtorweihen@uni-muenster.de (J.J.); marthe.schmitt@sigma-clermont.fr (M.S.); 2 SIGMA Clermont, 20 Avenue Blaise Pascal, TSA 62006, CEDEX, 63178 Aubière, France

**Keywords:** *Vertebrata lanosa*, bromophenol, amino acid derivatives, dimethyl sulfonium, sulfate

## Abstract

*Vertebrata lanosa* is a red alga that can commonly be found along the shores of Europe and North America. Its composition of bromophenols has been studied intensely. The aim of the current study was therefore to further investigate the phytochemistry of this alga, focusing more on the polar components. In total, 23 substances were isolated, including lanosol-4,7-disulfate (**4**) and the new compounds 3,5-dibromotyrosine (**12**), 3-bromo-5-sulfodihydroxyphenylalanine (**13**), 3-bromo-6-lanosyl dihydroxyphenylalanine (**14**), 3-(6′-lanosyl lanosyl) tyrosine (**15**) and 5-sulfovertebratol (**16**). In addition, 4-sulfo-7-dimethylsulfonium lanosol (**7**) was identified. While, in general, the dimethylsulfonium moiety is widespread in algae, its appearance in bromophenol is unique. Moreover, the major glycerogalactolipids, including the new ((5*Z*,8*Z*,11*Z*,14*Z*,17*Z*)-eicosapentaenoic acid 3′-[(6′’-*O*-α-galactopyranosyl-β-D-galactopyranosyl)]-1-glycerol ester (**23**), and mycosporine-like amino acids, porphyra-334 (**17**), aplysiapalythine A (**18**) and palythine (**19**), were identified.

## 1. Introduction

*Vertebrata lanosa* (L.) T.A. Christensen, formerly known as *Polysiphonia lanosa* (L.) Tandy, is a red alga belonging to the family of Rhodomelaceae. It is distributed commonly around the Atlantic shores of Europe and North America and the Baltic Sea [1], where it grows as an epiphyte on *Ascophyllum nodosum* [2]. Due to its characteristic aroma, it can be used as a spice [2], but also, the application of extracts as cosmetic ingredients is increasingly popular [3]. According to its widespread occurrence and usage, several studies have been conducted to investigate the phytochemical composition of *V. lanosa*, most of which focused on bromophenols. A series of different hydroxylated bromophenol derivatives, particularly lanosol (2,3-dibromo-4,5-dihydroxybenzyl alcohol) and similar benzyl alcohols or benzaldehydes, has been isolated [4,5,6,7]. These also included dimeric [6,7] or even tetrameric structures [7], as well as bromophenols with less common scaffolds, such as rhodomelol and methylrhodomelol [6,8], or vertebratol, which is composed of a dibromodihydroxybenzyl moiety linked to ornithin via an ureido group [6].

Regarding their bioactivities, selected lanosol derivatives [5,6] and dimeric bromophenols [6] were assayed for their antimicrobial properties against different Gram-positive and Gram-negative bacteria, but the inhibition of bacterial growth was either rather weak or absent [5,6]. Cytotoxic effects have been observed for certain bromophenolic compounds enriched in the dichloromethane fraction of a methanolic extract [9] but not for all bromophenols from *V. lanosa* in general [7].

Despite the numerous phytochemical investigations, much less is known about the more polar secondary metabolites of the alga. For example, only one sulfated bromophenol, namely 2,3-dibromo-5-hydroxybenzyl-1′,4-disulfate, has been reported to be present in a hydroethanolic extract from *V. lanosa* [10] and was isolated as dipotassium salt [11,12]. Further, the galactan sulfate of the red alga has been characterized as a polysaccharide of the agar class, containing methylated and sulfated units of d- and l-galactose [13]. The high quantities of the polysaccharide [13] could possibly contribute to the use of *V. lanosa* extracts in cosmetics as a moisturizing component [3,14]. Moreover, the presence of mycosporine-like amino acids (MAA) [15], which are known for their very efficient absorbance of UV radiation [16,17], might have an additional value in the seaweed extract for cosmetical purposes. Similar to the bromophenols [18], seasonal variations of MAA patterns in red algae [15] have to be considered. The composition of MAA in *V. lanosa* has been investigated using LC-ESI-qTOF-MS, resulting in the identification of several known compounds, such as porphyra-334 or palythine. In addition, the presence of MAAs previously undescribed for this species was also indicated [15], however, no MAAs have been isolated from *V. lanosa* for structure elucidation until now.

The aim of the current study was, therefore, to further characterize the phytochemical composition of *V. lanosa*, with a focus on the more polar compounds in particular, including the major MAA of the alga. Partitioning of a methanol–water extract between water and ethyl acetate led to an enrichment of sulfated bromophenols and MAAs, with a high amount of polysaccharides. Thirteen compounds were isolated from the aqueous phase, including three new sulfated bromophenols and one bromophenol carrying a unique sulfonium moiety. Their structures were elucidated by means of mass spectrometry and NMR spectroscopy. Furthermore, one new galactolipid was obtained from the ethyl acetate partition, together with eleven known bromophenols and galactolipids.

## 2. Results

### 2.1. Sulfated Bromophenols and Amino Acid Derivatives

**Figure 1:**
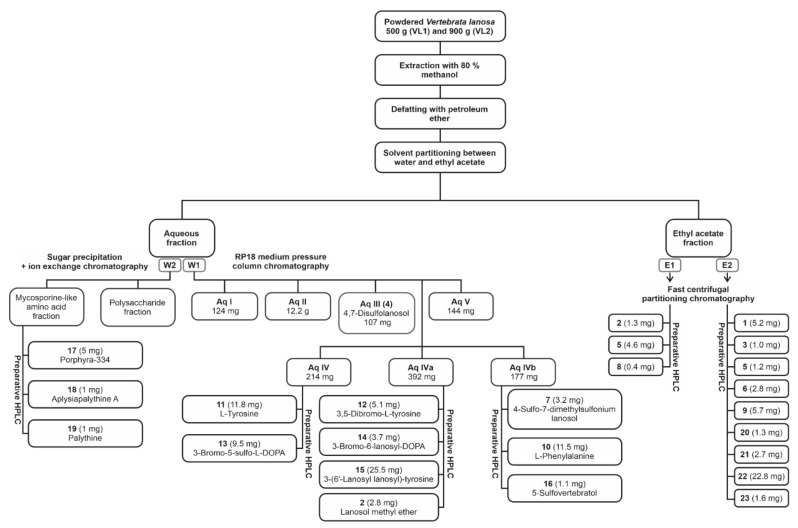
Extraction and fractionation procedures of *Vertebrata lanosa*.

In order to characterize the more polar components in a methanol–water (8:2 *v*/*v*) extract from *V. lanosa*, the crude extract was partitioned between ethyl acetate and water. The aqueous phase W1 was then fractionated by RP18 medium pressure column chromatography, which led to the isolation of lanosol-4,7-disulfate **4** (107 mg, Aq III) together with subfractions Aq I–Aq V. Resorcinol sulfuric acid tests and UPLC-MS analysis revealed the presence of polysaccharides and mycosporine-like amino acids (MAAs) in fractions Aq I and II and a variety of bromophenols in fractions Aq IV and Aq V. Hence, subfractions Aq IV, Aq IVa and Aq IVb were further fractionated by preparative HPLC, affording lanosol methyl ether (**2,** 2.8 mg), 4-sulfo-7-dimethylsulfonium lanosol (**7,** 3.2 mg), L-phenylalanine (**10,** 11.5 mg), L-tyrosine (**11,** 11.8 mg), 3,5-dibromotyrosine (**12,** 5.1 mg), 3-bromo-5-sulfo-L-dihydroxyphenylalanine (**13,** 9.5 mg), 3-bromo-6-lanosyl dihydroxyphenylalanine (**14,** 3.7 mg), 3-(6′-lanosyl lanosyl)-tyrosine (**15,** 25.5 mg) and 5-sulfovertebratol (**16,** 1.1 mg), of which **7** and **12**–**16** are new natural products. The known bromophenols **2** and **4** were identified by comparison of the NMR and MS data with the literature data [6,19]. Figure 1 shows the fractionation procedure.

**Figure 2:**
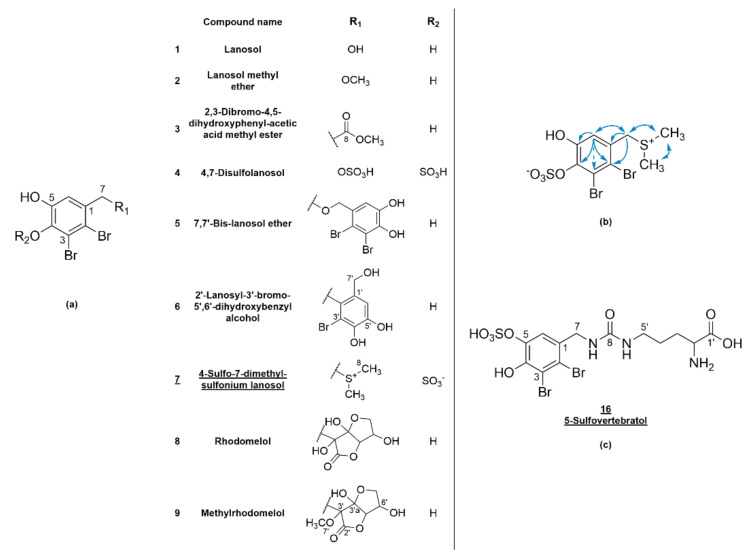
(**a**) Structures of isolated lanosol derivatives **1**–**9** from *V. lanosa*. (**b**) Key HMBC correlations of 4-sulfo-7-dimethylsulfonium lanosol (**7**). (**c**) Chemical structure of 5-sulfovertebratol (**16**). New compounds are underlined.

The fragmentation of **7** in positive- and negative-mode HR-ESI-MS indicated the presence of a sulfate moiety (*m/z* 345/343/341 [M – SO_3_]^+^), a lanosol scaffold (*m/z* 283/281/279 [C_7_H_5_Br_2_O_2_]^+^) and, by the cleavage of two methyl groups (*m/z* 409/407/405 [M – CH_3_]^−^ and *m/z* 393/391/389 [M – C_2_H_6_]^−^), also a heteroatom in the side chain carrying the methyl groups (*m/z* 361/359/357 [M – C_2_H_6_S]^−^). This hypothesis was further corroborated by the calculated sum formula C_9_H_11_^79^Br_2_O_5_S_2_^+^ of the [M + H]^+^ adduct (*m/z* 420.8463), suggesting a sulfonium moiety in the side chain. The presence of two bromine atoms could also be deduced from the isotopic pattern in the intensity ratio of 1:2:1. An analysis of the ^1^H-, ^13^C- and 2D-NMR spectra (Table 1 and Figure 2b) also supported a sulfonium substructure by the shifts of the benzylic carbon (δ_C_ 50.16) and the methyl groups (δ_C_ 25.28, δ_H_ 2.97); as in the literature, similar shifts have been reported for sulfonium moieties [20]. HMBC correlations from the benzylic position to methyl groups C-8, C-9 and the aromatic carbons C-2 and C-6 confirmed the connection between the two subunits. The position of the sulfate moiety was assigned due to a stronger signal intensity of the aromatic proton to C-4 (δ_C_ 141.40) in the HMBC spectrum in comparison to C-5 (δ_C_ 151.20) and the shift similarity of the aromatic signals in the known metabolite **4** (Supplementary Materials) [19]. The structure of **7** was therefore established as 4-sulfo-7-dimethylsulfonium lanosol.

An analysis of the ^1^H-, ^13^C- and 2D-NMR data of **12**–**15** showed that all of them possess carboxylic carbons (δ_C_ approx. 173) adjacent to a α-carbon with an amino function (δ_C_ approx. 55) and a benzylic β-carbon (δ_C_ approx. 35) (Table 2), indicating a phenylalanine or tyrosine scaffold. For **12**, the (+)ESI-MS data also supported the amino acid substructure by the occurrence of [M – NH_3_]^+^ (*m/z* 325/323/321) and [M – HCOOH]^+^ (*m/z* 296/294/292) fragments. The calculated sum formula for the [M + H]^+^ adduct of **12** (*m/z* 337.8983, C_9_H_10_^79^Br_2_NO_3_^+^) suggested **12** to be a dibromotyrosine derivative, which was corroborated by the ^1^H-NMR data being consistent with the literature values for synthesized 3,5-dibromo-L-tyrosine [21]. The configuration of α-carbon was assigned by the optical rotation ([α]^20^_D_ = −67.7) in comparison to the literature data of tyrosine [22]. This is the first report of this compound being isolated from its natural source.

For **13,** the occurrence of a fragment resulting from the loss of a sulfate group (*m/z* 276/274 [M – SO_3_]^−^) could be observed in (-)ESI-MS. The isotopes of the [M – H]^−^ ion cluster (*m/z* 355.9304/353.9326) showed relative intensities of 1:1 and gave a calculated sum formula of C_9_H_9_^79^BrNO_7_S^−^ for the monoisotopic mass. HMBC correlations of the aromatic and the benzylic protons were used to elucidate the substitution of the phenyl ring: the β-protons showed intense HMBC correlations with the unsubstituted aromatic carbons (δ_C_ 132.12 (C-2) and 123.92 (C-6)). H-2, in turn, strongly correlated to β-carbon; C-3; C-4 and C-6 (δC 35.37, 111.84, 146.47 and 123.92, resp.) and H-6 to β-carbon; C-2; C-4 and C-5 (δ_C_ 35.37, 132.12, 146.47 and 140.47, resp.). Additionally, both protons showed weak four-bond correlations to the respective carbons. The sulfate moiety was determined to be attached to C-5 by the relative upfield shift of the respective carbon in comparison to unsubstituted phenolic carbons. The configuration of **13** was also determined by optical rotation ([α]^20^_D_ = −10.4) and comparison to the literature data of tyrosine [22]. Hence, the chemical structure of **13** was found to be 3-bromo-5-sulfo-L-dihydroxyphenylalanine.

Both **14** and **15** showed substituted aromatic carbons with similar shifts (C-6, δ_C_ 128.62 and C-3, δ_C_ 126.47, resp.) in their ^13^C-NMR spectra. For both of these carbons, compared to unsubstituted tyrosine, the expected upfield shift in the *ortho*-position to the hydroxyl group was not apparent, suggesting aliphatic or benzylic substituents attached to the tyrosine scaffold. Further analysis of the HMBC correlations revealed the presence of either one (**14**) or two (**15**) lanosyl moieties attached to C-6 (**14**) or C-3 (**15**). The isotopic pattern of the [M – H]^−^ ion of **14** (*m/z* 557.8233/555.8243/553.8265/551.8285) in the relative abundance of 1:3:3:1 indicated the presence of three bromine atoms in the molecule, suggesting a bromine substitution in the dihydroxyphenylalanine scaffold in addition to the lanosyl moiety. The position of the third bromine-substituted carbon was deduced from the strong upfield shift and the HMBC correlations from H-2 to C-3 (δ_C_ 109.97) to be at position 3. Thus, the chemical structure of **14** was determined to be 3-bromo-6-lanosyl dihydroxyphenylalanine.

**Figure 3:**
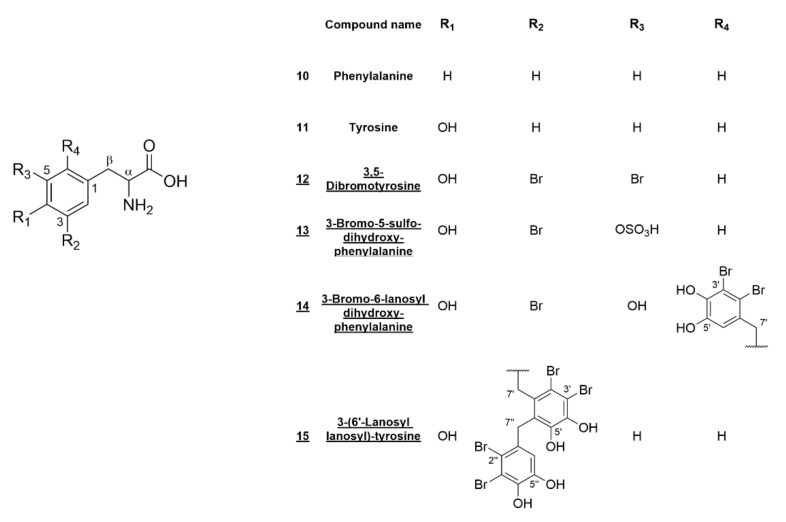
Structures of the isolated aromatic amino acid derivatives **10**–**15** from *V. lanosa*. New compounds are underlined.

The second lanosol residue of **15** is connected to C-6′ via its benzylic carbon. For **15,** four bromine atoms were confirmed by the relative isotopic abundance of the [M – H]^−^ ion cluster (*m/z* 743.7806/741.7758/739.7778/737.7803/735.7761) in the ratio of 1:4:6:4:1. The assignment of certain carbons (δ_C_ 113.94, C-3′ and δ_C_ 143.33, C-4′) was not possible from HMBC correlations, since there are no protons in the molecular vicinity. Based on the substitution of the 2′’,3′’-dibromo-4′’,5′’-dihydroxybenzyl moiety and since *p*-hydroxybenzaldehyde is the biogenetic origin of the lanosol scaffold [23], we propose the reported assignments (see Table 2) and, therefore, the structure of **15** to be 3-(6′-lanosyl lanosyl)-tyrosine. The structures of compounds **10**–**15** are given in Figure 3.

The HR-ESI-MS of **16** showed an [M + H]^+^ adduct at *m/z* 533.9257, inferring the sum formula C_13_H_18_^79^Br_2_N_3_O_8_S^+^. The presence of sulfur was deduced from a fragment at *m/z* 453.9645 [M – SO_3_]^+^ (C_13_H_18_^79^Br_2_N_3_O_5_), which corresponds to vertebratol [6]. NMR data were mostly consistent with those of vertebratol [6], except for benzylic carbon (δ_C_ 75.85), which was shifted about 30 ppm downfield, and the brominated aromatic carbons (δ_C_ 114.34 and 112.70, see also Table 1). Once again, the position of the sulfate moiety was assigned by HMBC correlations of H-6 to C-4 and C-5 and, additionally, by comparison of the simulated spectra of the respective 4- and 5-sulfo derivatives. Thus, the structure of **16** was found to be 5-sulfovertebratol (Figure 2c). Due to the low yield, the configuration of **16** could not be determined.

### 2.2. Mycosporine-Like Amino Acids

**Figure 4:**
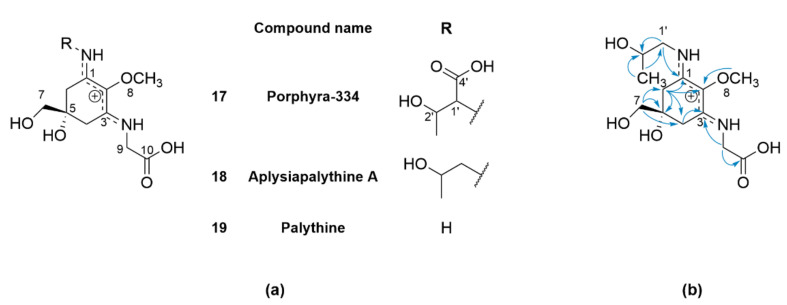
(**a**) Structures of the isolated mycosporine-like amino acids **17**–**19** from *V. lanosa*. (**b**) Key HMBC correlations of aplysiapalythine A (**18**).

The presence of several MAAs in *V. lanosa* has been reported recently, however, not all MAAs could be unambiguously identified by LC-MS [15]. Therefore, the aqueous-phase W2 was further fractionated to obtain pure MAAs. Briefly, W2 was first depleted of polysaccharides by ethanolic precipitation and, subsequently, fractionated via cation exchange chromatography on Amberlite™ IR120 (H) resin. The fraction containing the highest amount of MAA (Amb9) was finally purified by preparative HPLC to obtain **17** (5 mg), **18** (1 mg) and **19** (1 mg). The structures of these MAAs are given in Figure 4a. To assess the purity of the compounds and effectiveness of the fractionation procedure, a HPLC method using a HILIC stationary phase was developed to facilitate the targeted isolation of the desired MAA. The isolated compounds correspond to porphyra-334 (**17**), palythine (**18**) and aplysiapalythine A (**19**), respectively, as determined by the comparison of HR-ESI-MS and NMR data (see Supplementary Materials) with the literature [24,25]. Compound **19** was distinguished from its isomer palythinol by the HMBC correlation of methylene protons H-1′ (δ_H_ 3.48 and 3.45) to C-1 (δ_C_ 162.72) in the cyclohexenimine ring [24].

### 2.3. Bromophenols and Glycerogalactolipids

**Figure 5:**
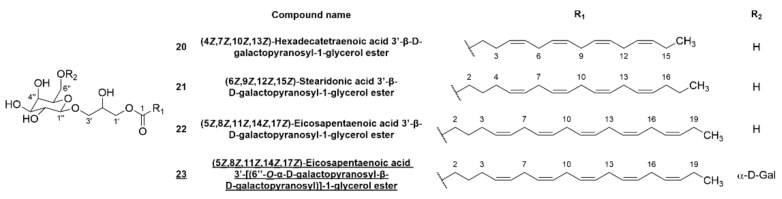
Structures of isolated fatty acid glycerogalactosides **20**–**23** from *V. lanosa*. New compounds are underlined.

In addition to the characterization of the aqueous phase of the methanolic extract, the composition of the ethyl acetate phase was investigated by initial fractionation via FCPC and subsequent preparative HPLC of the fractions obtained (Figure 1). For E1, this method yielded bromophenols lanosol methyl ether (**2**, 1.3 mg), **5** (7,7′-bis-lanosol ether, 4.6 mg) and **8** (rhodomelol, 0.4 mg). The application of the same method to E2 facilitated the isolation of **1** (lanosol, 5.2 mg), **3** (2,3-dibromo-4,5-dihydroxyphenyl acetic acid methyl ester, 1.0 mg), **5** (7,7′-bis-lanosol ether, 1.2 mg), **6** (2′-lanosyl-3′-bromo-5′,6′-dihydroxybenzyl alcohol, 2.8 mg), **20** ((4*Z*,7*Z*,10*Z*,13*Z*)-hexadecatetraenoic acid 3′-β-D-galactopyranosyl-1-glycerol ester, 0.6 mg), **21** ((6*Z*,9*Z*,12*Z*,15*Z*)-stearidonic acid 3′-β-D-galactopyranosyl-1-glycerol ester, 2.7 mg) and **22** ((5*Z*,8*Z*,11*Z*,14*Z*,17*Z*)-eicosapentaenoic acid 3′-β-D-galactopyranosyl-1-glycerol ester, 22.8 mg). Figure 5 shows the structures of the isolated glycerogalactolipids. The extruded stationary phase (G II) of E2 was subjected to further fractionation by FCPC with a different solvent system, which ultimately led to the isolation of **9** (methylrhodomelol, 5.7 mg), **20** (0.7 mg) and **23** ((5*Z*,8*Z*,11*Z*,14*Z*,17*Z*)-eicosapentaenoic acid 3′-[(6′’-*O*-α-galactopyranosyl-β-D-galactopyranosyl)]-1-glycerol ester, 1.6 mg) via preparative HPLC.

Compounds **1**, **2**, **3**, **5**, **6**, **8** and **9** (Figure 2) represent the known bromophenols and were identified by comparison of the NMR and MS data to the literature data [6,26,27]. The structures of **20**–**22** were verified by comparison of the literature data [28,29,30] to the measured MS and NMR spectra (Supplementary Data), followed by sugar hydrolysis and the identification by thin layer chromatography (TLC) and capillary zone electrophoresis (CZE). The NMR data for **23** were very similar to those of **22**, suggesting the presence of an eicosapentaenoic acid moiety connected to a 3′-β-glucopyranosylglycerol moiety via a C-1′ ester. Moreover, six additional signals were present in the ^1^H- and ^13^C-NMR spectra, indicating a further galactosyl moiety. By the HMBC correlation of H-6″ (δ_H_ 3.70/3.56) to C-1′″ (δ_C_ 101.10) and the small coupling constant of H-1′″ (δ_H_ 4.84, d, *J*_1,2_ 3.6 Hz), the α-1,6 glycosidic linkage was determined [31]. The identity of the digalactoside was confirmed by sugar hydrolysis and comparison to the reference compounds on TLC. The D-configuration was subsequently determined by CZE analysis after diastereomeric derivatization. This led to the identification of **23** as (5*Z*,8*Z*,11*Z*,14*Z*,17*Z*)-eicosapentaenoic acid 3′-(6″-*O*-α-D-galactopyranosyl-β-D-galactopyranosyl)-1-glycerol ester (Figure 5).

## 3. Discussion

Rhodomelaceae, especially the genus *Vertebrata* (syn. *Polysiphonia*), are known to frequently contain haloaryl derivatives [32], and a series of bromophenolic compounds was isolated from a hydroalcoholic extract of *V. lanosa*. Lanosol (**1**), representing the simplest structure, has been isolated from various red algae previously and is reported to possess cytotoxic [9], antiviral [33], antioxidant [34] and glucosidase-inhibiting [35] activity in vitro [32,36]. Similarly, the methyl ether of lanosol (**2**) showed cytotoxic [9] and antimicrobial [27] effects [32,36], although it might be an isolation artifact formed during methanolic extraction [5]. This might also be the case for **3**, which has not been isolated from algae before, unlike the native phenyl acetic acid derivative found in *Rhodomela confervoides* [37]. Compared to simple lanosols, dimeric bromophenols, such as **5**, seem to enhance the observed in vitro effects [6,7,38,39,40,41] and, additionally, showed antifungal [42] and anti-inflammatory [43] activity. Further, **5** and, to a lesser extent, also **6** have been reported to inhibit glucose-6-phosphate dehydrogenase [40,44]. It should, however, be mentioned that the observed biological activities of bromophenols were often relatively weak—in particular, the antimicrobial effects [6,32,36]—and, with the exception of some compounds such as **8**, rather unselective [36]. Kurihara et al. pointed out that enzyme inhibition could be the result of an *o*-quinone addition to the respective proteins [35], and as Baell emphasized, compounds with a variety of reported moderate bioactivities should be considered as potential drug leads only with caution, as, particularly, molecules with catechol substructures are prone to interfering with multiple in vitro assays [45].

In addition to the more frequently occurring bromophenols, two new brominated tyrosine derivatives (**12** and **13**) and two amino acids coupled to lanosyl moieties (**14** and **15**) were also isolated. A bromotyrosine has previously been found in *Rhodomela confervoides* [46], as well as derivatives of lanosol coupled to pyroglutamate [47]. So far, L-tyrosine (**11**) has been considered the main precursor of bromophenols, with the respective hydroxy benzoic acid derivatives being brominated at later steps in biosynthesis [23]. On the other hand, as also suspected by Ma et al. [46], the presence of **12** and **13** suggests that bromination occurs at a much earlier stage of biosynthesis. Whether bromoperoxidases in *V. lanosa*, unlike other macroalgae [48], are capable of directly converting L-tyrosine remains a topic for further research.

Another intriguing finding with respect to the biosynthesis is that, for *Vertebrata* species, typically, 2,3-dibrominated benzyl moieties have been reported (*Vertebrata decipiens* (syn. *Polysiphonia decipiens*) [38] and *V. lanosa* [6]). This is well in line with most of the compounds isolated in this study (**1**–**7** and **14**–**16**); however, **12** is an exception from this substitution pattern. The 3,5-dibrominated benzyl moiety, as in **12**, is usually found in members of the *Polysiphonia* genus (*Polysiphonia stricta (syn. P. urceolata*) [49] and *P. morrowii* [50]), and the occurrence of **12** in *V. lanosa* might therefore be a hint for the presence of two regioselective bromoperoxidases in this alga.

Along with the amino acid-coupled compounds, sulfated bromophenols (**4**, **7**, **14** and **16**) were also isolated. Lanosol-4,7-disulfate (**4**) is one of the major secondary metabolites within the extract, and its occurrence in *V. lanosa* has been known for a long time [11,12]. Probably due to its high quantity, it has previously even been assumed to be the only sulfated compound in extracts from this alga [10]. On the other hand, the presence of sulfates could depend on parameters like the extraction procedure, as suggested by Weinstein et al., who suspected lanosol and its derivatives to be artifacts [51]. Despite this, Barreto and Meyer still found the lanosol disulfate ester in *Osmundaria serrata* after an extraction procedure of one week [52]. Taking into account that salt, unlike the unsulfated lanosols, was inactive as a feeding deterrent [53], they proposed bromophenols to be stored in algae as inactive salts and the release of lanosols upon injury [52]. On the other hand, Ma et al. did not find a significant difference in the cytotoxicity of a certain bromophenol versus its sulfate ester [46]. Within the current study, we also found the sulfate ester of vertebratol (**16**), a bromophenol that was just recently isolated from *V. lanosa* [6], but we cannot infer from our data which compound is genuine to the plant.

In contrast, compound **7** represents a unique structure containing a dimethyl sulfonium group. Generally, methyl sulfonium moieties, particularly dimethyl sulfonioproionate (DMSP), are widely distributed and also present in marine algae [54], including *V. lanosa* [55,56], where it acts as an antioxidant [57], cryoprotectant and osmolyte [54,58,59]. DMSP is biosynthesized in several steps via methionine [59] and degraded enzymatically to dimethylsulfide by DMSP lyases [56]. In the case of **7**, the C-3 alkyl side chain is formally replaced by a dibromo dihydroxy benzyl moiety. Whether the sulfur atom is derived from an amino acid, e.g., cysteine, similar to the biosynthesis of methionine [60], and at what stage of lanosol formation remains a subject for further research. Interestingly, a bromophenol containing a similar sulfoxide structure was previously isolated from *Rhodomela confervoides* [47]. It could be regarded as an oxidation product of **7** comparable to dimethyl sulfoxide (DMSO) produced from DMSP in bacteria and microalgae [61,62]. Accordingly, **7** could be a precursor of the respective sulfoxide reported by Zhao et al. [47], and it would be interesting to explore if there are further sulfoxonium intermediates that could suggest a route of oxidation similar to the biosynthesis of DMSO from DMSP via dimethyl sulfoxonium propionate (DMSOP) [62]. However, the conversion of DMSOP to DMSO has been experimentally confirmed in bacteria [62], and the enzymatic cleavage of DMSP in *V. lanosa* is reported to yield mainly dimethyl sulfide [56]. On the other hand, Lee and de Mora summarized their findings supporting the hypothesis of intracellular production of DMSO by algae [61]. If such a biosynthetic route is applicable to a structurally distinct substrate such as **7** remains, of course, highly speculative.

Apart from bromophenols, several glycerogalactolipids (**20**–**22** and the new compound **23**) were isolated. They represent a class of fatty acid derivatives attached to a glycerogalactoside moiety and are very common in algae [29,63], but they have not been reported for *V. lanosa* until now. Typically, glycerogalactolipids are constituents of membranes in plant plastids and other organisms performing photosynthesis [64]. Apart from their role in photosynthesis, they may also be involved in radical scavenging and cryoprotection [65] and exert haemolytic activity in organisms like oysters or fish [29,30].

Finally, the major MAAs in *V. lanosa* were identified, confirming the presence of porphyra-334 (**17**), the most abundant MAA in this alga, and palythine (**19**) [15]. Compound **18**, which was suspected to be palythinol based on its HPLC and UV data [15], was instead found to be aplysiapalythine A.

## 4. Materials and Methods

### 4.1. Plant Material and Chemicals

Algal material of *V. lanosa* was collected, identified and kindly provided as a gift by Nutramara Ltd. in March (VL1, 700 g) and October (VL2, 900 g) 2020 from Dungloe Bay, West Donegal, Ireland. Voucher specimens of both collection series (IPBP524 and IPBP528, respectively) were deposited at the Institute of Pharmaceutical Biology and Phytochemistry, University of Münster, Germany. Both batches of algae were kept separately during extraction and fractionation.

If not stated otherwise, all chemicals were purchased from VWR (Darmstadt, Germany). The solvents used for the analytical and preparative work were of analytical grade.

### 4.2. General Analytical Methods

Analytical UPLC for the purity assessment of the compounds and method development for preparative HPLC was generally carried out on Acquity™ Ultra Performance LC (UPLC), PDA λe Detector and QDa™ Detector autosampler, in-line degasser and Waters Empower 3^®^ Software (Waters, Milford, MA, USA). For separation, a binary gradient of 0.1% formic acid (A) and acetonitrile/0.1% formic acid (B) at 0.5 mL/min was used in a RP-18 stationary phase (HSS T3, 1.8 μm, 2.1 × 100 mm) at 40 °C. The separation of MAA was carried out in a HILIC stationary phase (Phenomenex Kinetex HILIC, 1.7 µm, 100 × 2.1 mm) at 40 °C using a binary gradient of 5 mM ammonium acetate pH 6.7 (A) and acetonitrile (B) at 0.5 mL/min, modified from [66]: *t*_0min_ 90% B, *t*_1min_ 90% B, *t*_4min_ 83% B, *t*_9min_ 83% B, *t*_11min_ 50% B, *t*_12.5min_ 50% B, *t*_13min_ 90% B and *t*_20min_ 90% B.

Thin-layer chromatography (TLC) for analytical purposes was performed on silica gel plates 60 F_254_ (0.2 mm; Merck, Darmstadt, Germany) using ethyl acetate/water/formic acid (90:5:5 *v/v/v*) as the standard mobile phase. Visualization of the compounds was achieved under UV light (254 nm or 365 nm, resp.) and at daylight after spraying with thymol/sulfuric acid reagent, followed by heating the plate to approximately 105 °C for bromophenolic compounds and for MAA by spraying with ninhydrin.

NMR spectra were recorded on an Agilent DD2 spectrometer (Agilent Technologies, Santa Clara, CA, USA) at 600 MHz (^1^H) or 150 MHz (^13^C). Depending on their solubility, samples were dissolved in the respective solvents as follows: **22** in chloroform-*d*_1_ (7.26; 77.16 ppm); **1**, **2**, **8**, **9**, **14**, **16**, **20**, **21** and **23** in methanol-*d*_4_ (3.31; 49.00 ppm); **3**, **5** and **6** in acetone-*d*_6_ (2.84; 206.26 ppm) and **4**, **10**–**13**, **15** and **17**–**19** in water-*d*_2_ (4.80 ppm). Chemical shifts were referenced to the respective residual solvent signals (^1^H and ^13^C shifts in brackets). Compounds **7** and **15** were dissolved in a 1:1 (*v/v*) mixture of water-*d*_2_ and methanol-*d*_4_ (experimentally determined shifts relative to TMS: 4.77 ppm (water-*d*_2_), 3.32 and 48.97 ppm (methanol-*d_4_*)). For **7** and **15**, ^1^H NMR (only **15**) and 2D spectra (**7** and **15**) were recorded utilizing WET signal suppression.

Analysis by UPLC-qTOF-MS was carried out as follows: Separation was performed on a Dionex Ultimate 3000 RS Liquid Chromatography System (Thermo Fisher Scientific, Waltham, MA, USA) over a Dionex Acclaim RSLC 120, C18 column (2.1 × 100 mm, 2.2 µm) with a binary gradient (A: water with 0.1% formic acid; B: acetonitrile with 0.1% formic acid) at 0.4 mL/min: t_0min_ 5% B, t_0.4min_ 5% B, t_9.9min_ 100% B, t_15min_ 100% B, t_15.1min_ 5% B and t_20min_ 5% B. The injection volume was 2 or 5 µL. Eluted compounds were detected using a Dionex Ultimate DAD-3000 RS over a wavelength range of 200–400 nm and a Bruker Daltonics micrOTOF-QII time-of-flight mass spectrometer equipped with an Apollo electrospray ionization source in positive or negative mode (depending on the respective substance) at 3 Hz over a mass range of *m/z* 50–1500 using the following instrument settings: nebulizer gas nitrogen, 3.5 bar; dry gas nitrogen, 9 L/min, 180 °C (positive mode) or 200 °C (negative mode); capillary voltage, 4500 V; end plate offset, −500 V; collision energy, +3 eV or −8 eV; transfer time, 100 µ and pre-pulse storage, 6 µs; the collision energy and collision RF settings were combined to the single spectrum of 1650 (positive mode) or 2483 (negative mode) summations. MS/MS scans were triggered by AutoMS2 settings within a range of *m/z*: 200–1500. Internal dataset calibration (HPC or enhanced quadratic mode) was performed for each analysis using the mass spectrum of a 10-mM solution of sodium formiate in isopropanol–water-formic acid–1M NaOH solution (50 + 50 + 0.2 + 1) that was infused during LC re-equilibration using a divert valve equipped with a 20-µL sample loop.

Simulation of NMR spectra and generation of IUPAC names was performed with ChemDraw Ver. 21.0, PerkinElmer Informatics Inc. 2022 (Waltham, MA, USA).

Optical rotations were measured using Autopol V (Rudolph analytical research) at 20 °C and 589 nm. The compound concentrations ranged from 0.1 to 0.6 g/100 mL, and the solvents used were methanol, water and methanol/water (1:1 *v*/*v*).

### 4.3. Hydrolysis of Glycosides

Mono- and disaccharides were hydrolyzed as described by Albersheim et al. [67]. Trifluoroacetic acid (TFA) was removed by at least three washing steps with 2 mL MeOH 50%. Hydrolyzed sugars were identified by TLC (n-propanol/water/EtOH 7/2/1 (*v*/*v*), visualization with thymol sulfuric acid after heating to 105 °C) compared to the reference compounds (concentration: 1 mg/mL).

### 4.4. Capillary Zone Electrophoresis (CZE)

The derivatization of the carbohydrates for analysis by CZE electrophoresis was conducted as described by Noe and Freissmuth [68]. CZE equipment: Beckman Coulter P/ACE MDQ (Beckman Coulter, Brea, CA, USA); fused silica capillary, 70/77 cm × 50 μm i.d.; running buffer, 50 mM Na_2_B_2_O_7_ pH 10.3, MeCN 4.4 mol/L added; injection, 5−10 s at 0.5 psi; voltage, 30 kV; detection, λ = 200 nm; software, 32 Karat software version 5.0 (Beckman Coulter).

### 4.5. Extraction and Fractionation

Air-dried and ground algae were extracted five times for 20 min using methanol/water (8:2, *v/v*) (3.75 L/500 g) in an ultrasonic bath. The crude extract was filtered and lyophilized after evaporation of the organic solvent, yielding M1 (122.52) and M2 (148.07 g). Subsequent defatting by stirring five times with 1.25 L petroleum ether/100 g of crude extract for 10 min yielded the defatted extracts Md1 (96.11 g) and Md2 (142.21 g). All extracts and fractions were stored at −20 °C.

#### 4.5.1. Solvent Partitioning of the Extracts

Twenty grams of defatted extract were sequentially partitioned between 1 L water and 1 L ethyl acetate. Each partitioning step was repeated four times with fresh ethyl acetate, yielding the ethyl acetate fractions E1 (1.184 g) and E2 (1.767 g) and the aqueous fractions W1 (80.18 g) and W2 (131.44 g).

#### 4.5.2. Fast Centrifugal Partition Chromatography (FCPC)

FCPC was performed using the following system: SCPC-250 (Gilson, Middleton, WI, USA); pump: AZURA P 4.1S (KNAUER GmbH, Berlin, Germany); injector: 10-mL injection loop, Rheodyne (Oak Harbor, WA, USA); mobile phase: EtOAc/hexane 3:7 (*v*/*v*), stationary phase: MeOH/water 7:3 (*v*/*v*); ascending mode, flow 10 mL/min, 1600 rpm, fraction size: 20 mL. Six hundred and fifty milligrams of sample per run were dissolved in 7 mL of the mobile phase. The fraction analysis by TLC led to fractions A–J (E1) and A II–G II (E2). Four hundred and fifty milligrams of fraction E2 (G II), representing the extruded stationary phase, was further fractionated via FCPC (mobile phase: EtOAc/butanol 4/5:0/5 (*v*/*v*), stationary phase: water), yielding fractions G IIa–G IIe.

#### 4.5.3. Medium-Pressure Liquid Chromatography (MPLC)

Twenty grams of the aqueous fraction W1 were separated by MPLC on a RP-18 stationary phase (RP-18, 18–32 µm, 100 Å, 36 × 500 mm (BESTA Technik, Wilhelmsfeld, Germany), flow rate 5 mL/min, step gradient MeOH 5%/HCOOH 0.1% (525 mL) → MeOH 25%/HCOOH 0.1% (300 mL) → MeOH 50%/HCOOH 0.1% (300 mL) → MeOH (900 mL min), fraction size 15 mL. Two grams of samples dissolved in 10 mL of water were injected per run. Every second fraction was spotted on a TLC plate and detected without development, yielding fractions Aq I–Aq V, of which Aq III corresponded to pure compound **4** (107 mg).

#### 4.5.4. Sugar Precipitation and Ion Exchange Chromatography

To obtain an MAA-enriched fraction, the polysaccharides were precipitated in the first step. Therefore, 50 g of W2 dissolved in 500 mL of water were added dropwise to 2 L of ice-cold ethanol while stirring. After stirring overnight and centrifugation (3000× *g*, 15 min), the residual polysaccharide fraction WP2 (9.6 g) and the polysaccharide-depleted supernatant WM2 (38.5 g) were obtained.

Ten grams of WM2 were then subjected to fractionation by ion exchange chromatography (Amberlite™ IR120 (H) resin, 40 × 550 mm, flow 4 mL/min) using a pH step gradient of 0.1 M citric acid and 0.2 M phosphate buffer [69]: pH 2.6 (700 mL) →pH 7.0 (700 mL) → pH 8.0 (2.7 L), fraction size 12 mL. TLC analysis of the fractions led to Amb1–Amb14.

#### 4.5.5. Preparative HPLC

Preparative HPLC was carried out using Waters Quaternary Gradient Module 2545, Photodiode Array Detector 2998, Autosampler 2707, Prep Degasser and Fraction Collector III. Software: Waters ChromScope v1.40 Beta (Waters, Milford, MA, USA). Stationary phase: Nucleodur^®^ C18 HTec, 5 µm, 250 × 21 mm and mobile phase: binary gradient of water (A) and acetonitrile (B) at a flow rate of 15 mL/min. For fractions Aq IV, Aq Iva and Aq IVb, the addition of 0.1% trifluoroacetic acid was necessary. For fraction Amb9, the stationary phase was a Modulo-card Uptisphere 6 µm, diol, 250 × 21.2 mm, and the mobile phase consisted of a binary gradient of 5 mM ammonium acetate buffer pH 6.7 (A) and acetonitrile (B). The gradients were adapted to each subfraction individually, and the purity was assessed by UPLC. Pure compounds were obtained from the respective subfractions by preparative HPLC as follows: Fraction D: **2** (1.3 mg); fractions G and H: **5** (1.7 and 2.9 mg, respectively); fraction J: **8** (0.4 mg); fraction C II: **3** (1.0 mg) and **5** (2.3 mg); fraction D II: **1** (5.2 mg) and **5** (1.2 mg); fraction F II: **6** (2.8 mg), **20** (0.6 mg), **21** (2.7 mg) and **22** (22.8 mg); fraction G IIa: **9** (5.7 mg), **20** (0.7 mg) and **23** (1.6 mg); fraction Aq IV: **11** (11.8 mg) and **13** (9.5 mg); fraction Aq IVa: **2** (2.8 mg), **12** (5.1 mg), **14** (3.7 mg) and **15** (25.5 mg); fraction Aq IVb: **7** (3.2 mg), **10** (11.5 mg) and **16** (1.1 mg) and fraction Amb9: **17** (5 mg), **18** (1 mg) and **19** (1 mg).

### 4.6. Structure Elucidation of Isolated Compounds

Spectroscopic data (UV spectra, NMR spectra and optical rotation) and HR-ESI-MS were used for structure elucidation. NMR shifts of the known compounds (**1**–**6**, **8**–**11** and **1722**) were compared to the published literature and are reported as the Supplementary Materials [70]. Their respective IUPAC names are given in brackets.

***4-Sulfo-7-dimethylsulfonium lanosol (2,3-dibromo-4-((dimethylsulfonio)methyl)-6-hydroxyphenyl sulfate) (7):*** orange amorphous solid; UV (MeCN, H_2_O) *λ*_max_ 212, 300 nm. ^1^H NMR and ^13^C NMR assignments are given in Table 1. HR(+)ESI-MS *m/z* 424.8411/422.8437/420.8463 (56:100:48) [M + H]^+^ (calcd. for C_9_H_11_^79^Br_2_O_5_S_2_^+^, 420.8409). Observed adducts and fragments: 446.8255/444.8231/442.8319 (1.3:2.3:1.1) [M + Na]^+^, 441.8606/439.8681/437.8642 (1.1:1.5:0.7) [M + NH_4_]^+^, 362.8190/360.8213/358.8278 (1.0/2.0/1.0) [M – C_2_H_6_S]^+^, 344.8835/342.8857/340.8882 (11:20:10) [M – SO_3_]^+^, 304.8454/302.8476/300.8512 (2.0:3.7:2.0), 282.8634/280.8657/278.8681 (11:21:11) [C_7_H_5_Br_2_O_2_]^+^; HR(-)ESI-MS *m/z* 422.8228/420.8246/418.8255 (54:100:47) [M – H]^−^ (calcd. for C_9_H_9_^79^Br_2_O_5_S_2_^+^, 418.8264). Observed adducts and fragments: 1270.4775/1268.4796/1266.4811/1264.4819/1262.4938/1260.4836/1258.4411 (1.8:3.6:6.7:6.4:5.4:2.3:0.5) [3M – H]^−^, 846.6488/844.6522/842.6511/840.6541/838.6606 (3.2:7.5:10:7.1:1.9) [2M – H]^−^, 408.8086/406.8088/404.8096 (19:43:37) [M – CH_3_]^−^, 406.8088/404.8096/402.8141 (43:37:11) [M – CH_4_]^-^, 392.7819/390.7742/388.7762 (2.0:3.2:1.7) [M – C_2_H_6_]^−^, 374.8163/372.8203/370.8228 (3.0:5.1:2.2) [M – CH_4_S]^−^, 360.8072/358.8057/356.8041 (5.0:9.9:5.1) [M – C_2_H_6_S]^-^, 342.8753/340.8838/338.8897 (1.4:4.4:2.8) [M – SO_3_]^−^, 328.8621/326.8742/324.8738 (1.1:5.5:4.1) [M – CH_2_SO_3_]^−^, 298.9073/296.9085/294.8619 (3.3:3.7:2.4), 294.8619/292.8643/290.8664 (2.4:4.2:2.1), 160.8731/158.8745 (3.4:3.0).

***3,5-Dibromo-L-tyrosine (2-amino-3-(3,5-dibromo-4-hydroxyphenyl)propanoic acid) (12):*** red amorphous solid; [α]^20^_D_ -67.7 (*c* 0.1, H_2_O); UV (MeCN, H_2_O) *λ*_max_ 208, 288 nm. ^1^H and ^13^C NMR, see Table 2. HR(+)ESI-MS *m/z* 341.8944/339.8966/337.8983 (50:100:53) [M + H]^+^ (calcd. for C_9_H_10_^79^Br_2_NO_3_^+^, 337.9022). Observed adducts and fragments: 682.7802/680.7818/678.7841/676.7853/674.7884 (0.5:1.8:2.6:1.9:0.5) [2M + H]^+^, 324.8681/322.8694/320.8712 (1.4:2.7:1.4) [M – NH_3_]^+^, 295.8888/293.8911/291.8632 (3.1:6.6:3.3) [M – HCOOH]^+^, 282.8589/280.8590/278.8616 (0.1:0.2:0.1) [C_7_H_5_Br_2_O_2_]^+^.

***3-Bromo-5-sulfo-L-dihydroxyphenylalanine (2-amino-3-(3-bromo-4-hydroxy-5-(sulfooxy)phenyl)propanoic acid) (13):*** off-white amorphous solid; [α]^20^_D_ -10.4 (*c* 0.2, H_2_O); UV (MeCN, H_2_O) *λ*_max_ 200, 284 nm. ^1^H and ^13^C NMR, see Table 2. HR(-)ESI-MS *m/z* 355.9304/353.9326 (100:99.7) [M – H]^−^ (calcd. for C_9_H_9_^79^BrNO_7_S^-^, 353.9289). Observed adducts and fragments: 734.8495/732.8498/730.8502 (2.0:3.0:1.5) [2M + Na – H]^−^, 712.8664/710.8679/708.8694 (8.6:14:6.7) [2M + H]^−^, 632.9083/630.9133/628.9128 (1.2:2.1:1.1) [2M – SO_3_]^−^, 377.9113/375.9135 (3.3:3.0) [M + Na – H]^−^, 275.9731/273.9765 (2.1:2.3) [M – SO_3_]^−^.

***3-Bromo-6-lanosyl dihydroxyphenylalanine (2-amino-3-(5-bromo-2-(2,3-dibromo-4,5-dihydroxybenzyl)-3,4-dihydroxyphenyl)propanoic acid) (14):*** reddish-brown amorphous solid; [α]^20^_D_-5.7 (*c* 0.1, MeOH); UV (MeCN, H_2_O) *λ*_max_ 212, 288 nm. ^1^H and ^13^C NMR, see Table 2. HR(-)ESI-MS *m/z* 557.8233/555.8243/553.8265/551.8285 (31:94:100:36) [M – H]^−^ (calcd. for C_16_H_13_^79^Br_3_NO_6_^−^, 551.8298). Observed adducts and fragments: 1116.6650/1114.6559/1112.6563/1110.6609/1108.6574/1106.6631/1104.6636 (6.0:23:57:71:55:23:4.1) [2M – H]^−^, 475.9023/473.9005/471.9021 (29:55:25) [M – HBr]^−^, 401.8730/399.8681/397.8646 (2.8:7.5:3.9), 285.9549/283.9564 (15:16).

***3-(6′-lanosyllanosyl)-tyrosine (2-amino-3-(3-(2,3-dibromo-6-(2,3-dibromo-4,5-dihydroxybenzyl)-4,5-dihydroxybenzyl)-4-hydroxyphenyl)propanoic acid)(15):*** brown amorphous solid; [α]^20^_D_ +2.8 (*c* 0.6, MeOH/H_2_O (1:1, *v/v*)); UV (MeCN, H_2_O) *λ*_max_ 212, 284 nm. ^1^H and ^13^C NMR, see Table 2. HR(-)ESI-MS *m/z* 743.7806/741.7758/739.7778/737.7803/735.7761 (16:72:100:69:21) [M – H]^−^ (calcd. for C_23_H_18_^79^Br_4_NO_7_^-^, 735.7822). Observed adducts and fragments: 1488.5855/1486.5671/1484.5609/1482.5653/1480.5649/1478.5662/1476.5600/1474.5634/1472.5691 (1.8:7.0:16:35:44:32:14:3.9:1.1) [2M – H]^−^, 661.8463/659.8541/657.8556/655.8593 (18:48:49:21) [M – HBr]^−^, 571.8429/569.8419/567.8442/565.8438 (9.5:30:33:9.3), 312.8692/310.8778/308.8782 (8.3:16:6.5).

***5-Sulfovertebratol (2-amino-5-(3-(2,3-dibromo-4-hydroxy-5-(sulfooxy)benzyl)ureido)pentanoic acid) (16):*** brown amorphous solid; UV (MeCN, H_2_O) *λ*_max_ 208, 291 nm. ^1^H and ^13^C NMR, see Table 1. HR(+)ESI-MS *m/z* 537.9224/535.9246/533.9257 (53:100:47) [M + H]^+^ (calcd. for C_13_H_18_^79^Br_2_N_3_O_8_S^+^, 533.9176). Observed adducts and fragments: 1074.8353/1072.8343/1070.8437/1068.8449/1066.8397 (2.2:7.3:9.1:5.8:1.5) [2M + H]^+^, 559.9050/557.9060/555.9135 (1.1:2.1:1.4) [M + Na]^+^, 554.9228/552.9428/550.9326 (0.8:0.7:0.3) [M + NH_4_]^+^, 520.8884/518.8865/516.9051 (0.6:0.9:0.6) [M – NH_3_]^+^, 510.8561/508.8674/506.8660 (0.6:0.8:0.5), 457.9631/455.9705/453.9645 (1.7:3.0:1.9) [M – SO_3_, Vertebratol]^+^.

***(5*Z*,8Z,11*Z*,14*Z*,17*Z*)-Eicosapentaenoic acid 3′-[(6***″***-*O*-α-galactopyranosyl-β-D-galactopyranosyl)]-1-glycerol ester*** (2-hydroxy-3-(((2*R*,3*R*,4*S*,5*R*,6*R*)-3,4,5-trihydroxy-6-((((*2S*,3*R*,4*S*,5*R*,6*R*)-3,4,5-trihydroxy-6-(hydroxymethyl)tetrahydro-2H-pyran-2-yl)oxy)methyl)tetrahydro-2H-pyran-2-yl)oxy)propyl (5*Z*,8*Z*,11*Z*,14*Z*,17*Z*)-icosa-5,8,11,14,17-pentaenoate) ***(23):*** sticky yellow solid; UV (MeCN, H_2_O) *λ*_max_ 224 nm. ^1^H NMR (CD_3_OD, 600 MHz) δ 5.44–5.29 (10H, m, H-5, H-6, H-8, H-9, H-11, H-12, H-14, H-15, H-17, H-18), 4.26 (2H, d, *J* = 7.5 Hz, H-1″), 4.19–4.13 (2H, m, H-1′), 4.02–3.98 (1H, m, H-2′), 3.93–3.91 (1H, m, H-6″a), 3.91–3.89 (1H, m, H4′″), 3.88 (1H, s, H-3′a), 3.88 (1H, d, *J* = 5.4 Hz, H-4″), 3.86 (1H, d, *J* = 5.3 Hz, H-5′’’), 3.79 (1H, dd, *J* = 10.1, 3.7 Hz, H-2′″), 3.77 (1H, d, *J* = 8.1 Hz, H-5″), 3.75 (1H, d, *J* = 6.9 Hz, H-3′″), 3.74–3.71 (2H, m, H-6′″), 3.70 (1H, d, *J* = 3.7 Hz, H-6″b), 3.69–3.65 (1H, m, 3′b), 3.55 (1H, dd, *J* = 9.8, 7.5 Hz, H-2″), 3.51 (1H, dd, *J* = 9.7, 3.4 Hz, H-3″), 2.86 (8H, dt, *J* = 16.3, 5.3 Hz, H-7, H-10, H-13, H-16), 2.39 (2H, t, *J* = 7.4 Hz, H-3), 2.17–2.13 (2H, m, H-4), 2.12–2.07 (2H, m, H-19), 1.70 (2H, p, *J* = 7.4 Hz, H-3), 1.01–0.97 (3H, m, H-20). The coupling constant of H-1″ was determined in acetone as the solvent signal of methanol at δ_H_ 4.87 that interfered with its measurement: ^1^H NMR (acetone-*d*_6_, 600 MHz) δ 4.84 (1H, d, *J* = 3.8 Hz, H-1′″); ^13^C NMR (CD_3_OD, 151 MHz) δ 175.25 (C-1), 132.82 (C-18), 130.04, 129.91 (C-5, C-6), 129.47, 129.25, 129.20, 129.13, 129.11, 128.94, 128.20 (C-8, C-9, C-11, C-12, C-14, C-15, C-17), 105.33 (C-1″), 100.56 (C-1′″), 74.67 (C-3″), 74.57 (C-5″), 72.57 (C-5′″), 72.53 (C-2″), 72.12 (C-3′), 71.47 (C-3′″), 71.06 (C-4′″), 70.24 (C-2′″), 70.10 (C-4″), 69.67 (C-2′), 67.78 (C-6″), 66.60 (C-1′), 62.75 (C-6′″), 34.34 (C-2), 27.56 (C-4), 26.57, 26.55 (C-7, C-10, C-13), 26.44 (C-16), 25.90 (C-3), 21.51 (C-19), 14.67 (C-20); HR(+)ESI-MS *m/z* 701.3766 (14) [M + H]^+^ (calcd. for C_35_H_57_O_14_^+^, 701.3766). Observed adducts and fragments: 1423.7381 (17) [2M + Na]^+^, 1418.7820 (12) [2M + NH_4_]^+^, 1401.7559 (24) [2M + H]^+^, 723.3584 (69) [M + Na]^+^, 718.4033 (71) [M + NH_4_]^+^, 683.3676 (22) [M – H_2_O]^+^, 539.3223 36) [M – Gal]^+^, 521.3117 (11) [M – Gal – H_2_O]^+^, 377.2684 (100) [M – 2Gal]^+^, 285.2208 (24) (fatty acid oxocarbenium ion, [C_20_H_29_O]^+^), 267.2107 (8.2) (fatty acid carbenium ion [C_20_H_27_]^+^).

## Figures and Tables

**Table 1:** ^1^H- (600 MHz) and ^13^C-NMR (151 MHz) data of the new lanosol derivatives **7** and **16** in D_2_O/CD_3_OD 1:1 (*v/v*).

	7		16	
Pos.	^13^C	^1^H	^13^C	^1^H
1	127.78 (C)	-	131.44 ^a^ (C)	-
2	118.86 (C)	-	114.34 ^a^ (C)	-
3	125.05 (C)	-	112.70 ^a^ (C)	-
4	141.40 (C)	-	145.62 ^a^ (C)	-
5	151.20 (C)	-	146.80 ^a^ (C)	-
6	121.76 (CH)	7.25 (s)	115.86 (CH)	6.93 (s)
7	50.16 (CH)	4.80 (s)	75.85 (CH_2_)	4.43 (s)
8	25.28 (2 CH_3_)	2.97 (s)	162.73 ^a^ (CO)	-
1′			172.22 ^a^ (COOH)	-
2′			53.63 (CH)	3.98 (t, 6.4)
3′			28.78 (CH_2_)	2.00 (d, 4.1) 1.90–1.85 (m)
4′			27.07 (CH_2_)	1.66 (m)
5′			39.63 ^b^ (CH_2_)	3.20–3.17 (m) 3.16–3.11 (m)

^a^ Shifts obtained from the HMBC spectrum. ^b^ Shifts obtained from the HSQC spectrum.

**Table 2:** ^1^H- (600 MHz) and ^13^C- NMR (151 MHz) data of tyrosine derivatives **12**–**15**.

	12 in D_2_O		13 in D_2_O		14 in CD_3_OD		15 in D_2_O/CD_3_OD 1:1 (*v/v*)	
Pos.	^13^C	^1^H	^13^C	^1^H	^13^C	^1^H	^13^C	^1^H
COOH	172.64 (*C*OOH)	-	172.18 (*C*OOH)	-	172.02 ^a^ (*C*OOH)	-	173.95 (*C*OOH)	-
α	55.36 (CH)	4.19 (dd, 7.6, 5.7)	54.95 (CH)	4.30 (ddd, 7.9, 5.4, 1.0)	55.36 (CH)	3.80 (dd, 8.9, 6.4)	58.32 (CH)	3.71 (d, 4.5)
β	35.25 (CH_2_)	3.23 (dd, 14.7, 5.6) 3.11 (dd, 14.8, 7.6)	35.37 (CH_2_)	3.29 (dd, 15.0, 5.6) 3.13 (dd, 15.0, 7.9)	34.70 (CH_2_)	3.08 (dd, 14.7, 6.2) 2.79 (dd, 14.7, 8.8)	36.85 (CH_2_)	2.70 (dd, 14.8, 9.0) 3.01 (dd, 14.7, 4.8)
1	130.25 (C)	-	128.05 (C)	-	126.35 (C)	-	126.97 (C)	-
2	134.10 (CH)	7.49 (s)	132.12 (CH)	7.40 (dd, 2.2, 1.1)	125.50 (CH)	7.01 (s)	129.49 (CH)	6.24 (d, 2.2)
3	112.20 (C)	-	111.84 (C)	-	109.97 (C)	-	126.47 (C)	-
4	150.14 (C)	-	146.47 (C)	-	143.67 (C)	-	154.23 (C)	-
5	112.20 (C)		140.47 (C)	-	147.44 (C)	-	115.88 (CH)	6.74 (d, 8.2)
6	134.10 (CH)	7.49 (s)	123.92 (CH)	7.29 (dd, 2.2, 1.1)	128.62 (C)	-	128.51 (CH)	6.89 (d, 8.1)
1′					132.49 (C)	-	133.16 (C)	-
2′					116.62 (C)	-	119.29 (C)	-
3′					114.45 (C)	-	113.94 (C)	-
4′					143.93 (C)	-	143.33 (C)	-
5′					146.29 (C)	-	145.39 (C)	-
6′					115.01 (CH)	6.14 (s)	128.45 (C)	-
7′					34.17 (CH_2_)	4.10 (d, 17.6) 4.01 (d, 17.5)	34.83 (CH_2_)	4.01 (s)
1′’							132.74 (C)	-
2′’							116.63 (C)	-
3′’							114.25 (C)	-
4′’							145.28 (C)	-
5′’							142.37 (C)	-
6′’							115.59 (CH)	6.17 (s)
7′’							34.92 (CH_2_)	3.96 (s)

^a^ Shifts obtained from the HMBC spectrum.

## Data Availability

The data are contained within the article and the Supplementary Materials.

## Supplementary Materials

The following supporting information can be downloaded at: https://www.mdpi.com/article/10.3390/md20070420/s1. Analytical data used for the structure elucidation of **1**–**6**, **8**–**11** and **17**–**22**. Table S1. ^1^H- and ^13C^-NMR data of **20**–**23**. Figure S1. (a–e) One- and two-dimensional NMR spectra of **7**. Figure S2. (a–e) One- and two-dimensional NMR spectra of **12**. Figure S3. (a–e) One- and two-dimensional NMR spectra of **13**. Figure S4. (a–e) One- and two-dimensional NMR spectra of **14**. Figure S5. (a–e) One- and two-dimensional NMR spectra of **15**. Figure S6. (a–e) One- and two-dimensional NMR spectra of **16**. Figure S7. (a–f) One- and two-dimensional NMR spectra of **23**. Figure S8. (a–c) HR-ESI-MS spectra of **7**. Figure S9. HR-(+)ESI-MS spectrum of **12**. Figure S10. HR-(-)ESI-MS spectrum of **13**. Figure S11. HR-(-)ESI-MS spectrum of **14**. Figure S12. HR-(-)ESI-MS spectrum of **15**. Figure S13. HR-(+)ESI-MS spectrum of **16**. Figure S14. HR-(+)ESI-MS spectrum of **23**. Figure S15. ^1^H-NMR spectrum of **1** (methanol-*d*_4_, 289 K, 600 MHz). Figure S16. ^1^H-NMR spectrum of **2** (methanol-*d*_4_, 289 K, 600 MHz). Figure S17. ^1^H-NMR spectrum of **3** (acetone-*d*_6_, 289 K, 600 MHz). Figure S18. ^1^H-NMR spectrum of **4** (water-*d*_2_, 289 K, 400 MHz). Figure S19. ^1^H-NMR spectrum of **5** (acetone-*d*_6_, 289 K, 600 MHz). Figure S20. ^1^H-NMR spectrum of **6** (acetone-*d*_6_, 289 K, 600 MHz). Figure S21. ^1^H-NMR spectrum of **8** (methanol-*d*_4_, 289 K, 600 MHz). Figure S22. ^1^H-NMR spectrum of **9** (methanol-*d*_4_, 289 K, 600 MHz). Figure S23. ^1^H-NMR spectrum of **10** (water-*d*_2_, 289 K, 600 MHz). Figure S24. ^1^H-NMR spectrum of **11** (water-*d*_2_, 289 K, 600 MHz). Figure S25. ^1^H-NMR spectrum of **17** (water-*d*_2_, 289 K, 600 MHz). Figure S26. ^1^H-NMR spectrum of **18** (water-*d*_2_, 289 K, 600 MHz). Figure S27. ^1^H-NMR spectrum of **19** (water-*d*_2_, 289 K, 600 MHz). Figure S28. ^1^H-NMR spectrum of **20** (methanol-*d*_4_, 289 K, 600 MHz). Figure S29. ^1^H-NMR spectrum of **21** (methanol-*d*_4_, 289 K, 600 MHz). Figure S30. ^1^H-NMR spectrum of **22** (chloroform-*d_1_*, 289 K, 600 MHz).
