# Supplementary material for: Amino Acid-Coupled Bromophenols and a Sulfated Dimethylsulfonium Lanosol from the Red Alga Vertebrata lanosa
Source: Mar Drugs. 2022 Jun 27;20(7):420. doi: 10.3390/md20070420 (PMC9322897; doi:10.3390/md20070420)

Supplementary Materials

Amino Acid Coupled Bromophenols and a Sulfated Dimethylsulfonium Lanosol from the Red Alga *Vertebrata lanosa*

Joshua Jacobtorweihen[1], Marthe Schmitt [1,2] and Verena Spiegler[1,*]

[1] Institute for Pharmaceutical Biology and Phytochemistry, University of Münster, Correnssstraße 48, 48149 Münster, Germany; E-mail: j.jacobtorweihen@uni-muenster.de

[2] SIGMA Clermont, 20 Avenue Blaise Pascal, TSA 62006, 63178 AUBIERE CEDEX, France; E-Mail : marthe.schmitt@sigma-clermont.fr

* Correspondence: verena.spiegler@uni-muenster.de

Analytical data used for the structure elucidation of **1** – **6**, **8** – **11** and **17** – **22**.

Table S1. ^{1}H- and ^{13C}-NMR data of **20** – **23**.

Figure S1. (a) – (e) 1D and 2D-NMR spectra of **7**.

Figure S2. (a) – (e) 1D and 2D-NMR spectra of **12**.

Figure S3. (a) – (e) 1D and 2D-NMR spectra of **13**.

Figure S4. (a) – (e) 1D and 2D-NMR spectra of **14**.

Figure S5. (a) – (e) 1D and 2D-NMR spectra of **15**.

Figure S6. (a) – (e) 1D and 2D-NMR spectra of **16**.

Figure S7. (a) – (f) 1D and 2D-NMR spectra of **23**.

Figure S8. (a) – (c) HR-ESI-MS spectra of **7**.

Figure S9. HR-(+)ESI-MS spectrum of **12**.

Figure S10. HR-(-)ESI-MS spectrum of **13**.

Figure S11. HR-(-)ESI-MS spectrum of **14**.

Figure S12. HR-(-)ESI-MS spectrum of **15**.

Figure S13. HR-(+)ESI-MS spectrum of **16**.

Figure S14. HR-(+)ESI-MS spectrum of **23**.

Figure S15. ^{1}H-NMR spectrum of **1** (methanol-d_4, 289 K, 600 MHz).

Figure S16. ^{1}H-NMR spectrum of **2** (methanol-d_4, 289 K, 600 MHz).

Figure S17. ^{1}H-NMR spectrum of **3** (acetone-d_6, 289 K, 600 MHz).

Figure S18. ^{1}H-NMR spectrum of **4** (water-d_2, 289 K, 400 MHz).

Figure S19. ^{1}H-NMR spectrum of **5** (acetone-d_6, 289 K, 600 MHz).

Figure S20. ^{1}H-NMR spectrum of **6** (acetone-d_6, 289 K, 600 MHz).

Figure S21. ^{1}H-NMR spectrum of **8** (methanol-d_4, 289 K, 600 MHz).

Figure S22. ^{1}H-NMR spectrum of **9** (methanol-d_4, 289 K, 600 MHz).

Figure S23. ^{1}H-NMR spectrum of **10** (water-d_2, 289 K, 600 MHz).

Figure S24. ^{1}H-NMR spectrum of **11** (water-d_2, 289 K, 600 MHz).

Figure S25. ^{1}H-NMR spectrum of **17** (water-d_2, 289 K, 600 MHz).

Figure S26. ^{1}H-NMR spectrum of **18** (water-d_2, 289 K, 600 MHz).

Figure S27. ^{1}H-NMR spectrum of **19** (water-d_2, 289 K, 600 MHz).

Figure S28. ^{1}H-NMR spectrum of **20** (methanol-d_4, 289 K, 600 MHz).

Figure S29. ^{1}H-NMR spectrum of **21** (methanol-d_4, 289 K, 600 MHz).

Figure S30. ^{1}H-NMR spectrum of **22** (chloroform-d_1, 289 K, 600 MHz).

Analytical data used for the structure elucidation of 1 – 6, 8 – 11 and 17 – 22. IUPAC names are given in brackets.

Lanosol (3,4-dibromo-5-(hydroxymethyl)benzene-1,2-diol) (1): grey brown amorphous solid; UV (MeCN, H_2O) λ_{max} 236, 292 nm; ^{1}H NMR (CD_3OD, 600 MHz) δ 7.01 (1H, s, H-6), 4.55 (2H, s, H-7); ^{13}C NMR (CD_3OD, 151 MHz) δ 146.44 (C-5), 144.77 (C-4), 134.27 (C-1), 114.89 (C-6), 114.24 (C-3), 114.05 (C-2), 65.66 (C-7); HR(+)ESI-MS *m/z* 298.8610 / 296.8643 / 294.8613 (1.9:3.4:1.4) [M + H]$^+$ (calcd. for $C_7H_5{}^{79}Br_2O_3^+$, 294.8600); Observed adducts and fragments: 320.8494 / 318.8455 / 316.8484 (23:36:14) [M + Na]$^+$, 315.8939 / 313.8897 / 311.8941 (1.9:3.1:1.4) [M + NH_4]$^+$, 282.8646 / 280.8668 / 278.8692 (48:100:54) [M – OH]$^+$.

Lanosol methyl ether (3,4-dibromo-5-(methoxymethyl)benzene-1,2-diol) (2): red brown amorphous solid; UV (MeCN, H_2O) λ_{max} 212, 292 nm; ^{1}H NMR (CD_3OD, 600 MHz) δ 6.93 (1H, s, H-6), 4.43 (2H, s, H-7), 3.40 (3H, s, H-8); ^{13}C NMR (CD_3OD, 151 MHz) δ 146.34 (C-5), 145.33 (C-4), 131.04 (C-1), 115.86 (C-6), 115.27 (C-2), 114.35 (C-3), 75.95 (C-7), 58.50 (C-8); HR(+)ESI-MS *m/z* 312.8741 / 310.8740 / 308.8765 (13:26:13) [M + H]$^+$ (calcd. for $C_8H_7{}^{79}Br_2O_3^+$, 308.8756); Observed adducts and fragments: 646.7428 /644.7415 / 642.7290 / 640.7248 / 638.7303 (4.3:5.4:5.4:2.0:0.8) [2M + Na]$^+$, 334.8644 / 332.8606 / 330.8587 (47:55:22)* [M + Na]$^+$, 329.9094 / 327.9044 / 325.8998 (5.5:5.3:2.6) [M + NH_4]$^+$, 282.8611 / 280.8628 / 278.8652 (51:100:51) [M – OCH_3]$^+$.

2,3-Dibromo-4,5-dihydroxyphenylacetic acid methyl ester (methyl 2-(2,3-dibromo-4,5-dihydroxyphenyl)acetate) (3): brown amorphous solid; UV (MeCN, H_2O) λ_{max} 208, 292 nm; ^{1}H NMR (acetone-d_6, 600 MHz) δ 6.96 (1H, s, H-6), 3.76 (2H, s, H-7), 3.64 (3H, s, H-9); ^{13}C NMR (acetone-d_6, 151 MHz) δ 171.32 (C-8), 145.51 (C-5), 144.52 (C-4), 128.14 (C-1), 118.13 (C-2), 117.30 (C-6), 113.74 (C-3), 52.12 (C-9), 42.84 (C-7); HR(+)ESI-MS *m/z* 342.8856 / 340.8873 / 338.8891 (48:96:49) [M + H]$^+$ (calcd. for $C_9H_9{}^{79}Br_2O_4^+$, 338.8892); Observed adducts and fragments: 706.7439 / 704.7442 / 702.7451 / 700.7448 / 698.7417 (4.1:18:29:25:11) [2M + Na]$^+$, 684.7643 / 682.7644 / 680.7641 / 678.7658 / 676.7760 (0.6:2.4:3.5:2.4:0.6) [2M + H]$^+$, 364.8680 / 362.8690 / 360.8691 (46:100:59) [M + Na]$^+$, 359.9076 / 357.9127 / 355.9129 (11:21:12) [M + NH_4]$^+$, 310.8596 / 308.5990 / 306.8595 (1.3:2.3:1.1) [M – OCH_3]$^+$, 298.8591 / 296.8607 / 294.9608 (2.3:4.9:2.7) [M – C_2H_4O]$^+$, 282.8635 / 280.8658 / 278.8679 (14:27:14) [$C_7H_5Br_2O_2$]$^+$.

4,7-Disulfolanosol (2,3-dibromo-5-hydroxy-4-(sulfooxy)benzyl hydrogen sulfate) (4): off white amorphous solid; UV (MeCN, H_2O) λ_{max} 208, 290 nm; ^{1}H NMR (D_2O, 400 MHz) δ 7.24 – 7.22 (1H, m, H-6), 5.14 (2H, dd, *J* = 2.1, 1.0 Hz, H-7); ^{13}C NMR (D_2O, 101 MHz) δ 149.39 (C-5), 138.37 (C-4), 134.99 (C-1), 122.58 (C-3), 117.82 (C-6), 115.54 (C-2), 70.66 (C-7); HR(+)ESI-MS *m/z* 477.8195 / 475.8196 / 473.8211 (42/75/37) $[M + NH_4]^+$ (calcd. for $C_7H_{10}{}^{79}Br_2NO_9S_2{}^+$, 473.8158); Observed adducts and fragments: 499.7839 / 497.7911 / 495.8012 $[M + Na + NH_4]^+$, 494.8432 / 492.8413 / 490.8360 $[M + 2NH_4]^+$, 419.8363 / 417.8416 / 415.8521, 282.8641 / 280.8658 / 278.8682 $[C_7H_5Br_2O_2]^{+.}$

7,7′-Bis-lanosol ether (5,5′-(oxybis(methylene))bis(3,4-dibromobenzene-1,2-diol)) (5): dark brown amorphous solid; UV (MeCN, H_2O) λ_{max} 212, 292 nm; ^{1}H NMR (acetone-*d*$_6$, 600 MHz) δ 7.35 (1H, s, H-6 / H-6'), 4.59 (3H, s, H-7 / H-7'); ^{13}C NMR (acetone-*d*$_6$, 151 MHz) δ 146.09 (C-5 / C-5'), 145.07(C-4 / C-4'), 131.06 (C-1 / C-1'), 115.61 (C-6 / C-6'), 114.04 (C-2 / C-2'), 113.38 (C-3 / C-3'), 73.21 (C-7 / C-7'); HR(+)ESI-MS *m/z* 580.7167 / 578.7314 / 576.7239 / 574.7550 (0.8:1.4:1.1:0.5) $[M + H]^+$ (calcd. for $C_{14}H_{11}{}^{79}Br_4O_5{}^+$, 574.7334); Observed adducts and fragments: 1183.4340 / 1181.4486 / 1179.4500 / 1177.4648 / 1175.4793 / 1173.4813 / 1171.4851 / 1167.4857 / 1165.5287 (2.1:5.0:7.2:19:20:16:8.3:2.8:0.5) $[2M + NH_4]^+$, 599.7496 / 597.7535 / 595.7583 / 593.7587 / 591.7598 (18:65:100:71:20) $[M + NH_4]^+$, 564.7177 / 562.7170 / 560.7175 / 558.7164 / 556.7151 (5:11:13:7) $[M – H_2O]^+$, 299.8891 / 297.8900 / 295.8935 (10:20:10) $[C_7H_4Br_2O_2 + NH_4]^+$, 282.8626 / 280.8642 / 278.8660 (48:97:47) $[C_7H_5Br_2O_2]^+$.

2′-Lanosyl-3′-bromo-5′,6′-dihydroxybenzyl alcohol (3,4-dibromo-5-(2-bromo-3,4-dihydroxy-6-(hydroxymethyl)benzyl)benzene-1,2-diol) (6): grey brown amorphous solid; UV (MeCN, H_2O) λ_{max} 212, 288 nm; ^{1}H NMR (acetone-*d*$_6$, 600 MHz) δ 7.10 (1H, s, H-6'), 6.08 (1H, s, H-6), 4.41 (2H, s, H-7'), 4.12 (2H, s, H-7); ^{13}C NMR (151 MHz, acetone-*d*$_6$) δ 145.42 (C-5), 145.00 (C-5'), 143.51 (C-4), 142.80 (C-4'), 134.42 (C-1'), 132.37 (C-1), 128.25 (C-2'), 116.34 (C-2), 115.07 (C-6'), 114.86 (C-3'), 114.78 (C-6), 113.67 (C-3), 62.70 (C-7'), 39.20 (C-7); HR(+)ESI-MS *m/z* 519.8480 / 517.8512 / 515.8520 / 513.2528 (16:49:52:22) $[M + NH_4]^+$ (calcd. for $C_{14}H_{15}{}^{79}Br_3NO_5{}^+$, 513.8495); Observed adducts and fragments: 1021.6358 / 1019.6357 / 1017.6588 / 1015.6678 / 1013.6700 / 1011.6686 / 1009.6620 (3.7:4.0:4.4:4.6:3.5:1.6:0.7) $[2M + NH_4]^+$, 502.8427 / 500.8420 / 498.8421 / 496.8281 (1.5:5.5:5.9:3.0) $[M + H]^+$, 501.8413 / 499.8417 / 497.8438 / 495.8459 (10:32:31:12) $[M – H_2O + NH_4]^+$, 484.8131 / 482.8148 / 480.8154 / 478.8149 (31:95:100:41) $[M – H_2O]^+$, 403.8958 / 401.8974 / 399.9001 (17:35:18) $[M – 2H_2O – HBr + NH_4]^+$, 402.8907 / 400.8912 / 398.8914 (15:22:10) $[M – H_2O – HBr]^+$, 282.8652 / 280.8677 / 278.8730 (2.4:4.2:2.1) $[C_7H_5Br_2O_2]^+$.

Rhodomelol (3-(2,3-dibromo-4,5-dihydroxybenzyl)-3,3a,6-trihydroxytetrahydrofuro[3,2-b]furan-2(3H)-one) (8): red brown oil; UV (MeCN, H_2O) λ_{max} 208, 292 nm; ^{1}H NMR (CD_3OD 600 MHz) δ 7.05 (1H, s, H-6), 4.60 (2H, s, H-6'a), 4.42 (1H, dd, *J* = 6.2, 3.7 Hz, H-6'), 4.24 (1H, dd, *J* = 9.8, 5.8 Hz, H-5'a), 4.10 (1H, dd, *J* = 9.8, 3.3 Hz, H-5'b), 3.37 (2H, d, *J* = 8.9 Hz, H-7a), 3.12 (1H, d, *J* = 6.9 Hz, H-7b); HR(+)ESI-MS *m/z* 458.8876 / 456.8896 / 454.8923 (3.2:6.6:3.4) $[M + H]^+$ (calcd. for $C_{13}H_{13}{}^{79}Br_2O_8{}^+$, 454.8972); Observed adducts and fragments: 916.7744 / 914.7753 / 912.7756 / 910.7828 / 908.7947 (0.8:3.3:5.2:3.5:0.9) $[2M + H]^+$, 475.9127 / 473.9167 / 471.9183 (38:78:39) $[M + NH_4]^+$; MS/MS *m/z* 282.8638 / 280.0382 / 278.8672 (21:100:39) $[C_7H_5Br_2O_2]^+$, 177.0378 (55) $[C_6H_9O_6]^{+,}$ 141.0137 (68) $[C_6H_5O_4]^+$, 95.0105 (62) $[C_5H_3O_2]^+$.

Methylrhodomelol (3-(2,3-dibromo-4,5-dihydroxybenzyl)-3a,6-dihydroxy-3-methoxytetrahydrofuro-[3,2-b]furan-2(3H)-one) (9): orange oil; UV (MeCN, H_2O) λ_{max} 212, 290 nm; ^{1}H NMR (CD_3OD, 600 MHz) δ 6.94 (1H, s, H-6), 4.63 (1H, s, H-6'a), 4.42 (1H, dd, *J* = 5.9, 3.4 Hz, H-6'), 4.28 (1H, dd, *J* = 9.8, 5.7 Hz, H-5'a), 4.14 (1H, dd, *J* = 9.8, 3.3 Hz, H-5'b), 3.60 (3H, s, H-7'), 3.37 – 3.34 (2H, m, H-7); ^{13}C NMR (CD_3OD, 151 MHz) δ 173.26 (C-2'), 145.78 (C-5), 144.90 (C-4), 128.06 (C-1), 118.70 (C-6), 117.92 (C-2), 113.66 (C-3), 110.52 (C-3'a), 88.91 (C-6'a), 83.96 (C-3'), 76.88 (C-5'), 74.79 (C-6'), 54.63 (C-7'), 38.94 (C-7); HR(+)ESI-MS *m/z* 472.9109 / 470.9133 / 468.9168 (8.2:17:8.5) $[M + H]^+$ (calcd. for $C_{14}H_{15}{}^{79}Br_2O_8{}^+$, 468.9128); Observed adducts and fragments: 966.8024 / 964.8024 / 962.8032 / 960.8079 / 958.8172 (6.6:22:32:23:8.3) $[2M + Na]^+$, 961.8146 / 959.8382 / 957.8479 / 955.8487 / 953.8484 (8.6:7.6:8.6:5.6:1.6; isotopic pattern is distorted due to

^{13}C isotopes of the sodium adduct) $[2M + NH_4]^+$, 944.8212 / 942.8191 / 940.8205 / 938.8234 / 936.8241 (1.6:5.4:7:7:5.2:1.5) $[2M + H]^+$, 494.8938 / 492.8971 / 490.9184 (9.1:18:14) $[M + Na]^+$, 489.9388 / 487.9405 / 485.9428 (51:100:50) $[M + NH_4]^+$, 454.9014 / 452.9041 / 450.9050 (2.5:4.8:2.4) $[M – H_2O]^+$, 282.8622 / 280.8640 / 278.8652 (0.9:1.8:0.9) $[C_7H_5Br_2O_2]^+$, 191.0561 (38) $[C_7H_{11}O_6]^+$, 173.0458 (9.0) $[C_7H_9O_5]^+$, 155.0343 (2.8) $[C_7H_7O_4]^+$, 143.0338 (7.9) $[C_6H_7O_4]^+$, 117.0194 (5.3).

L-Phenylalanine (10): orange amorphous solid; $[\alpha]^{20}_D$ -23.7 (*c* 0.2, H_2O); UV (MeCN, H_2O) λ_{max} 200, 260 nm; ^1H NMR (D_2O, 600 MHz) δ 7.46 – 7.41 (2H, m, H-3, H-5), 7.41 – 7.37 (1H, m, H-4), 7.36 – 7.32 (2H, m, H-2, H-6), 4.30 (1H, dd, *J* = 7.8, 5.4 Hz, H-2), 3.36 (1H, dd, *J* = 14.7, 5.6 Hz, H-βa), 3.22 (1H, dd, *J* = 14.7, 7.8 Hz, H-βb); ^{13}C NMR (D_2O, 151 MHz) δ 172.72 (COOH), 135.08 (C-1), 130.37 (C-2, C-6), 130.17 (C-3, C-5), 128.94 (C-4), 55.36 (C-α), 36.66 (C-β); HR(+)ESI-MS *m/z* 166.0893 (100) $[M + H]^+$ (calcd. for $C_9H_{12}NO_2^+$, 166.0893); Observed adducts and fragments: 331.1717 (1.3) $[2M + H]^+$, 120.0832 (89) $[M – COOH]^+$.

L-Tyrosine (11): off white amorphous solid; $[\alpha]^{20}_D$ -31.4 (*c* 0.1, H_2O); UV (MeCN, H_2O) λ_{max} 224, 276 nm; ^1H NMR (D_2O, 600 MHz) δ 7.22 – 7.17 (2H, m, H-2, H-6), 6.91 – 6.87 (2H, m, H-3, H-5), 4.14 (1H, dd, *J* = 7.7, 5.4 Hz, α-H), 3.25 (1H, dd, *J* = 14.7, 5.4 Hz, H-βa), 3.11 (1H, dd, *J* = 14.7, 7.7 Hz,H-βb); ^{13}C NMR (D_2O, 151 MHz) δ 173.47 (COOH), 156.02 (C-4), 131.75 (C-2, C-6), 127.10 (C-1), 116.82 (C-3, C-5), 55.98 (C-α), 36.03 (C-β); HR(+)ESI-MS *m/z* 182.0837 (100) $[M + H]^+$ (calcd. for $C_9H_{12}NO_3^+$, 182.0837); Observed adducts and fragments: 165.0558 (73) $[M – NH_3]^+$, 147.0446 (4.3) $[M – NH_3 – H_2O]^+$, 136.0765 (39), 123.0452 (4.9).

Porphyra-334 (((5S,E)-3-((1-carboxy-2-hydroxypropyl)iminio)-5-hydroxy-5-(hydroxymethyl)-2-methoxycyclohex-1-en-1-yl)glycinate) (17): orange oil; UV (MeCN, H_2O) λ_{max} 336 nm; ^1H NMR (D_2O, 600 MHz) δ 4.30 (1H, ddd, *J* = 6.5, 4.5, 2.9 Hz, H-2'), 4.07 (1H, dd, *J* = 4.6, 2.5 Hz, H-1'), 4.04 (2H, q, *J* = 3.0 Hz, H-9), 3.69 (3H, t, *J* = 2.4 Hz, H-8), 3.57 (2H, t, *J* = 2.6 Hz, H-7), 2.91 (1H, d, *J* = 2.3 Hz, H-6a), 2.84 – 2.80 (1H, m, H-4a), 2.77 (1H, ddt, *J* = 9.6, 3.9, 1.9 Hz, H-4b), 2.75 – 2.72 (1H, m, H-6b), 1.25 (3H, dt, *J* = 6.8, 2.3 Hz, H-3'); ^{13}C NMR (D_2O, 151 MHz) δ 175.40 (C-4'), 174.91 (C-10), 160.63 (C-3), 159.06 (C-1), 125.74 (C-2), 71.15 (C-5), 68.28 (C-2'), 64.50 (C-1'), 59.47 (C-8), 46.74 (C-9), 33.37 (C-6), 32.93 (C-4), 19.49 (C-3'); HR(+)ESI-MS *m/z* 347.1452 (100) $[M + H]^+$ (calcd. for $C_{14}H_{23}N_2O_8^+$, 347.1449).

Aplysiapalythine A (((5S,E)-5-hydroxy-5-(hydroxymethyl)-3-((2-hydroxypropyl)iminio)-2-methoxy-cyclohex-1-en-1-yl)glycinate) (18): yellow oil; UV (MeCN, H_2O) λ_{max} 332 nm; ^1H NMR (D_2O, 600 MHz) δ 4.03 (2H, t, *J* = 1.4 Hz, H-9), 4.02 – 3.99 (1H, m, H-2'), 3.63 (3H, d, *J* = 1.5 Hz, H-8), 3.59 (2H, t, *J* = 1.5 Hz, H-7), 3.47 (1H, d, *J* = 1.4 Hz, H-1'a), 3.45 (1H, d, *J* = 1.4 Hz, H-1'b), 2.89 (2H, s, H-6), 2.77 (1H, d, *J* = 1.3 Hz, H-4a), 2.75 (1H, d, *J* = 1.3 Hz, H-4b), 1.23 (3H, dd, *J* = 6.4, 1.5 Hz, H-3'); ^{13}C NMR (D_2O, 151 MHz) δ 177.60 (C-10), 162.72 (C-1), 162.17 (C-3), 127.87 (C-2), 73.62 (C-5), 70.02 (C-7), 69.22 (C-2'), 61.58 (C-8), 51.45 (C-1'), 49.06 (C-9), 35.63 (C-4), 35.32 (C-6), 21.91 (C-3'); HR(+)ESI-MS *m/z* 303.1558 (100) $[M + H]^+$ (calcd. for $C_{13}H_{23}N_2O_6^+$, 303.1551); Observed adducts and fragments: 341.1116 (4.6) $[M + K]^+$, 325.1381 (5.6) $[M + Na]^+$, 275.1614 (8.2), 245.1498 (6.3) $[M – CH_2CO_2]^+$.

Palythine ((S)-(5-hydroxy-5-(hydroxymethyl)-3-imino-2-methoxycyclohex-1-en-1-yl)glycine) (19): orange oil; UV (MeCN, H_2O) λ_{max} 320 nm; ^1H NMR (D_2O, 600 MHz) δ 4.03 (2H, dt, *J* = 3.3, 1.6 Hz, H-9), 3.64 (3H, dd, *J* = 3.8, 1.9 Hz, H-8), 3.56 (1H, dd, *J* = 4.2, 2.0 Hz, H-7), 2.95 (1H, dd, *J* = 17.2, 2.9 Hz, H-6a), 2.83 (1H, dd, *J* = 17.6, 2.7 Hz, H-4a), 2.78 – 2.72 (1H, m, H-4b), 2.68 (1H, ddd, *J* = 17.0, 3.6, 1.8 Hz, H-6b);^{13}C NMR (D_2O, 151 MHz) δ 174.90 (C-10)*, 161.90 (C-1), 160.29 (C-3), 124.71 (C-2), 71.39 (C-5), 67.49 (C-7), 59.04 (C-8), 46.76 (C-9), 35.87 (C-6), 33.49 (C-4); ^{13}C NMR (D_2O, 151 MHz) δ 174.90 (C-9; assigned via HMBC), 161.90 (C-3), 160.29 (C-1), 124.71 (C-2), 71.39 (C-5), 67.49 (C-7), 59.04 (C-10), 46.76 (C-8), 35.87 (C-4), 33.49 (C-6); HR(+)ESI-MS *m/z* 245.1124 (100) $[M + H]^+$ (calcd. for $C_{10}H_{17}N_2O_5^+$, 245.1132); Observed adducts and fragments: 267.0938 (12) $[M + Na]^+$, 217.1166 (5.5), 187.1062 (11).

(4Z,7Z,10Z,13Z)-Hexadecatetraenoic acid 3′-β-D-galactopyranosyl-1-glycerol ester (2-hydroxy-3-(((1R,2R,3S,4S,5R)-2,3,4-trihydroxy-5-(hydroxymethyl)cyclohexyl)oxy)propyl (4Z,7Z,10Z,13Z)-hexadeca-4,7,10,13-tetraenoate) (20): dark brown amorphous solid; UV (MeCN, H_2O) λ_{max} 230 nm; for NMR data see Table S1.; HR(+)ESI-MS *m/z* 485.2745 (32) $[M + H]^+$ (calcd. for $C_{25}H_{41}O_9^+$, 485.2745); Observed adducts and fragments: 991.5261 (13) $[2M + Na]^+$, 986.5717 (29) $[2M + NH_4]^+$, 969.5452 (8) $[2M + H]^+$, 507.9814 (10) $[M + Na]^+$, 502.3021 (100) $[M + NH_4]^+$, 467.2643 (36) $[M – H_2O]^+$, 323.2220 (69) $[M – Gal]^+$, 305.2110 (6.7) $[M – Gal – H_2O]^+$, 231.1744 (27) (fatty acid oxocarbenium ion $[C_{16}H_{23}O]^+$), 213.1614 (4.3) (fatty acid carbenium ion $[C_{16}H_{21}]^+$).

(6Z,9Z,12Z,15Z)-Stearidonic acid 3′-β-D-galactopyranosyl-1-glycerol ester (2-hydroxy-3-(((3R,4S,5R,6R)-3,4,5-trihydroxy-6-(hydroxymethyl)tetrahydro-2H-pyran-2-yl)oxy)propyl (5Z,8Z,11Z,14Z)-octadeca-5,8,11,14-tetraenoate) (21): greenish brown amorphous solid; UV (MeCN, H_2O) λ_{max} 212 nm; for NMR data see Table S1.; HR(+)ESI-MS *m/z* 513.3077 (37) $[M + H]^+$ (calcd. for $C_{27}H_{45}O_9^+$, 513.3058); Observed adducts and fragments: 1047.5954 (9.5) $[2M + Na]^+$, 1042.6393 (30) $[2M + NH_4]^+$, 1025.6113 (18) $[2M + H]^+$, 535.2895 (4.8) $[M + Na]^+$, 530.3355 (75) $[M + NH_4]^+$, 495.2984 (73) $[M – H_2O]^+$, 351.2549 (100) $[M – Gal]^+$, 259.2069 (19) (fatty acid oxocarbenium ion, $[C_{18}H_{27}O]^+$), 241.1952 (5.2) (fatty acid carbenium ion $[C_{18}H_{25}]^+$).

(5Z,8Z,11Z,14Z,17Z)-Eicosapentaenoic acid 3′-β-D-galactopyranosyl-1-glycerol ester (2-hydroxy-3-(((3R,4S,5R,6R)-3,4,5-trihydroxy-6-(hydroxymethyl)tetrahydro-2H-pyran-2-yl)oxy)propyl (5Z,8Z,11Z,14Z,17Z)-icosa-5,8,11,14,17-pentaenoate) (22): yellowish brown amorphous solid; $[\alpha]^{20}_D$ -5.1 (*c* 0.7, MeOH); UV (MeCN, H_2O) λ_{max} 212 nm; for NMR data see Table S1.; HR(+)ESI-MS *m/z* 539.3242 (44) $[M + H]^+$ (calcd. for $C_{29}H_{47}O_9^+$, 539.3215); Observed adducts and fragments: 1094.6733 (16) $[2M + NH_4]^+$, 1077.6446 (24) $[2M + H]^+$, 556.3501 (51) $[M + NH_4]^+$, 521.3132 (94) $[M – H_2O]^+$, 377.2687 (100) $[M – Gal]^+$, 285.2231 (21) (fatty acid oxocarbenium ion, $[C_{20}H_{29}O]^+$), 267.2105 (6.6) (fatty acid carbenium ion $[C_{20}H_{27}]^+$).

Table S1. ^{1}H (600 MHz)- and ^{13}C-NMR (151 MHz) data of **20 – 23**.

	**20** in CD_3OD		**21** in CD_3OD		**22** in $CDCl_3$		**23** in acetone-d_6	
**Pos.**	**^{13}C**	**^{1}H**	**^{13}C**	**^{1}H**	**^{13}C**	**^{1}H**	**^{13}C**	**^{1}H**
1	174.74 (COOR)	-	175.32 (COOR)	-	174.40 (COOR)	-	175.25 (COOR)	-
2	34.92 (CH_2)	2.40 (4H, m)[a]	34.82(CH_2)	2.38 (t, 7.5)	33.70 (CH_2)	2.35 (t, 7.8)	34.34 (CH_2)	2.39 (t, 7.4)
3	23.74 (CH_2)	[a]	25.62(CH_2)	1.65 (dt, 15.1, 7.3)	24.88 (CH_2)	1.66 (p, 7.5)	25.90 (CH_2)	1.70 (p, 7.4)
4	*	*	30.16 (CH_2)	1.42 (p, 7.6)	25.75 (CH_2)	2.12 – 2.07 (m)	27.56 (CH_2)	2.15 (m)
5	*	*	27.88 (CH_2)	2.10 (4H, m)[b]	*	*	*	*
6	**	**	130.66 (CH)	*	*	*	*	*
7	*	*	*	*	**	**	**	**
8	*	*	**	**	*	*	*	*
9	**	**	*	*	*	*	*	*
10	*	*	*	*	**	**	**	**
11	*	*	**	**	*	*	*	*
12	**	**	*	*	*	*	*	*
13	*	*	*	*	**	**	**	**
14	*	*	**	**	*	*	*	*
15	21.49 (CH_2)	2.09 (p, 7.4)	*	*	*	*	*	*
16	14.20 (CH_3)	0.98 (t, 7.6)	132.80 (CH)	*	**	**	**	**
17			21.50 (CH_2)	[b]	*	*	*	*
18			14.66 (CH_3)	0.99 (t, 7.5)	132.15 (CH)	*	132.82 (CH)	*
19					20.70 (CH_2)	2.05 (qd, 7.4, 1.7)	21.51 (CH_2)	2.10 (m)
20					14.45 (CH_3)	0.95 (t, 7.6)	14.67 (CH_3)	0.99 (m)
1′	66.68 (CH_2)	4.16 (t, 5.9)	66.59 (CH_2)	4.16 (m)	65.36 (CH_2)	4.10 (d, 6.7)	66.60 (CH_2)	4.16 (m)
2′	69.62 (CH)	4.01 – 3.96 (m)	69.63 (CH)	4.00 (m)	68.60 (CH)	4.06 – 4.02 (m)	69.67 (CH)	4.00 (m)
3′	71.88 (CH_2)	3.66 (dd, 10.6, 4.5)<br>3.92 (dd, 10.6, 5.1)	71.88 (CH_2)	3.93 (dd, 10.6, 5.1)<br>3.67 (dd, 10.4, 4.5)	71.37 (CH_2)	3.89 – 3.84 (m)<br>3.71 (dd, 11.0, 2.9)	72.12 (CH_2)	3.86 (s)<br>3.66 (m)
1′′	105.33 (CH)	4.23 (d, 7.6)	105.33 (CH)	4.24 (d, 7.6)	103.54 (CH)	4.31 (d, 7.6)	105.33 (CH)	4.26 (d, 7.5)
2′′	72.57 (CH)	3.53 (d, 7.6)	72.56 (CH)	3.55 (m)	71.20 (CH)	3.56 (s)	72.53 (CH)	3.55 (dd, 9.8, 7.5)
3′′	74.84 (CH)	3.48 (d, 3.4)	74.84 (CH)	3.48 (dd, 9.7, 3.4)	73.22 (CH)	3.62 (dd, 10.0, 3.2)	74.67 (CH)	3.51 (dd, 9.7, 3.4)

4′′	70.27 (CH)	3.82 (d, 2.3)	70.27 (CH)	3.83 (d, 2.3)	68.76 (CH)	3.97 – 3.94 (m)	70.10 (CH)	3.88 (d, 5.4)
5′′	76.78 (CH)	3.51 (dt, 7.6, 2.0)	76.78 (CH)	3.53 (m)	74.86 (CH)	3.58 (dd, 7.3, 2.4)	74.57(CH)	3.77 (d, 8.1)
6′′	62.48 (CH_2)	3.73 (d, 5.3)<br>3.75 (d, 7.0)	62.47 (CH_2)	3.77 (dd, 11.3, 6.9)<br>3.73 (dd, 11.3, 5.3)	60.97 (CH_2)	3.74 (d, 6.6)	67.78 (CH_2)	3.91 (m)<br>3.70 (d, 3.7)
1′′′							100.56	4.86 (d, 3.8)[c]
2′′′							70.24	3.79 (dd, 10.1, 3.7)
3′′′							71.47	3.75 (d, 6.9)
4′′′							71.06	3.90 (m)
5′′′							72.57	3.86 (d, 5.3)
6′′′							62.75	3.73 (m)
*<br>Olefinic groups (CH)	128.19<br>128.91<br>129.06<br>129.08<br>129.24<br>129.48<br>130.31<br>132.81<br>(C14)	5.28 – 5.43 (m)	129.43<br>129.30<br>129.23<br>129.03<br>128.96<br>128.20	5.37 (8H, m)	129.05<br>129.03<br>128.72<br>128.46<br>128.42<br>128.29<br>128.19<br>127.99<br>127.16	5.40 – 5.26 (10H, m)	130.04<br>129.91<br>129.47<br>129.25<br>129.20<br>129.13<br>129.11<br>128.94<br>128.20	5.44 – 5.29 (10H, m)
**<br>Methylene groups between the olefinic groups (CH_2)	26.42<br>26.49<br>26.51	2.85 – 2.80 (2H, m)<br>2.88 – 2.84 (4H, m)	26.57<br>26.53<br>26.42	2.83 (6H, m)	26.69 (2C)<br>25.75<br>25.68	2.78 (4H, t, 7.0)<br>2.84 – 2.80 (4H, m)	26.57 (2C)<br>26.55<br>26.44	2.86<br>(8H, dt, 16.3, 5.3)

[a] H-2 and H-3 are overlapping (also reported by [68])

[b] H-5 and H-17 appear as one multiplet

[c] Determined in acetone-d_6

NMR spectra of new compounds

Figure S1. 1D and 2D-NMR spectra of **7**.

(a) ^{1}H-NMR spectrum of **7** (1:1 (*v*/*v*) methanol-d_4 / water-d_2, 289 K, 600 MHz).

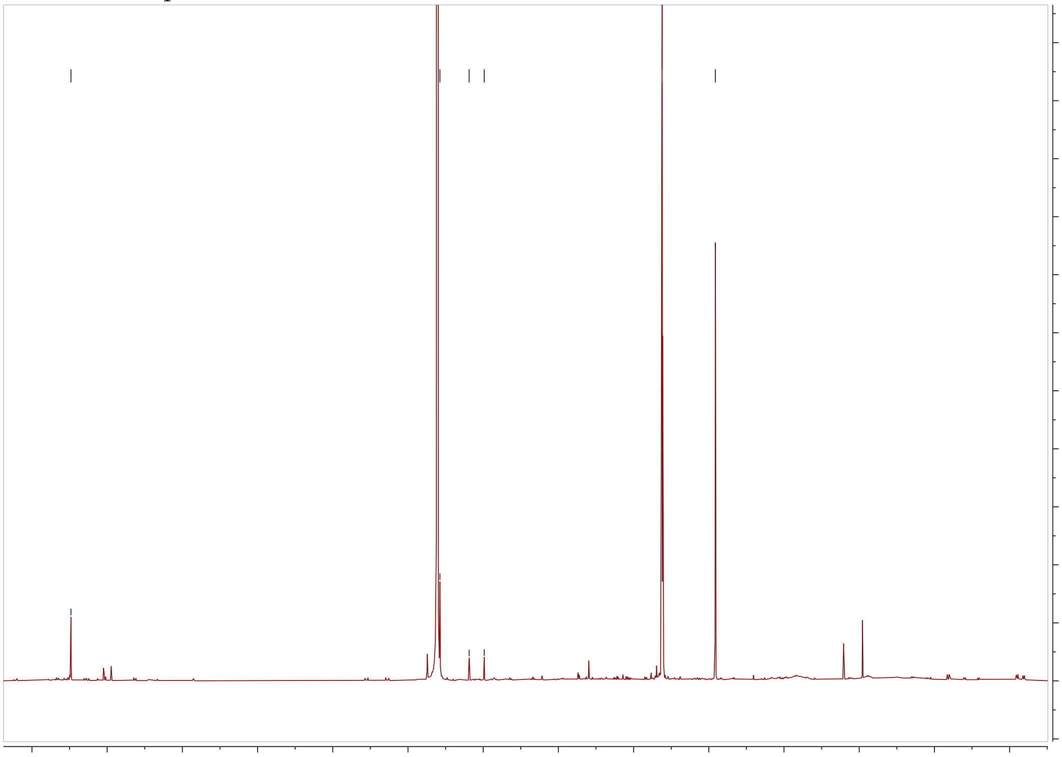

(b) ^{13}C-NMR spectrum of **7** (1:1 (*v*/*v*) methanol-d_4 / water-d_2, 289 K, 600 MHz).

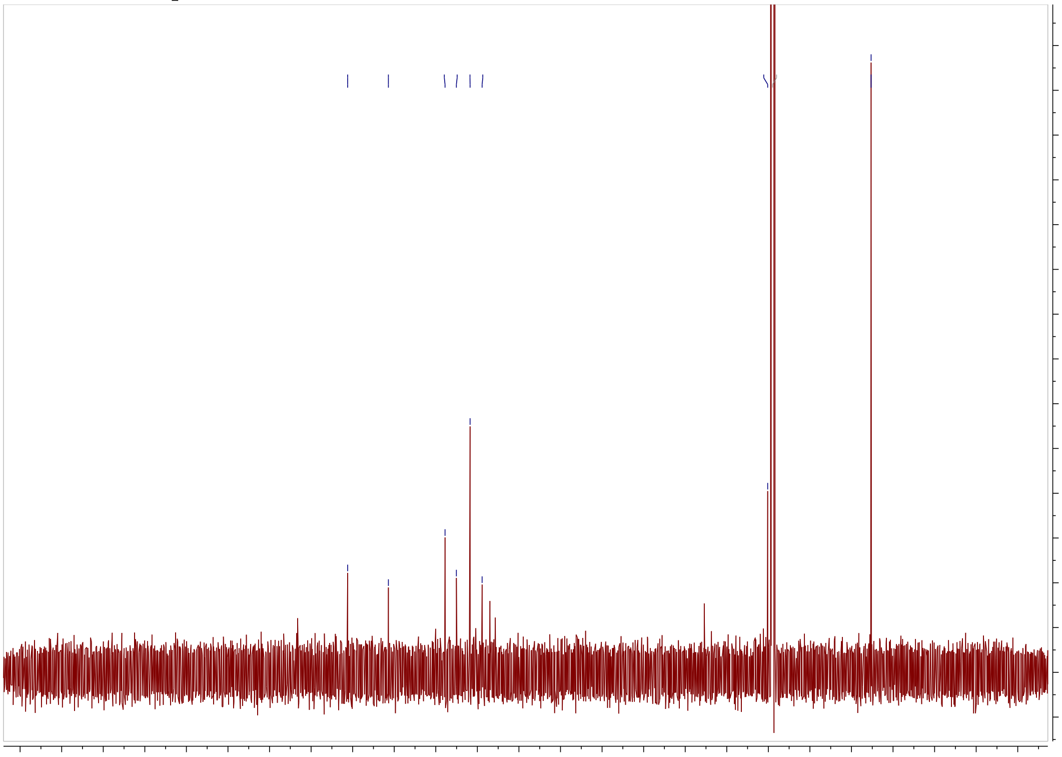

(c) HSQC spectrum of **7** (1:1 (*v*/*v*) methanol-*d*₄ / water-*d*₂, 289 K, 600 MHz).

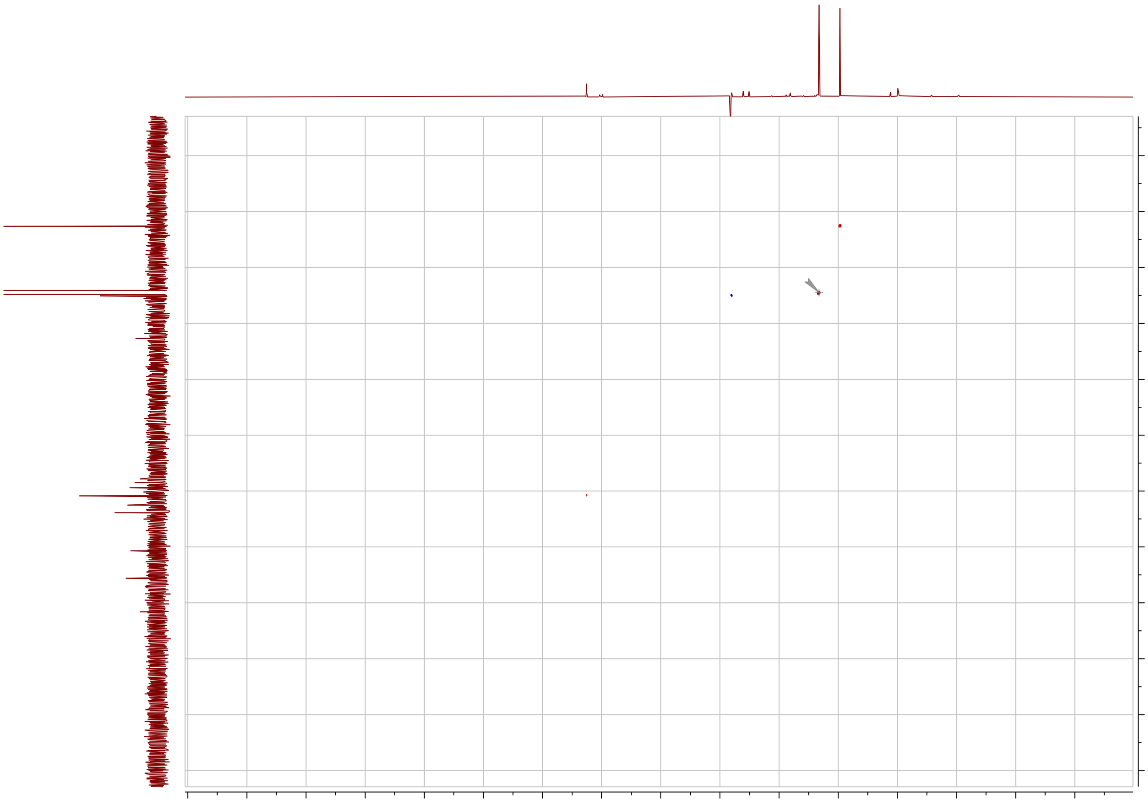

(d) HMBC spectrum of **7** (1:1 (*v*/*v*) methanol-*d*₄ / water-*d*₂, 289 K, 600 MHz).

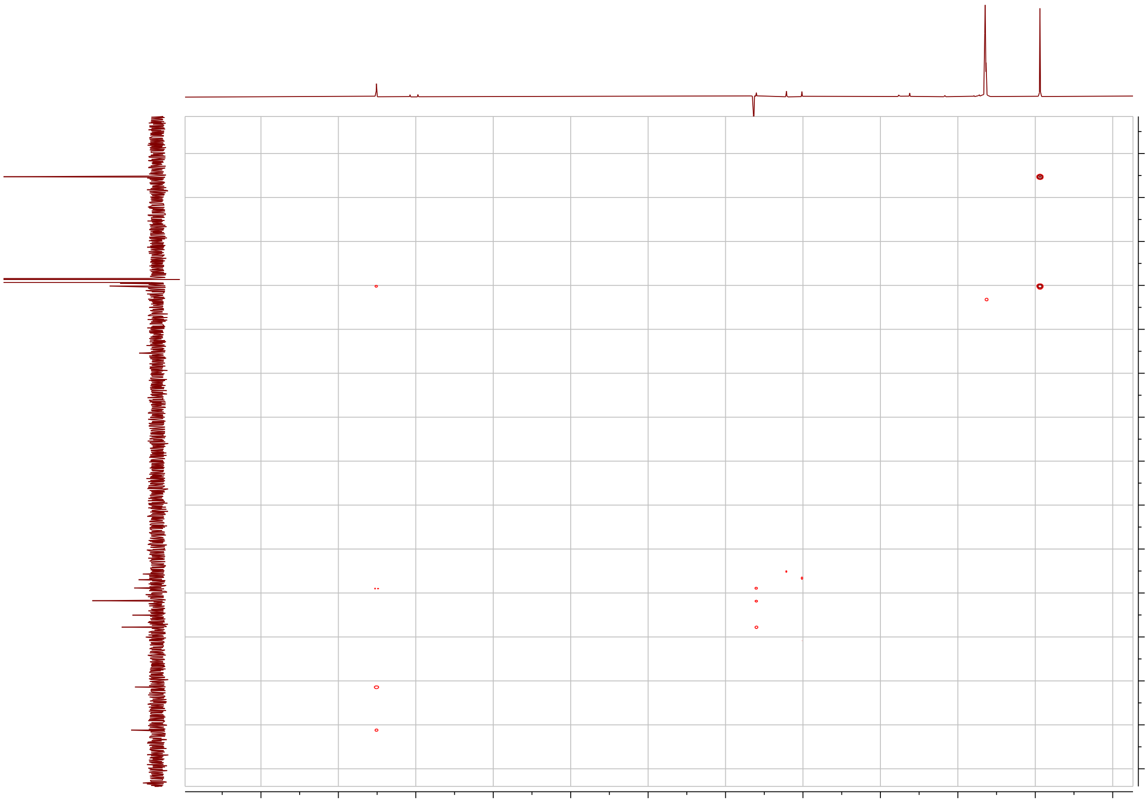

(e) COSY spectrum of **7** (1:1 (*v*/*v*) methanol-d_4 / water-d_2, 289 K, 600 MHz).

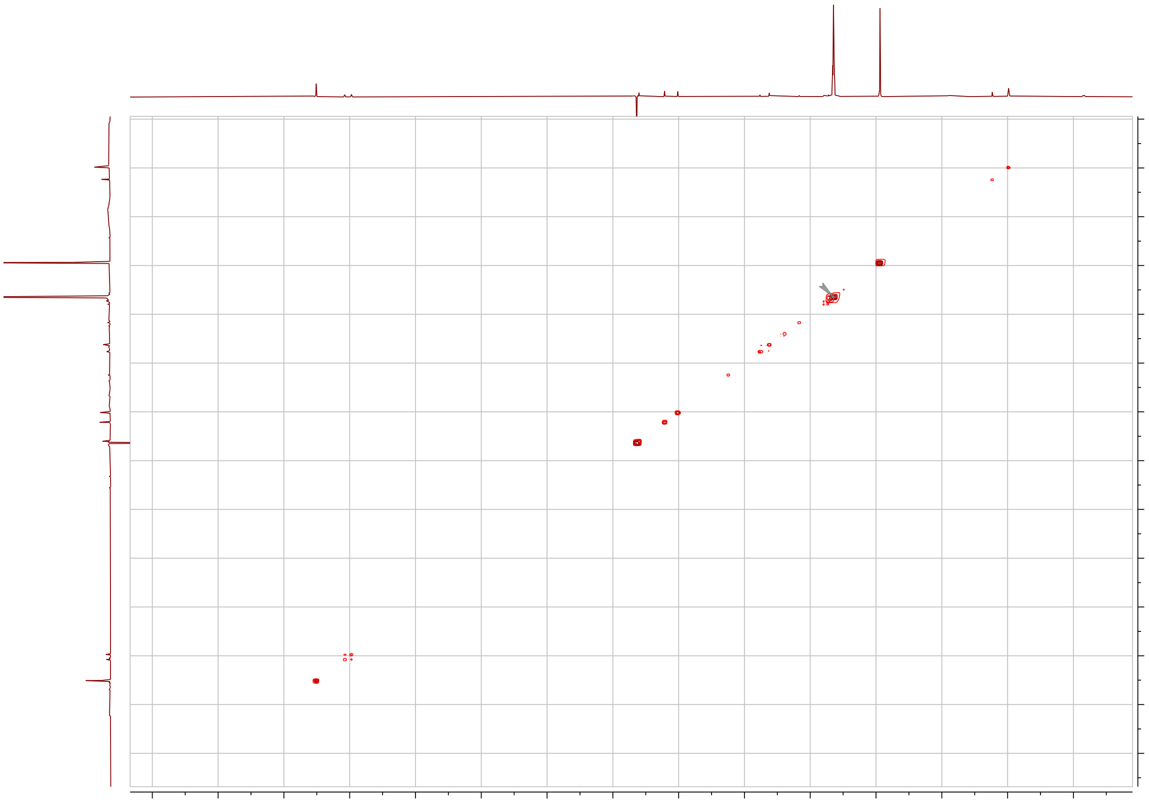

Figure S2. 1D and 2D-NMR spectra of **12.**

(a) ^{1}H-NMR spectrum of **12** (water-d_2, 289 K, 600 MHz).

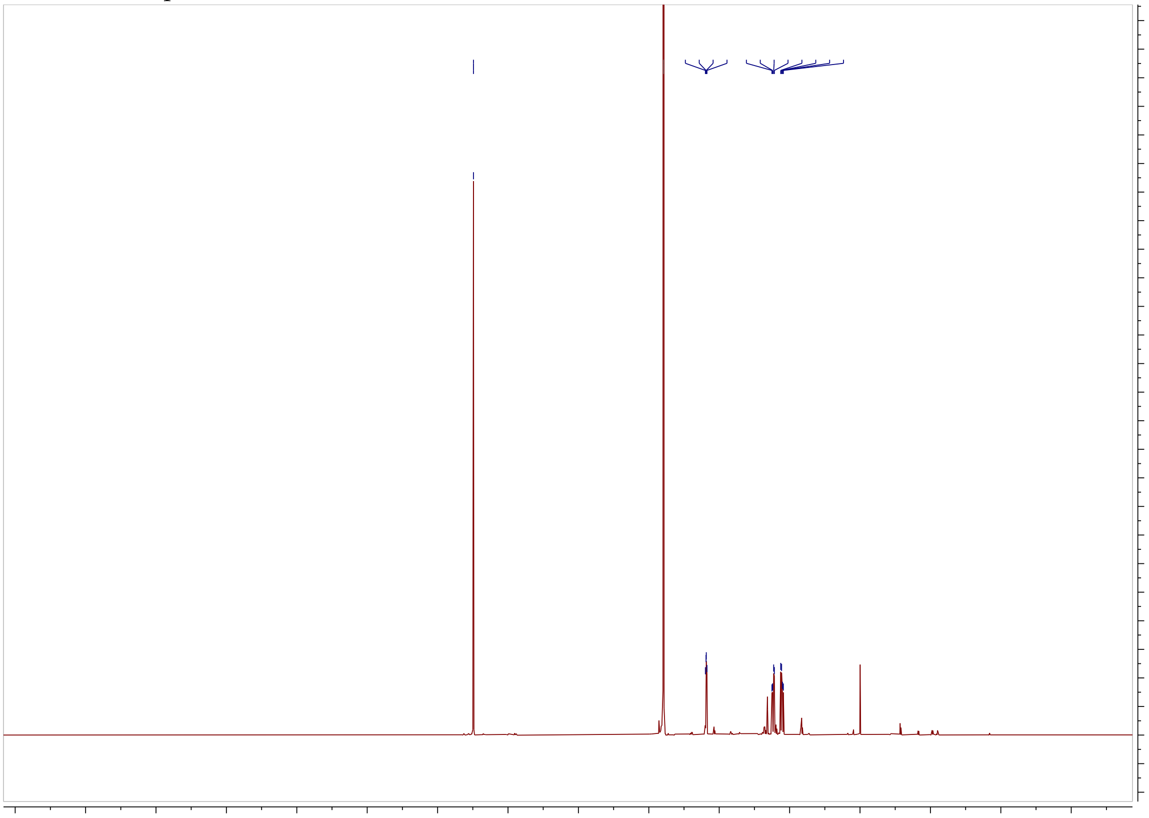

(b) ^{13}C-NMR spectrum of **12** (water-d_2, 289 K, 151 MHz).

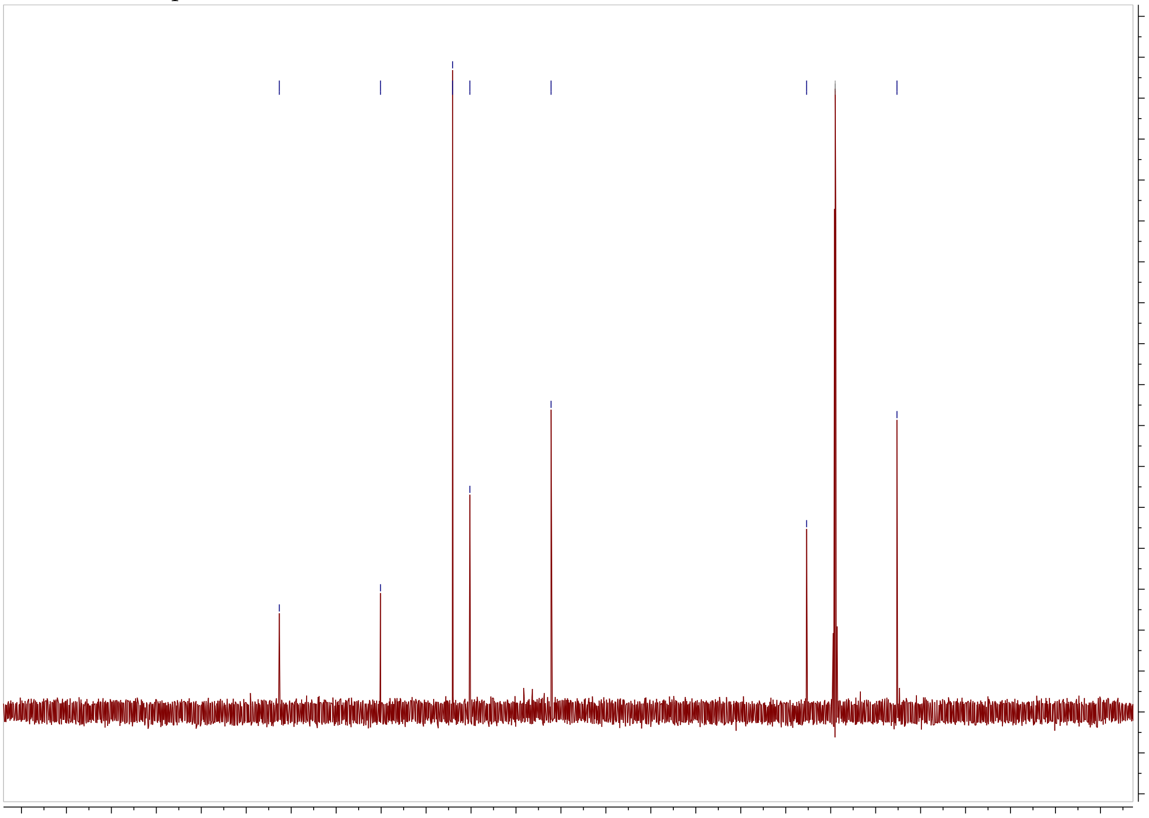

(c) HSQC spectrum of **12** (water-d_2, 289 K, 600 MHz).

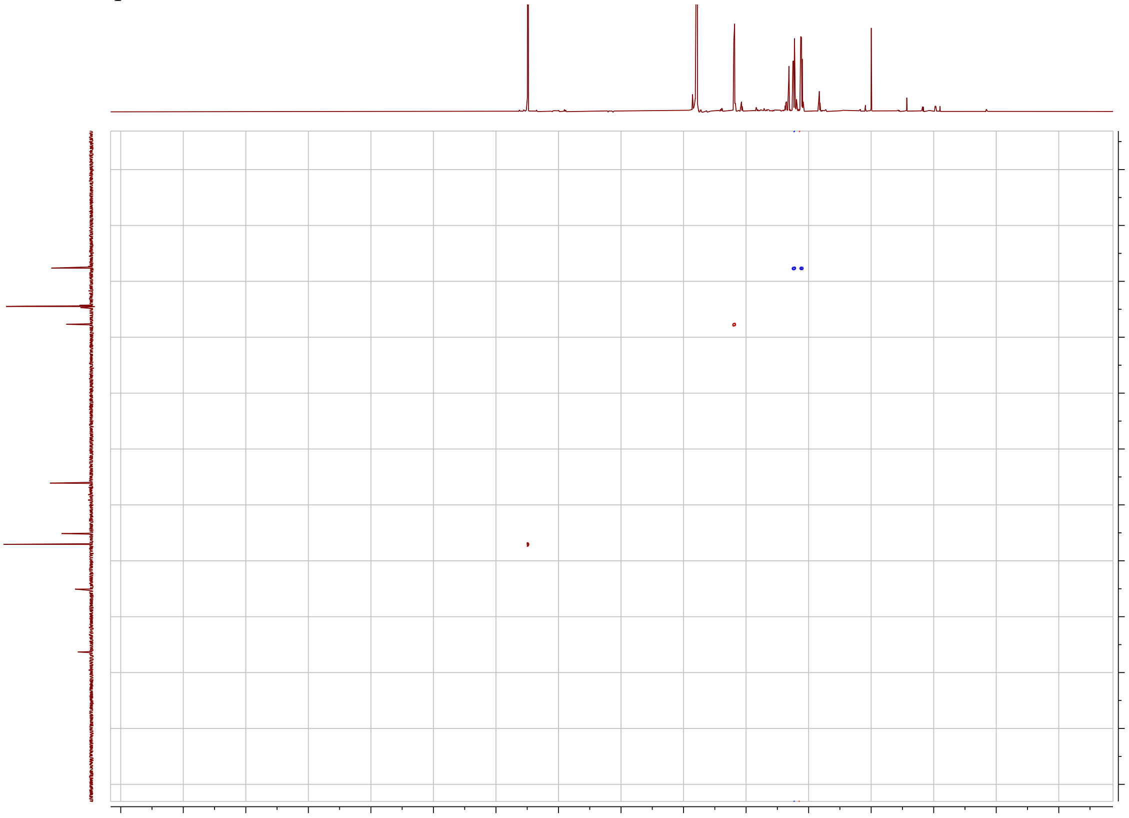

(d) HMBC spectrum of **12** (water-*d*$_2$, 289 K, 600 MHz).

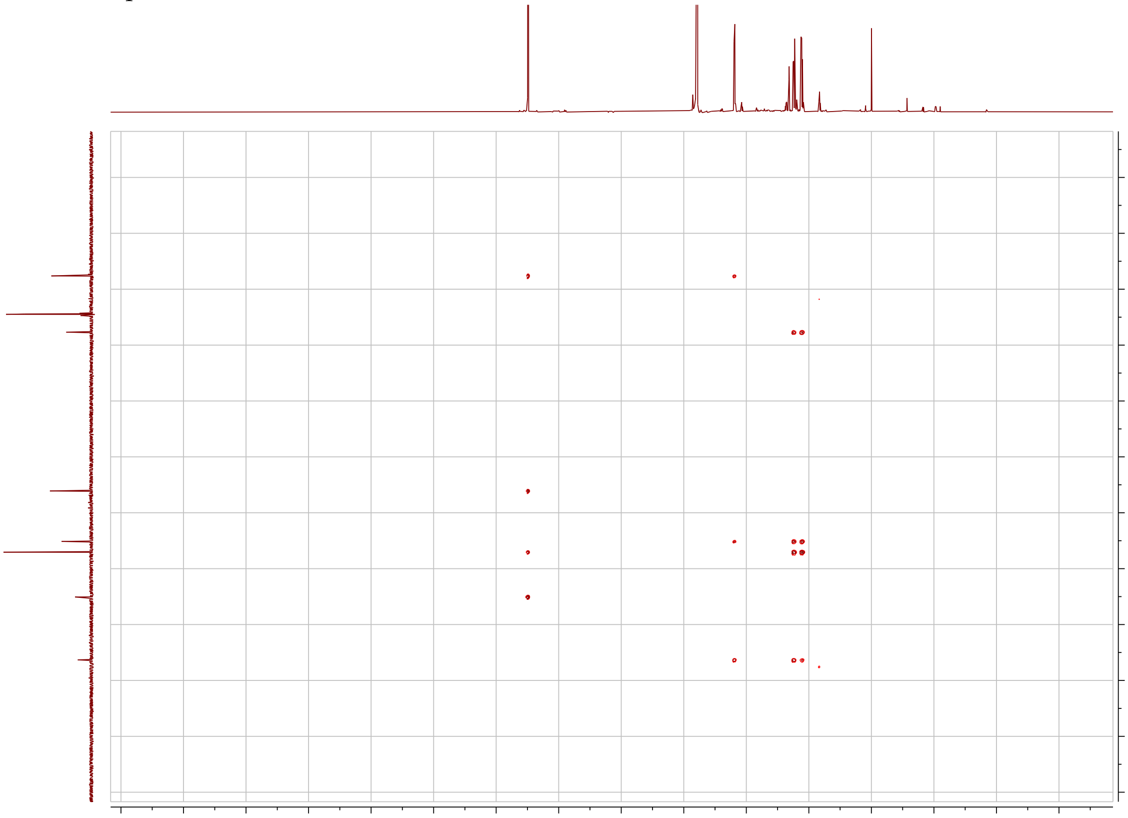

(e) COSY spectrum of **12** (water-*d*$_2$, 289 K, 600 MHz).

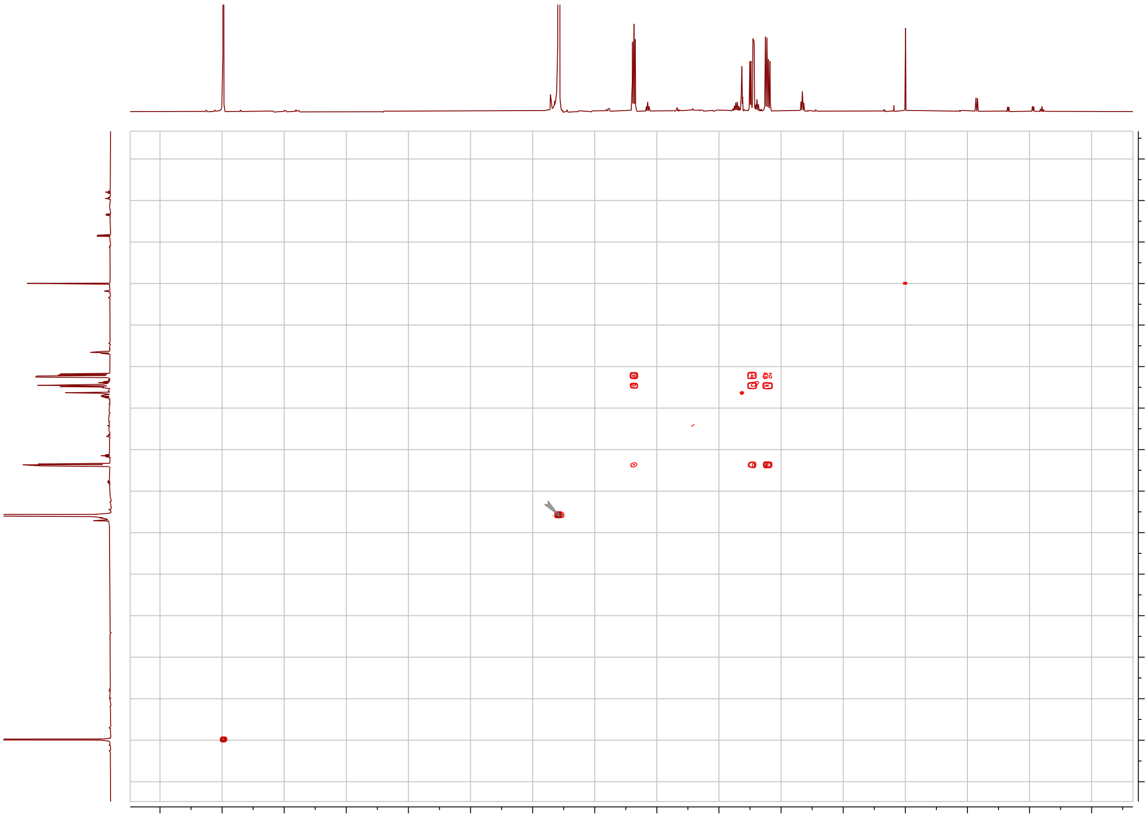

Figure S3. 1D and 2D-NMR spectra of **13.**

(a) ^{1}H-NMR spectrum of **13** (water-d_2, 289 K, 600 MHz).

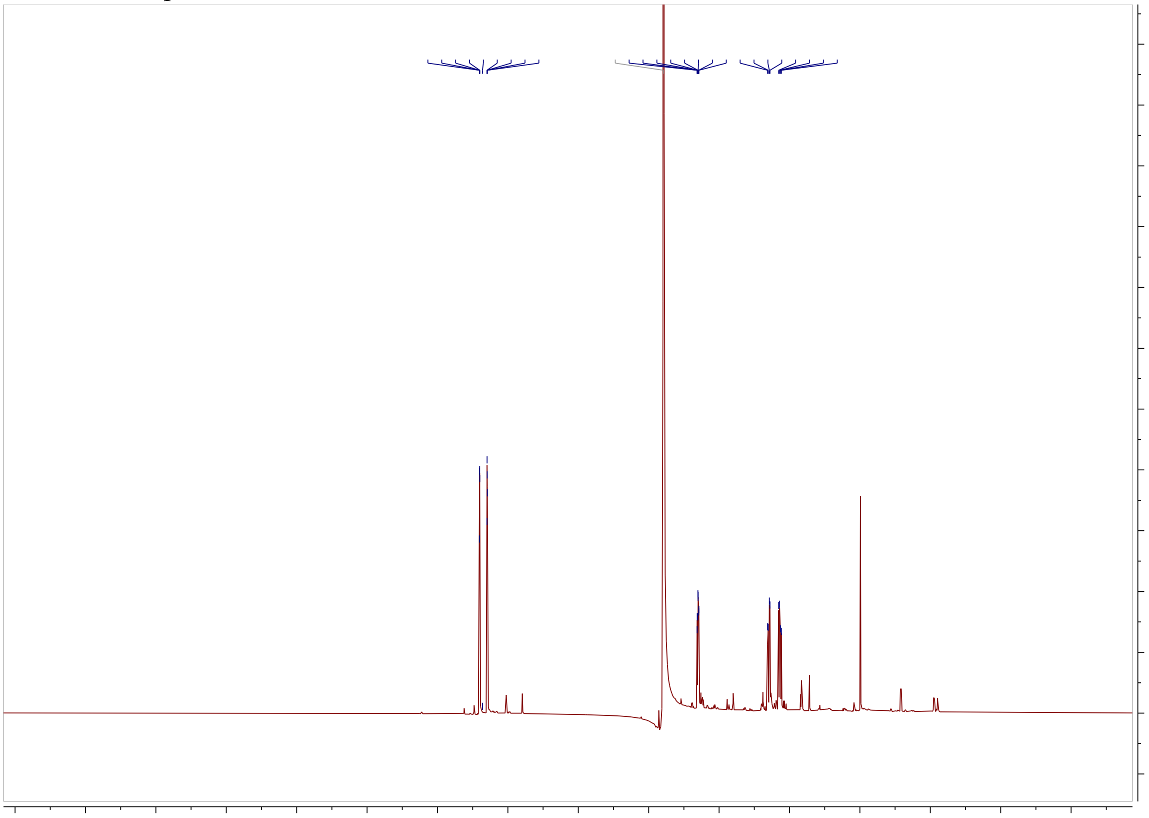

(b) ^{13}C-NMR spectrum of **13** (water-d_2, 289 K, 151 MHz).

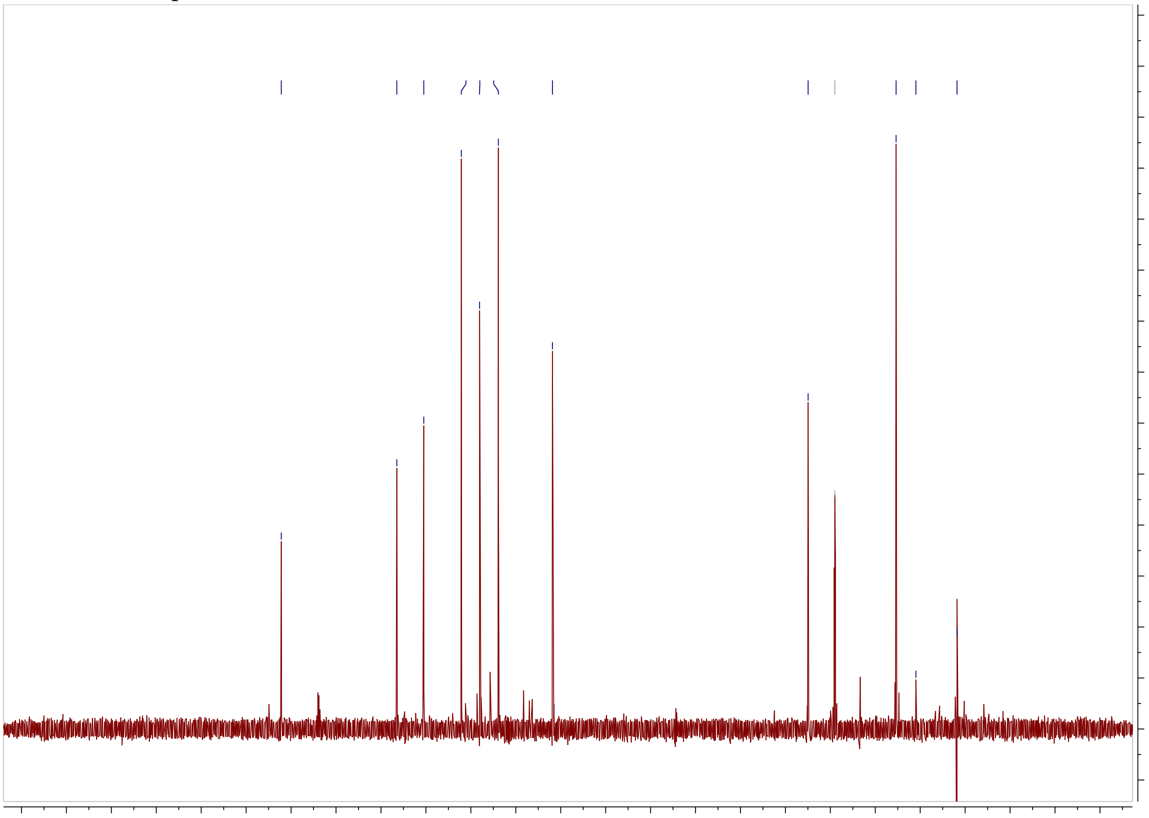

(c) HSQC spectrum of **13** (water-*d*$_2$, 289 K, 600 MHz).

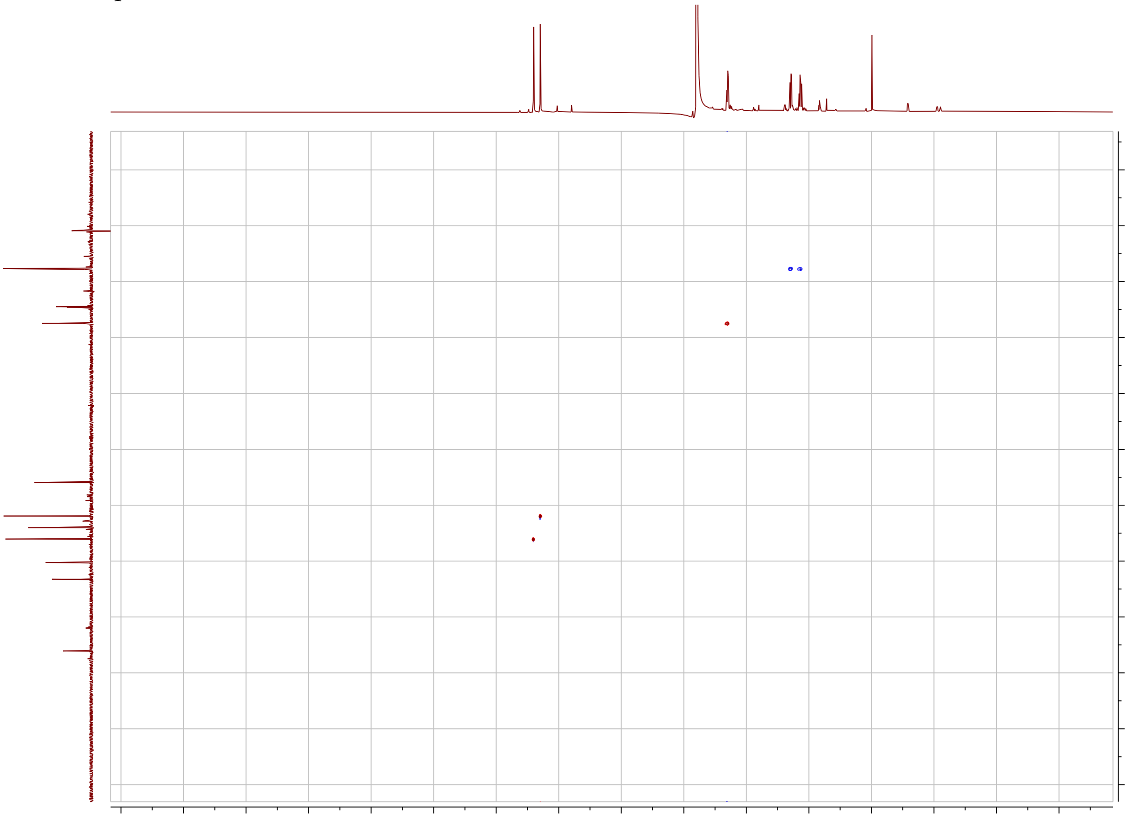

(d) HMBC spectrum of **13** (water-*d*$_2$, 289 K, 600 MHz).

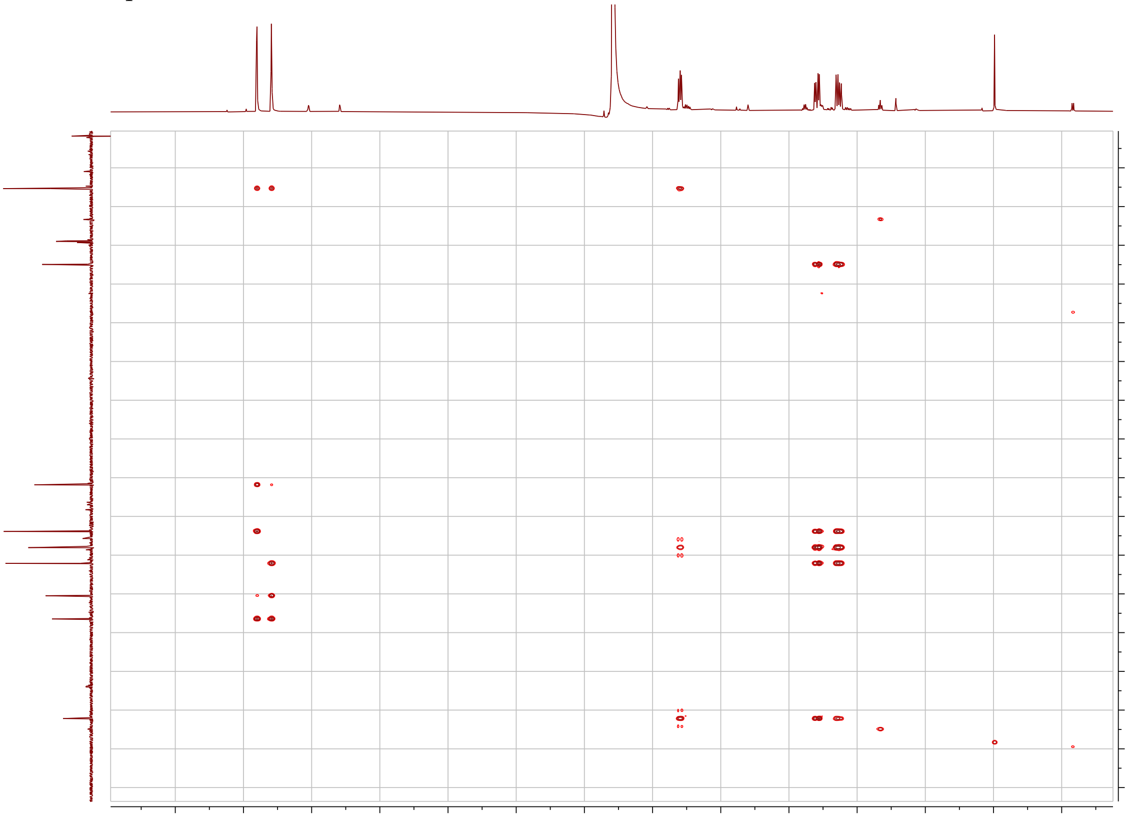

(e) COSY spectrum of **13** (water-*d*$_2$, 289 K, 600 MHz).

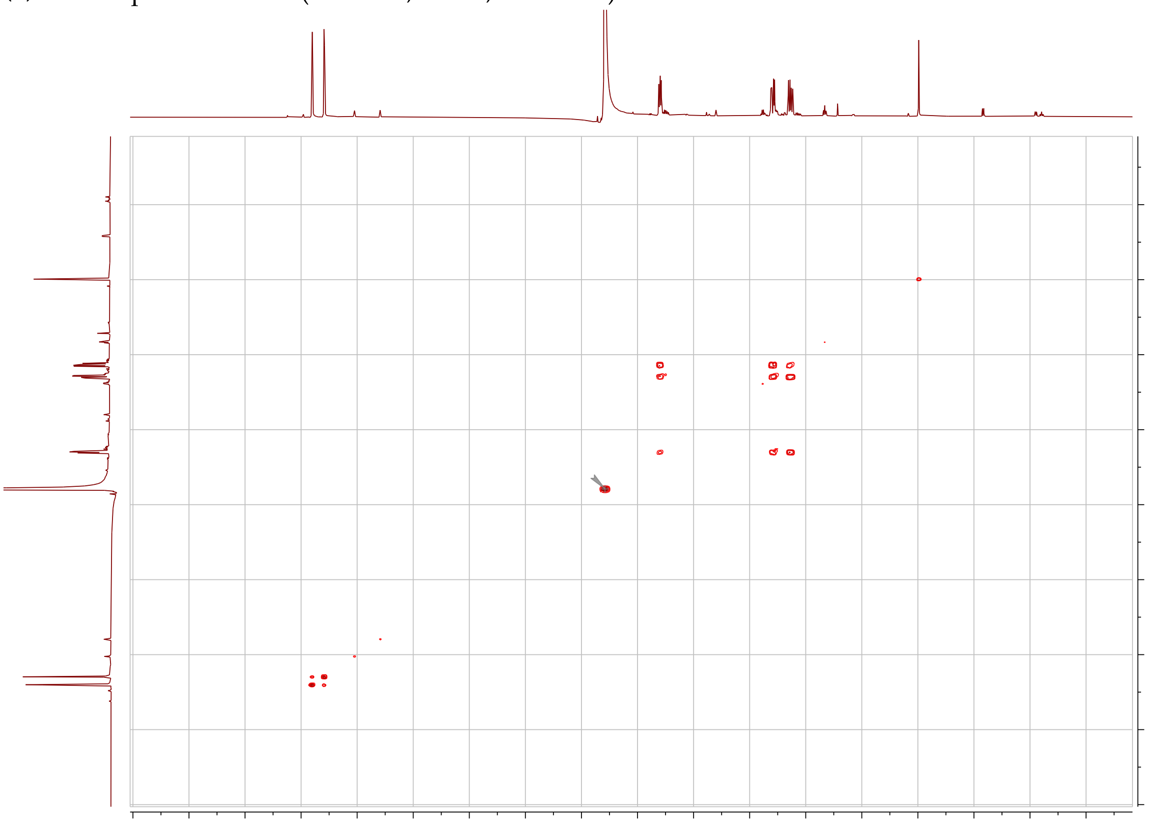

Figure S4. 1D and 2D-NMR spectra of **14**.

(a) ^{1}H-NMR spectrum of **14** (methanol-*d*$_4$, 289 K, 600 MHz).

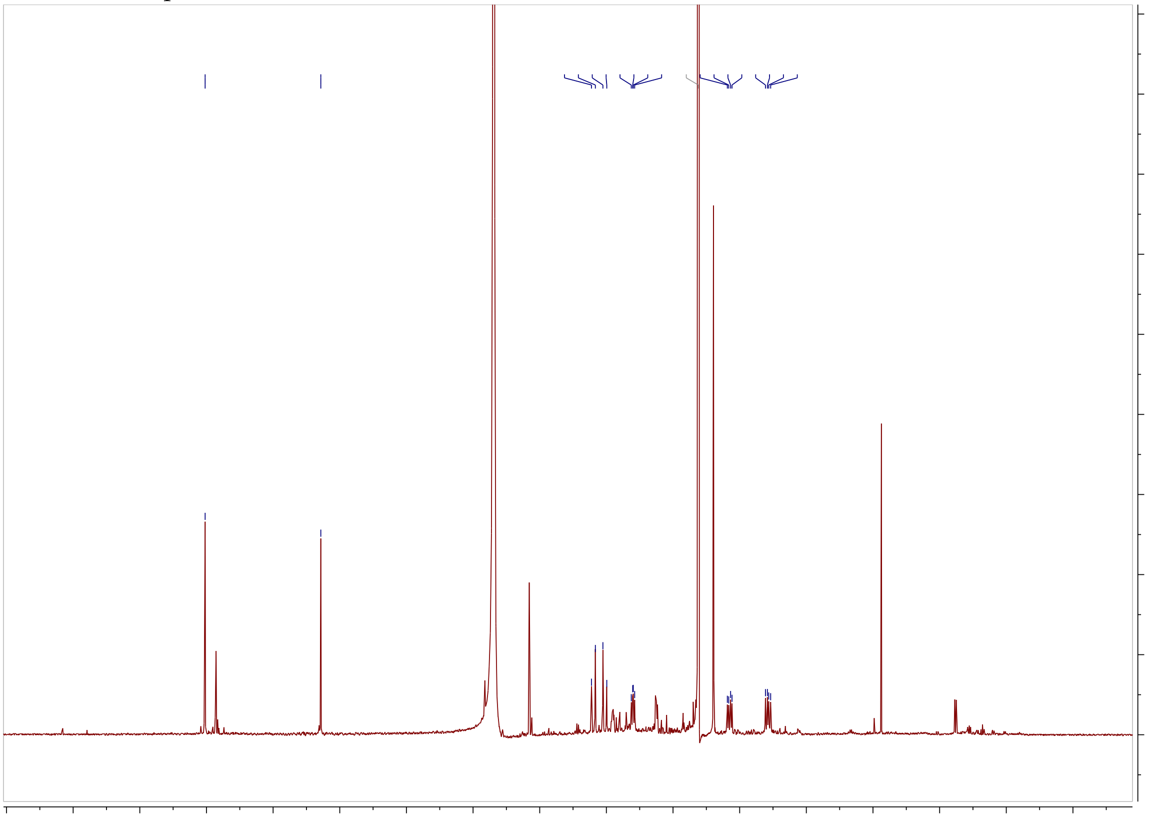

(b) ^{13}C-NMR spectrum of **14** (methanol-*d*$_4$, 289 K, 151 MHz).

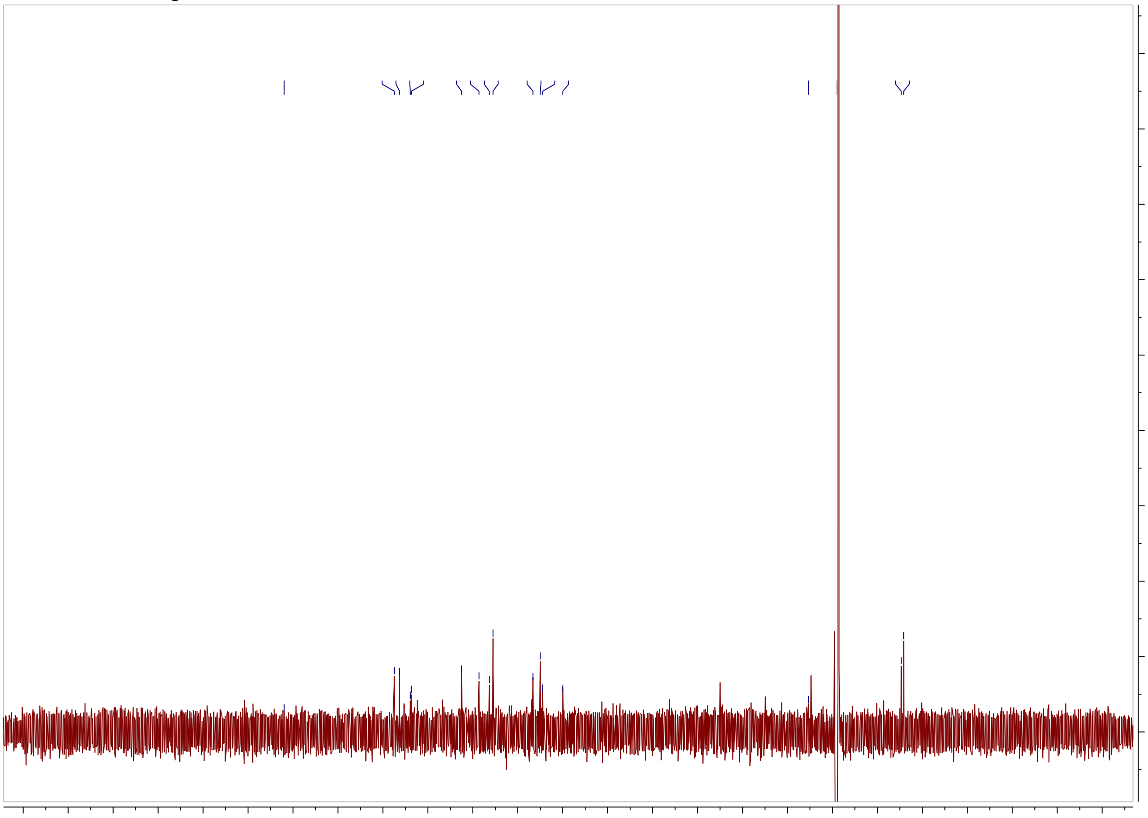

(c) HSQC spectrum of **14** (methanol-*d*$_4$, 289 K, 600 MHz).

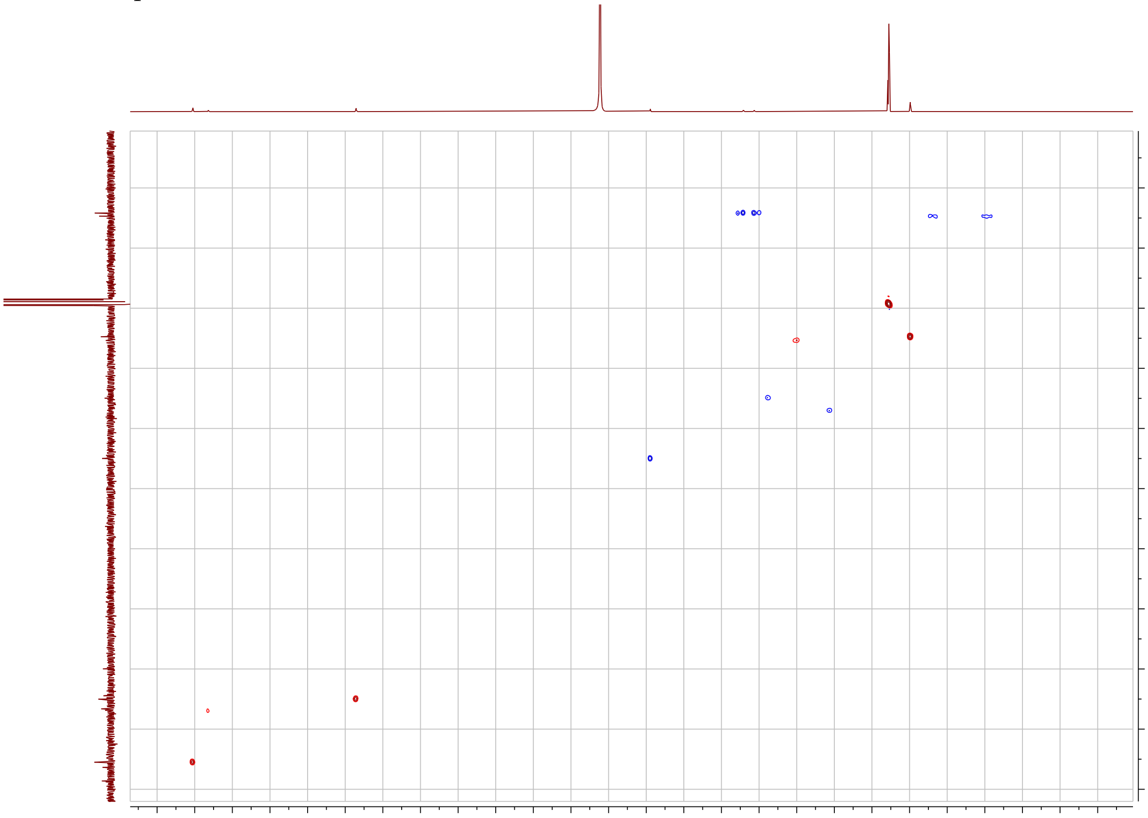

(d) HMBC spectrum of **14** (methanol-d_4, 289 K, 600 MHz).

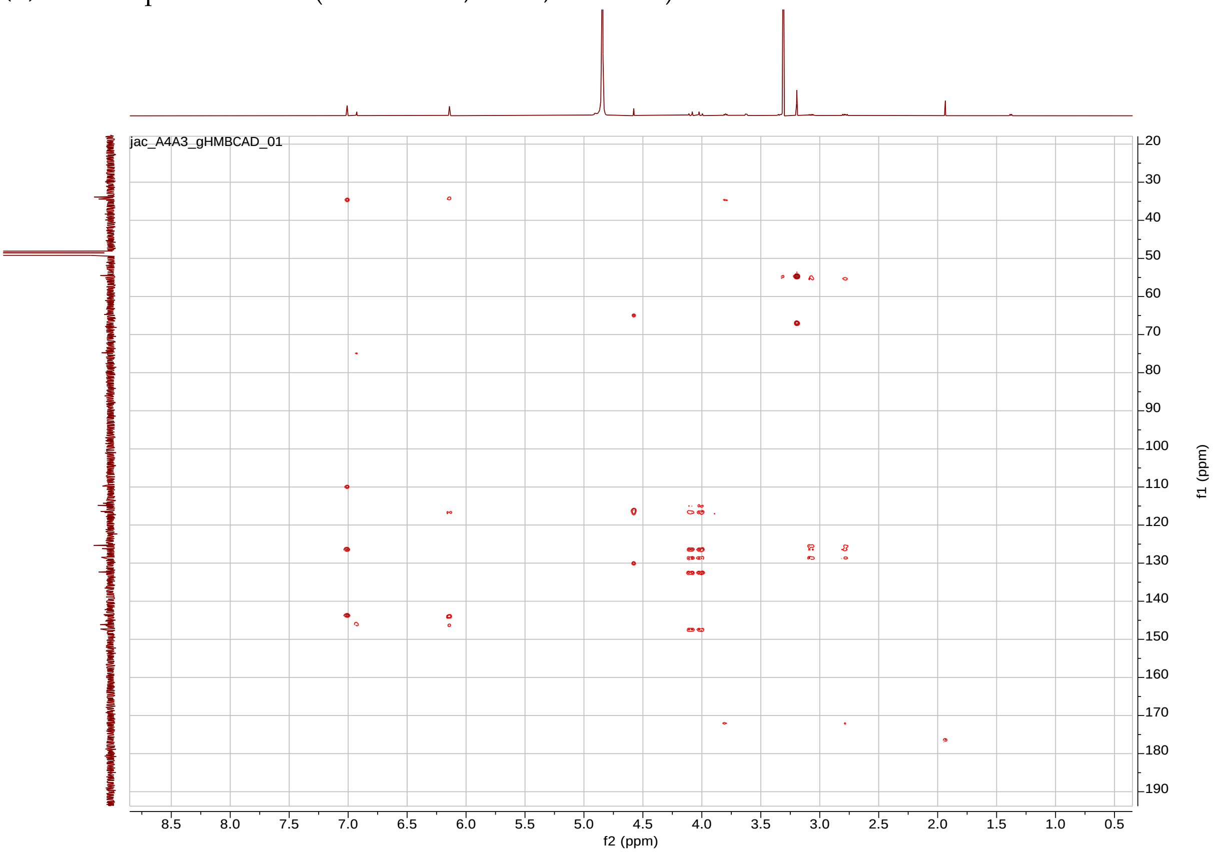

(e) COSY spectrum of **14** (methanol-d_4, 289 K, 600 MHz).

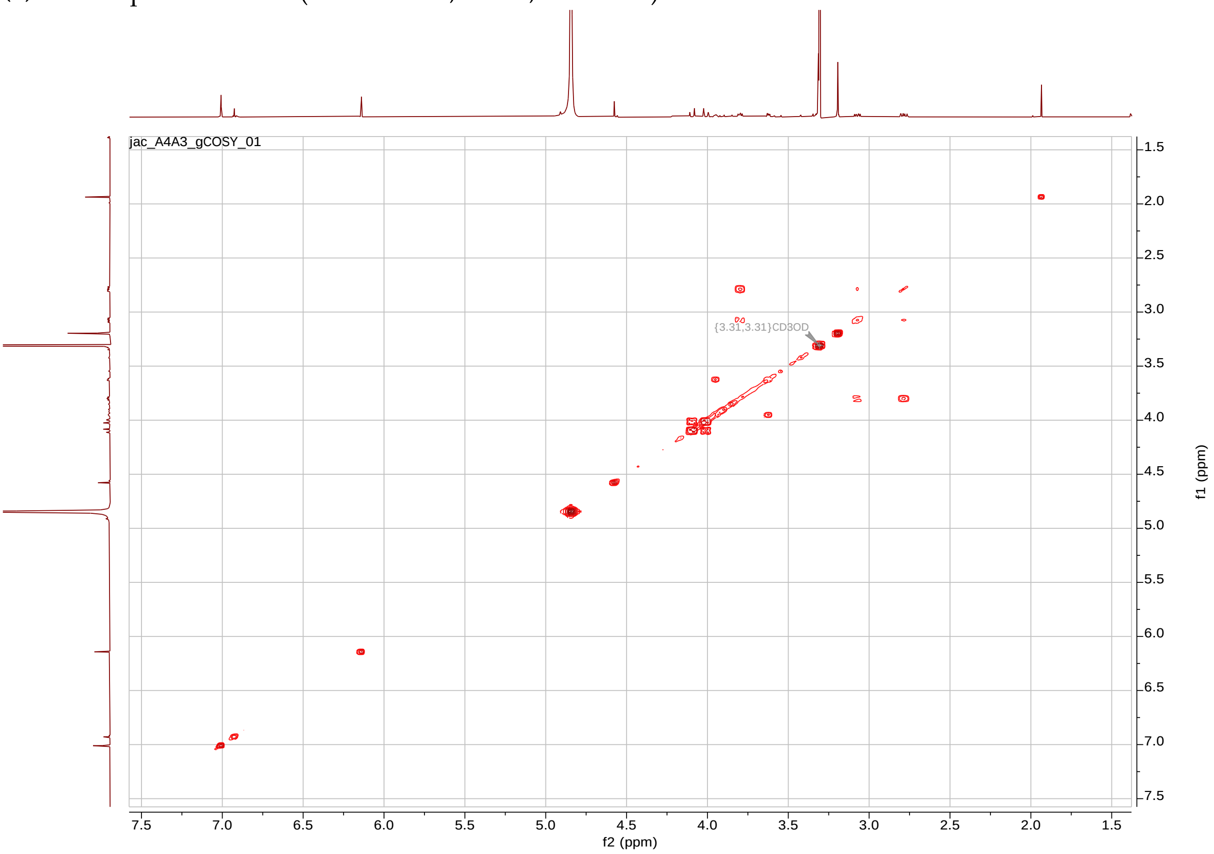

Figure S5. 1D and 2D-NMR spectra of **15**.

(a) ^{1}H-NMR spectrum of **15** (1:1 (*v/v*) methanol-*d*$_4$ / water-*d*$_2$, 289 K, 600 MHz).

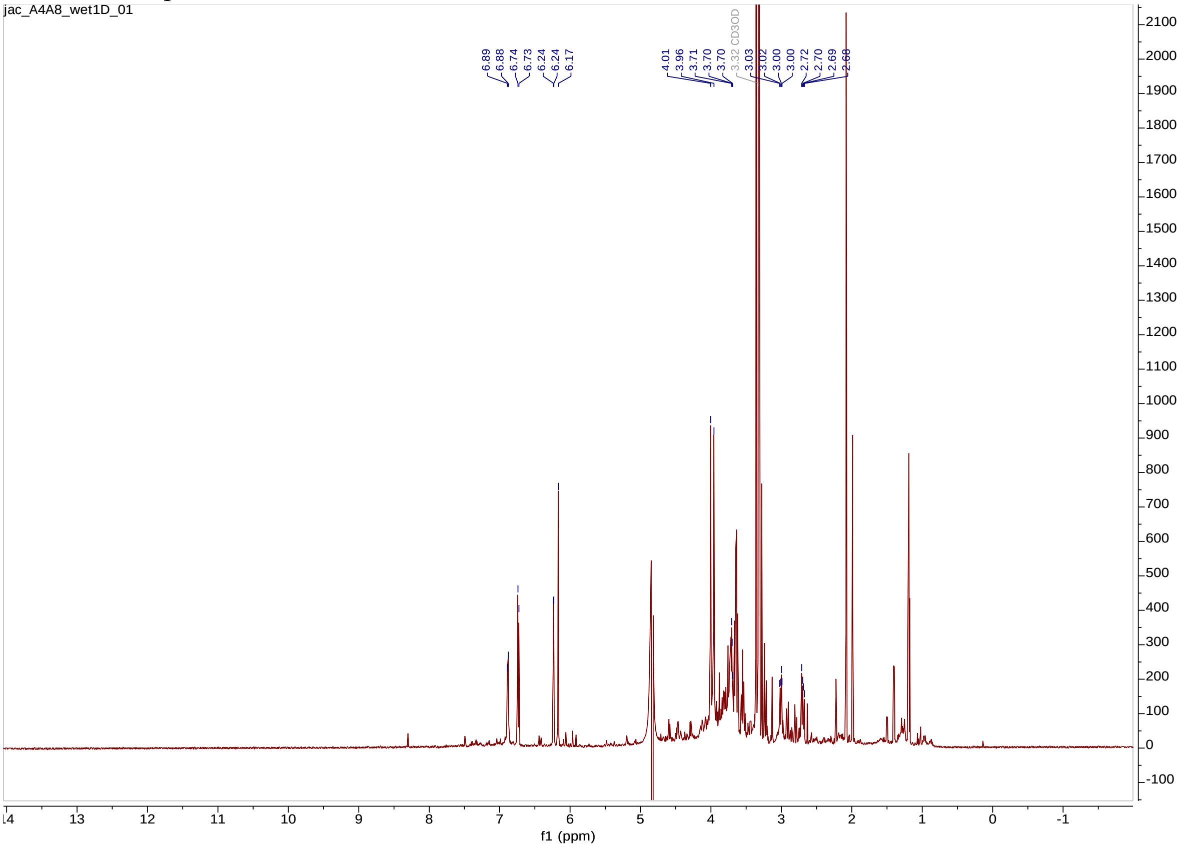

(b) ^{13}C-NMR spectrum of **15** (1:1 (*v/v*) methanol-*d*$_4$ / water-*d*$_2$, 289 K, 600 MHz).

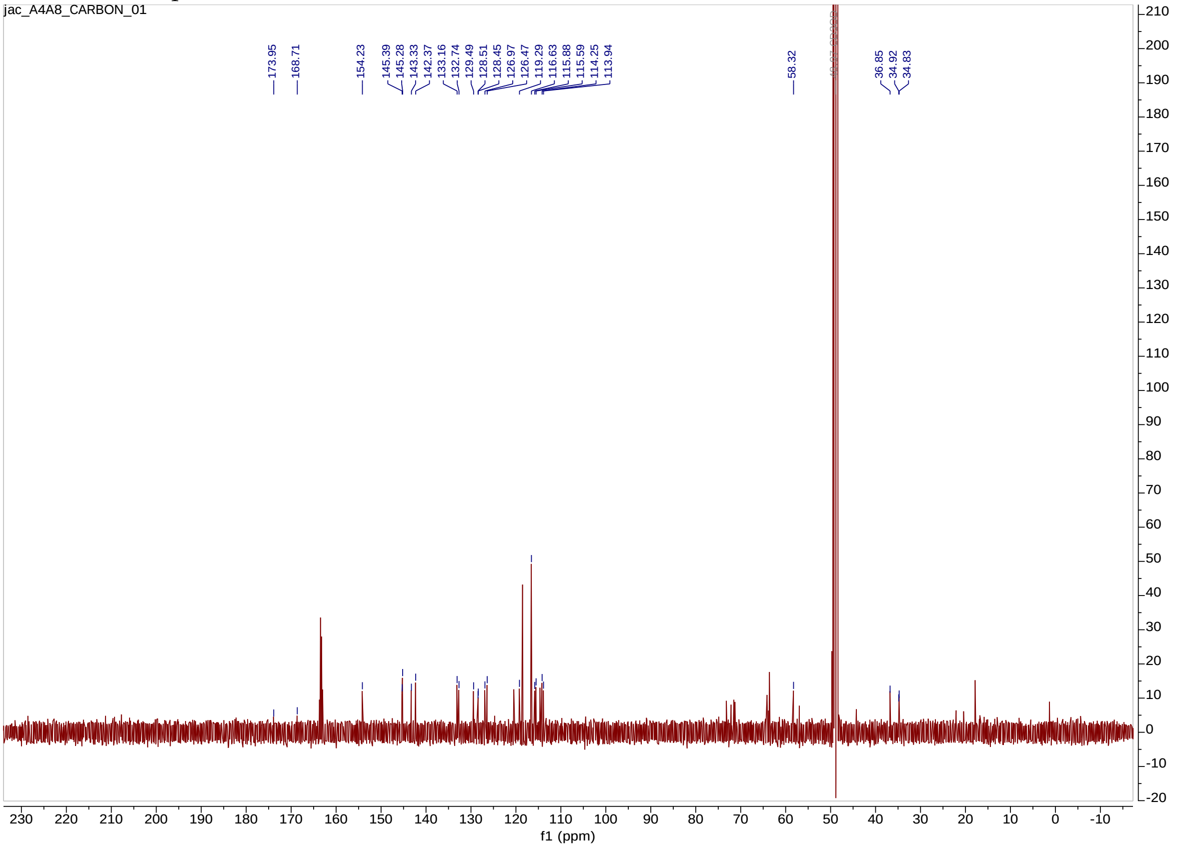

(c) HSQC spectrum of **15** (1:1 (*v*/*v*) methanol-*d*4 / water-*d*2, 289 K, 600 MHz).

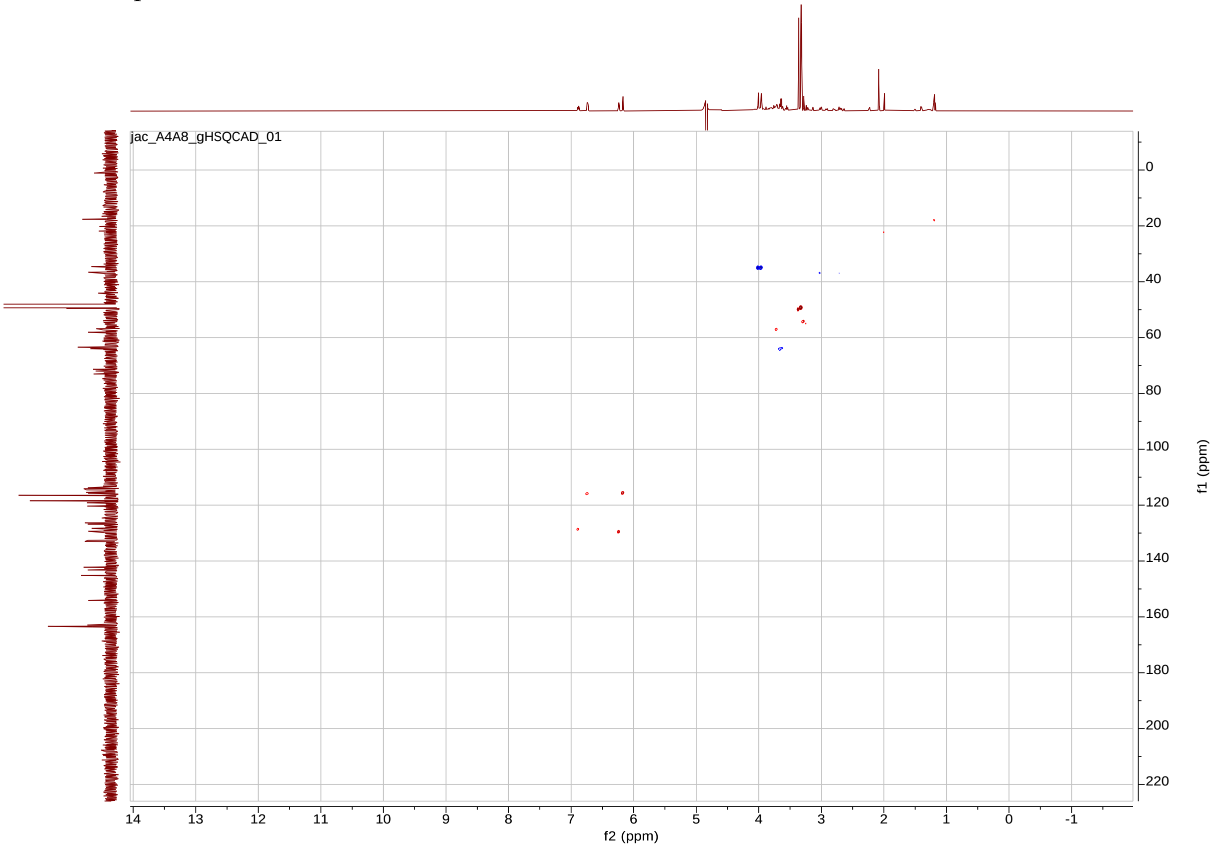

(d) HMBC spectrum of **15** (1:1 (*v*/*v*) methanol-*d*4 / water-*d*2, 289 K, 600 MHz).

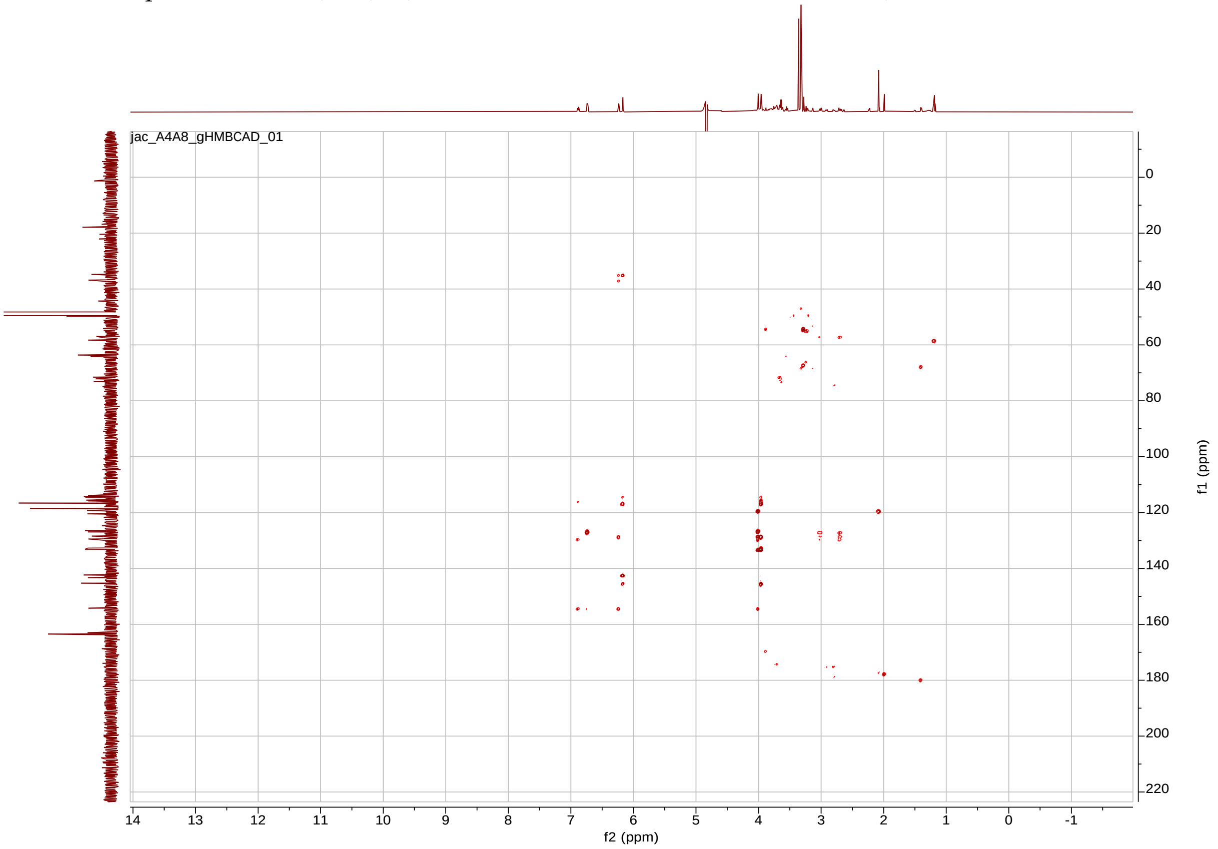

(e) COSY spectrum of **15** (1:1 (*v*/*v*) methanol-d_4 / water-d_2, 289 K, 600 MHz).

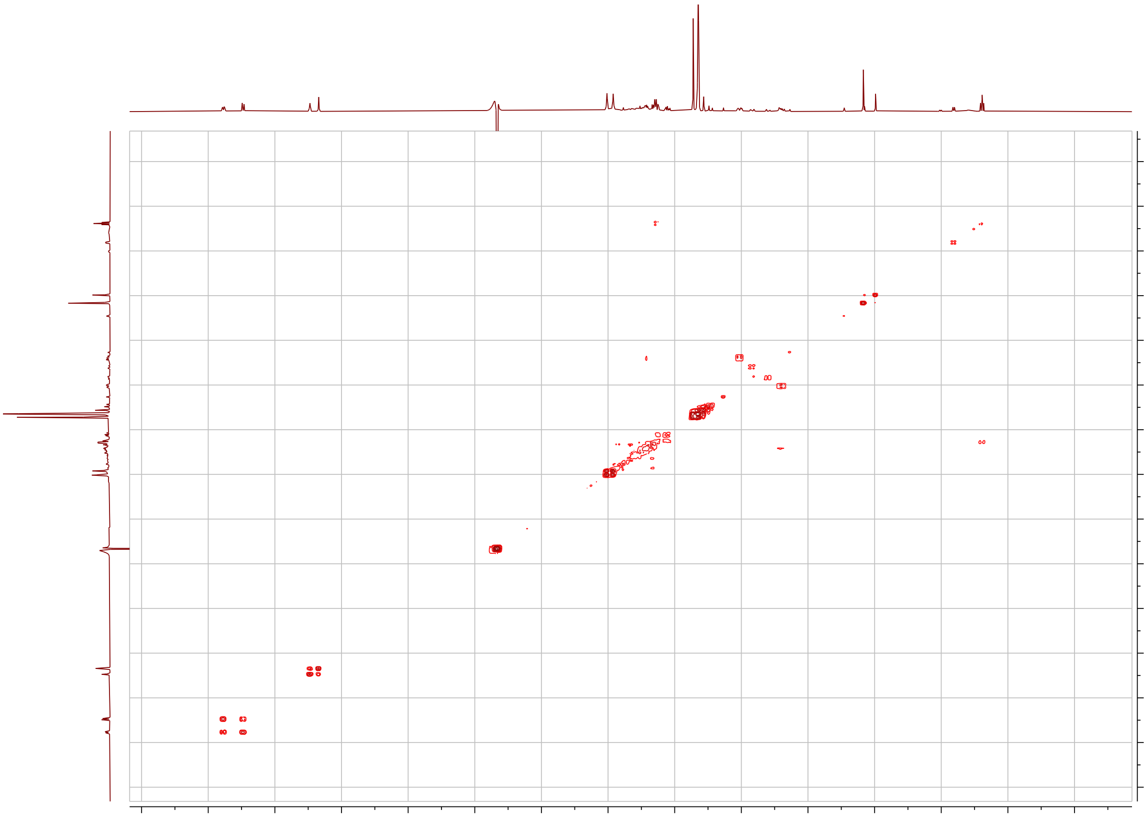

Figure S6. 1D and 2D-NMR spectra of **16**.

(a) ^{1}H-NMR spectrum of **16** (methanol-d_4, 289 K, 600 MHz).

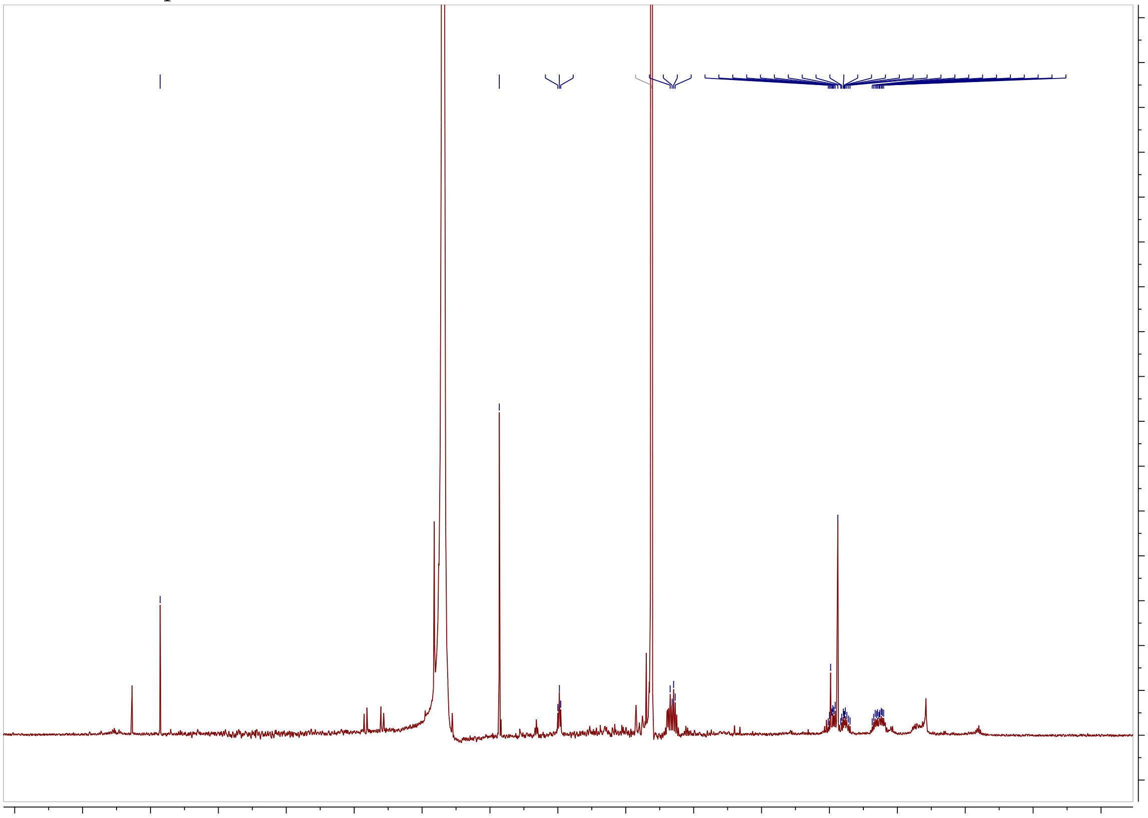

(b) ^{13}C-NMR spectrum of **16** (methanol-d_4, 289 K, 151 MHz).

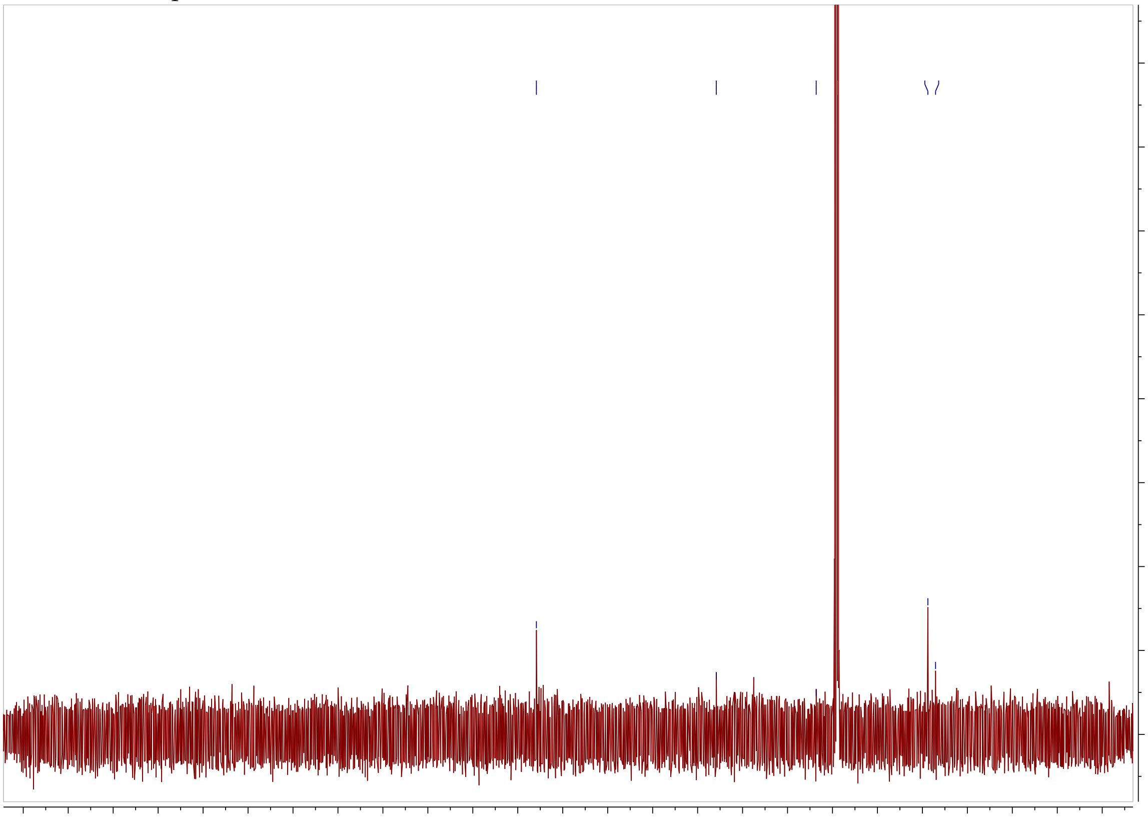

(c) HSQC spectrum of **16** (methanol-d_4, 289 K, 600 MHz).

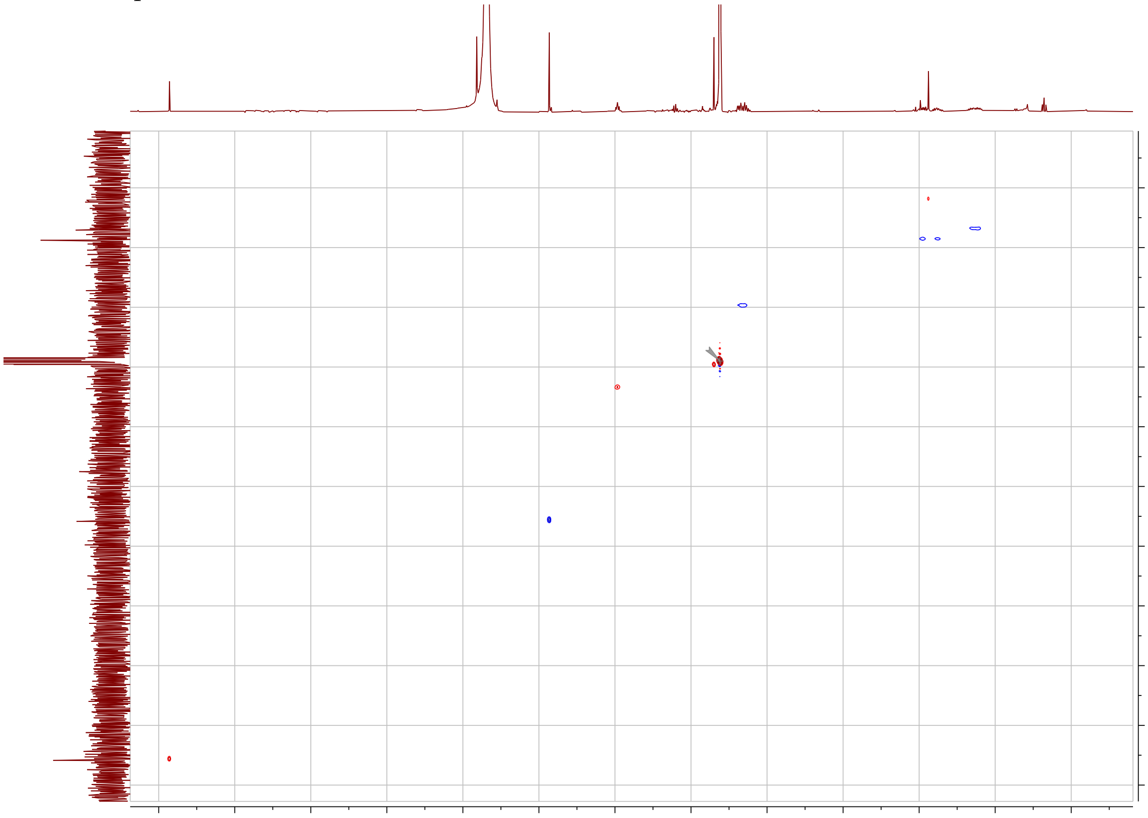

(d) HMBC spectrum of **16** (methanol-d_4, 289 K, 600 MHz).

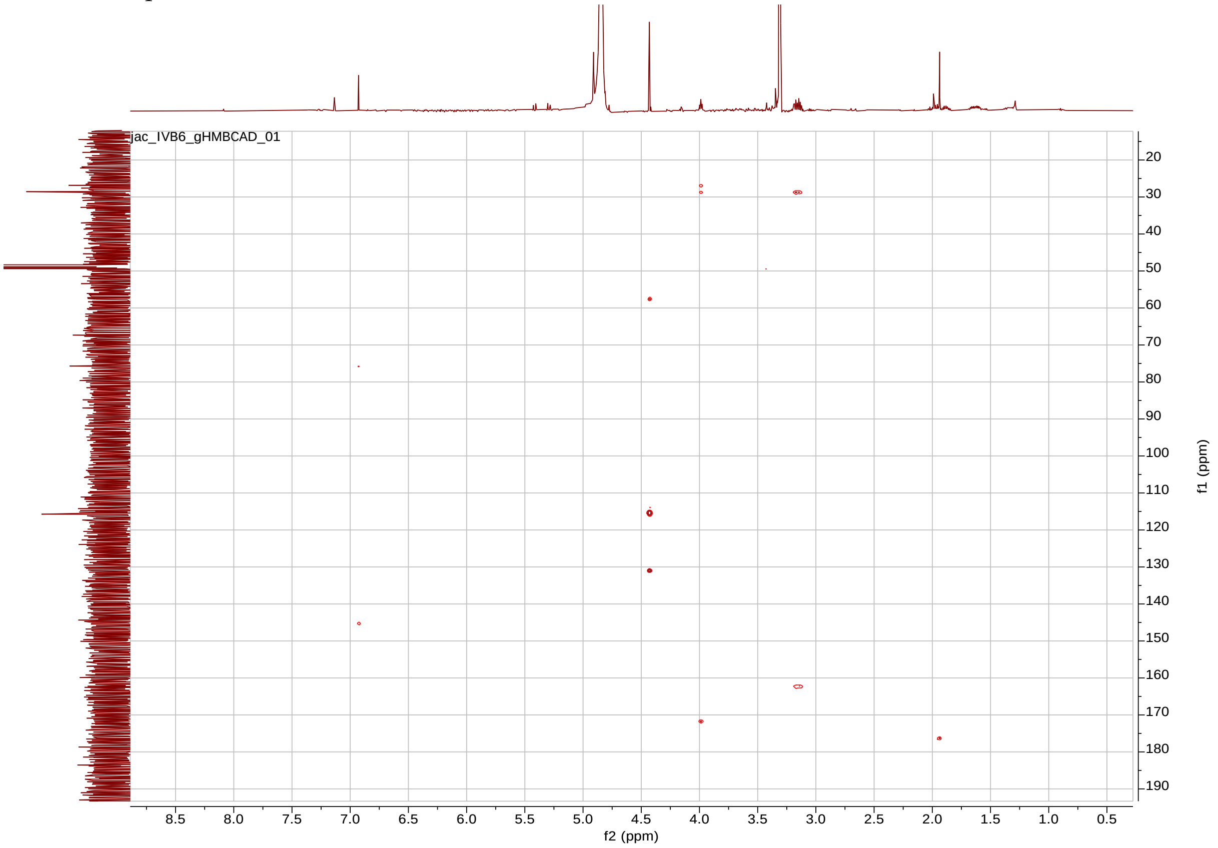

(e) COSY spectrum of **16** (methanol-d_4, 289 K, 600 MHz).

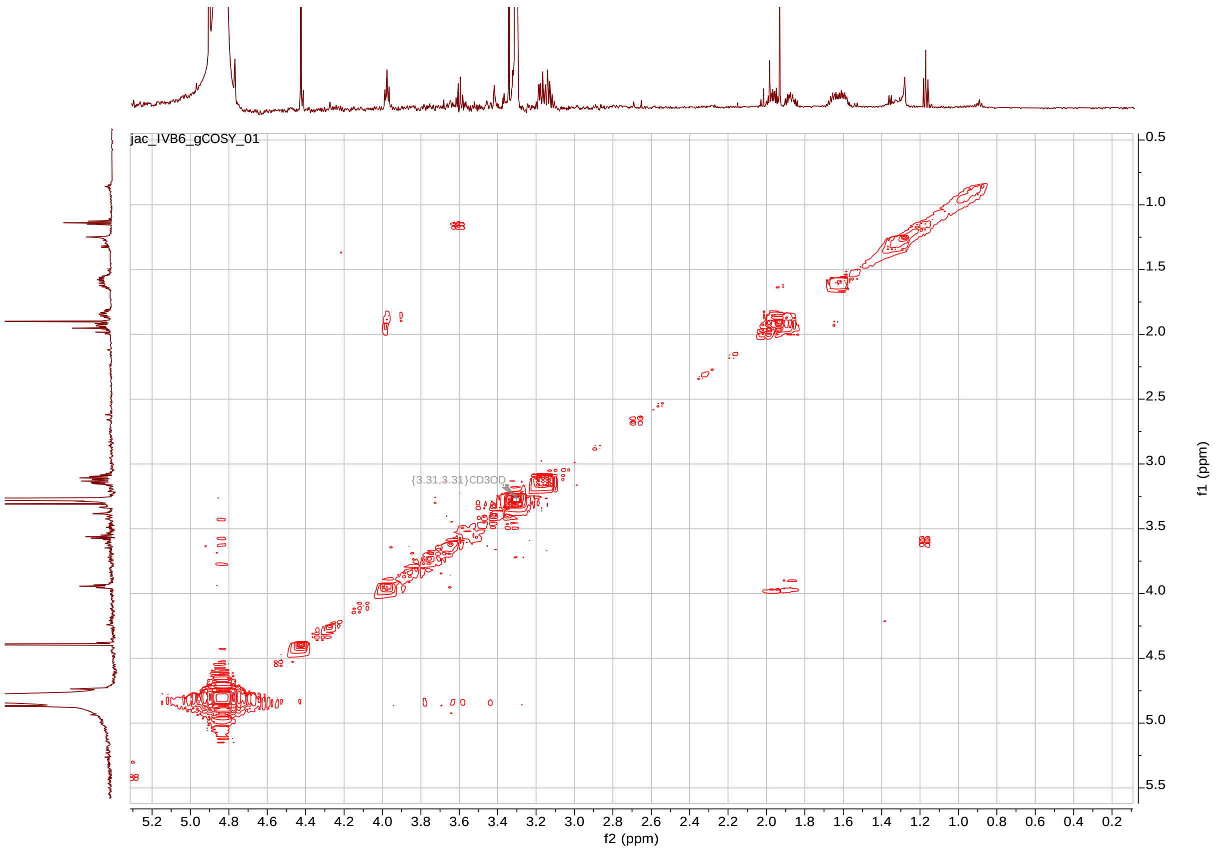

Figure S7. 1D and 2D-NMR spectra of **23**.

(a) ^{1}H-NMR spectrum of **23** (methanol-d_4, 289 K, 600 MHz).

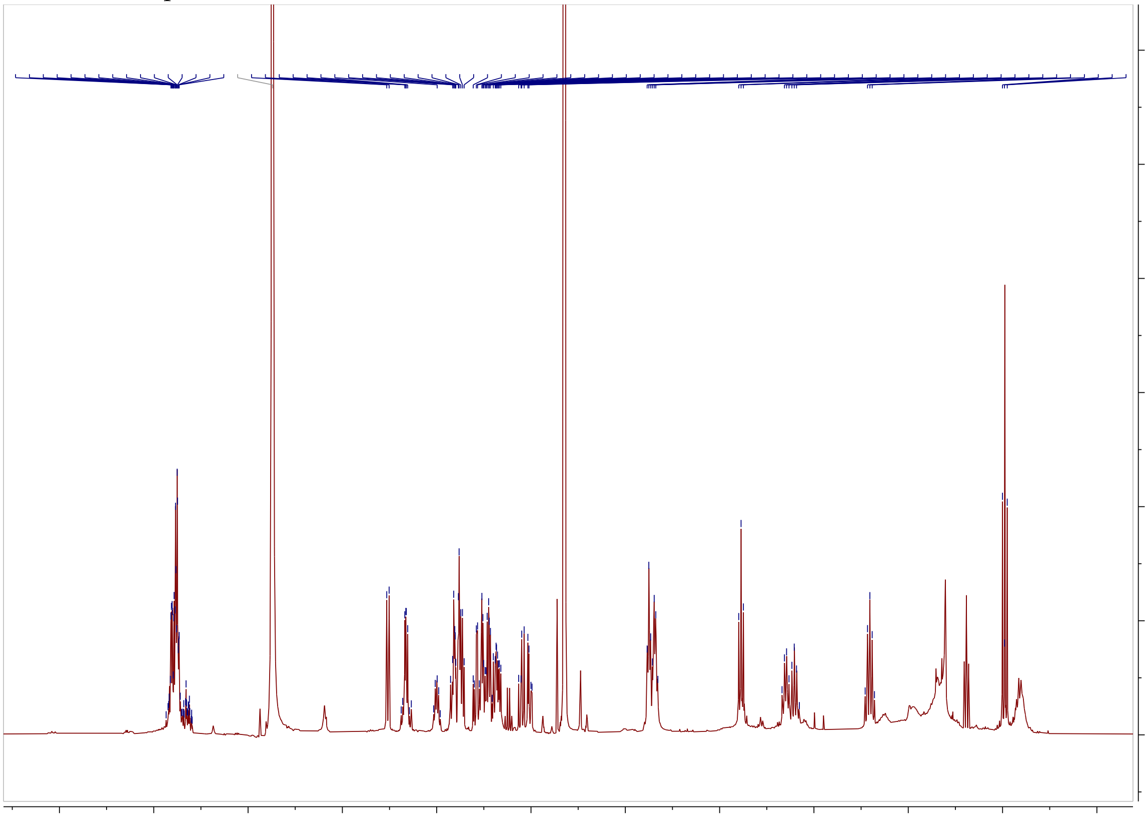

(b) ^{13}C-NMR spectrum of **23** (methanol-d_4, 289 K, 151 MHz).

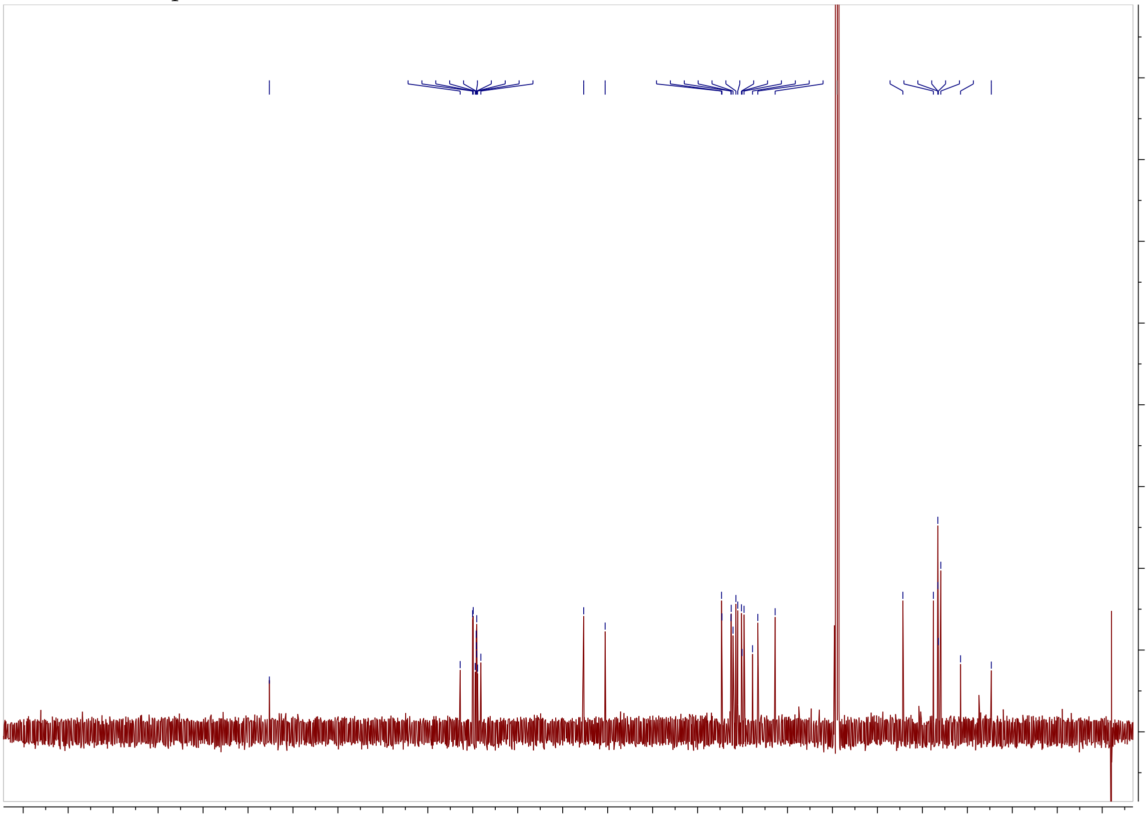

(c) HSQC spectrum of **23** (methanol-*d*4, 289 K, 600 MHz).

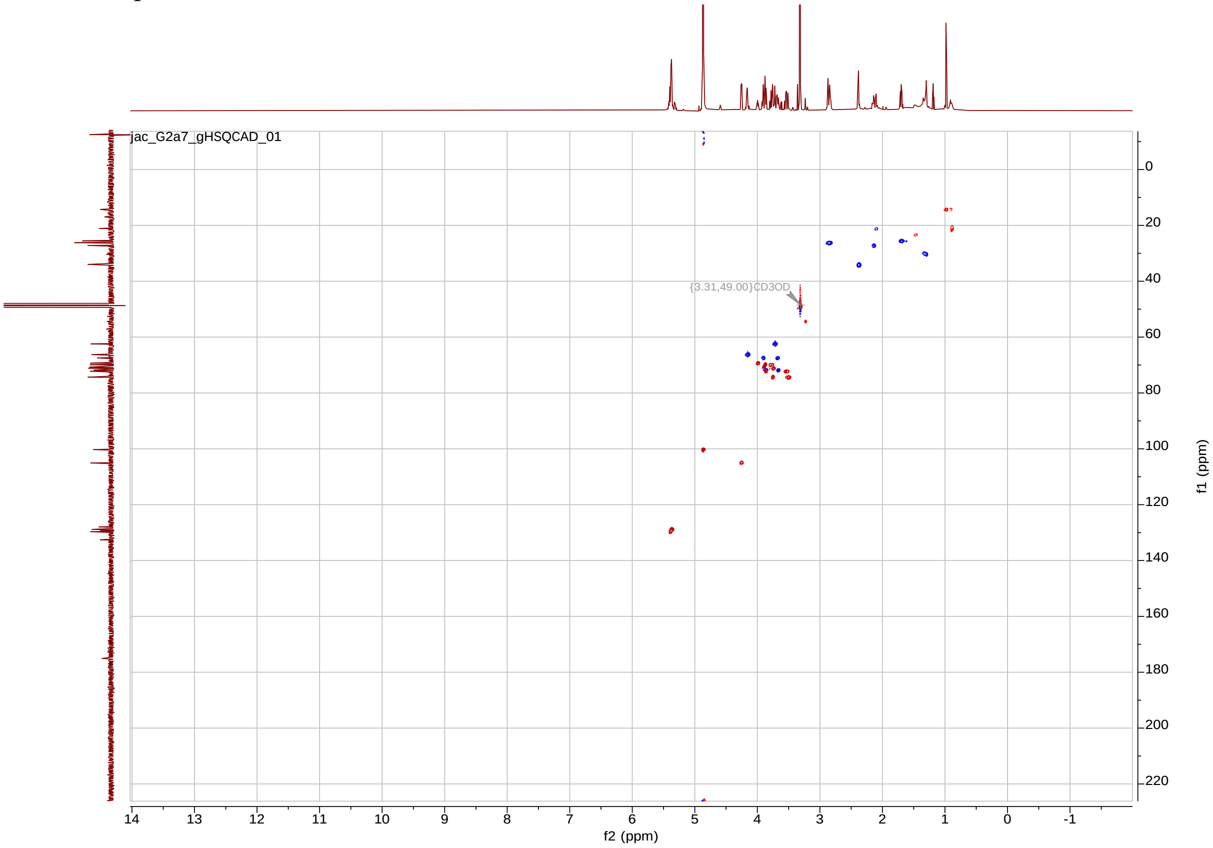

(d) HMBC spectrum of **23** (methanol-*d*4, 289 K, 600 MHz).

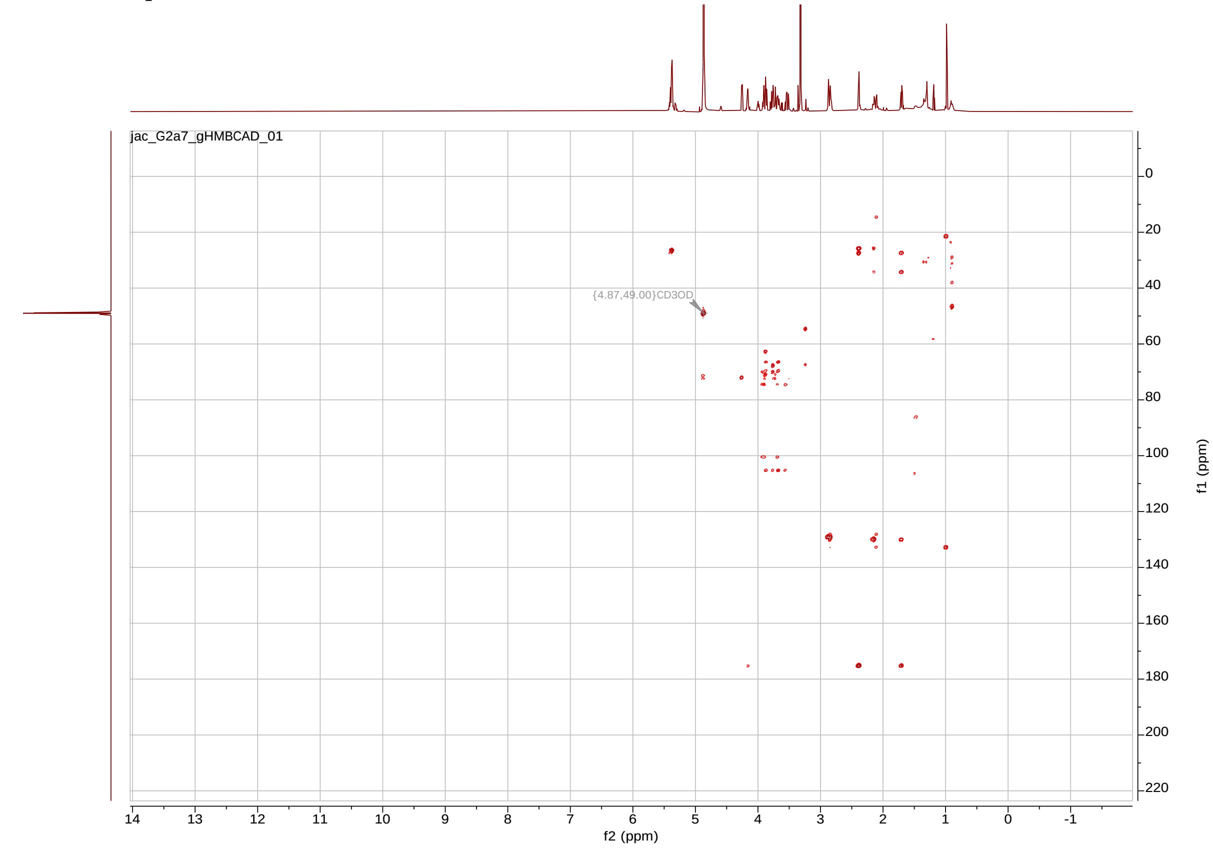

(e) COSY spectrum of **23** (methanol-d_4, 289 K, 600 MHz).

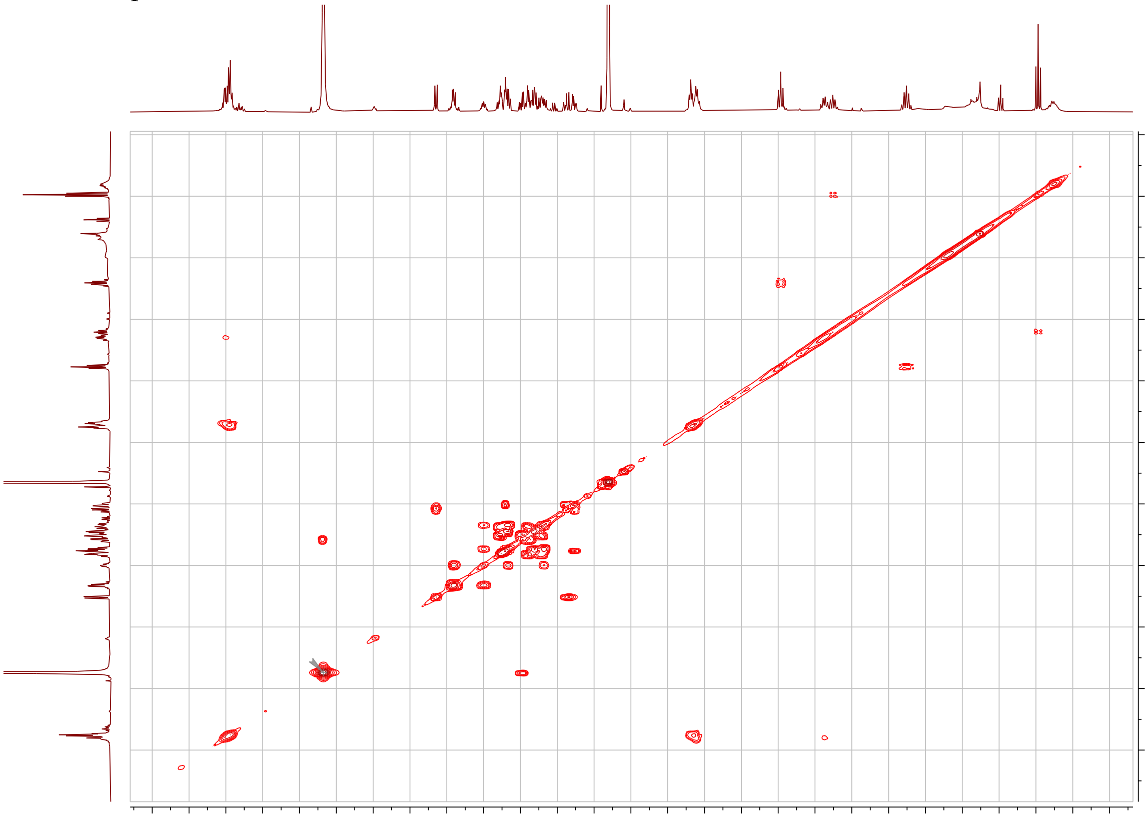

(f) ^{1}H-NMR spectrum of **23** (acetone-d_6, 289 K, 600 MHz).

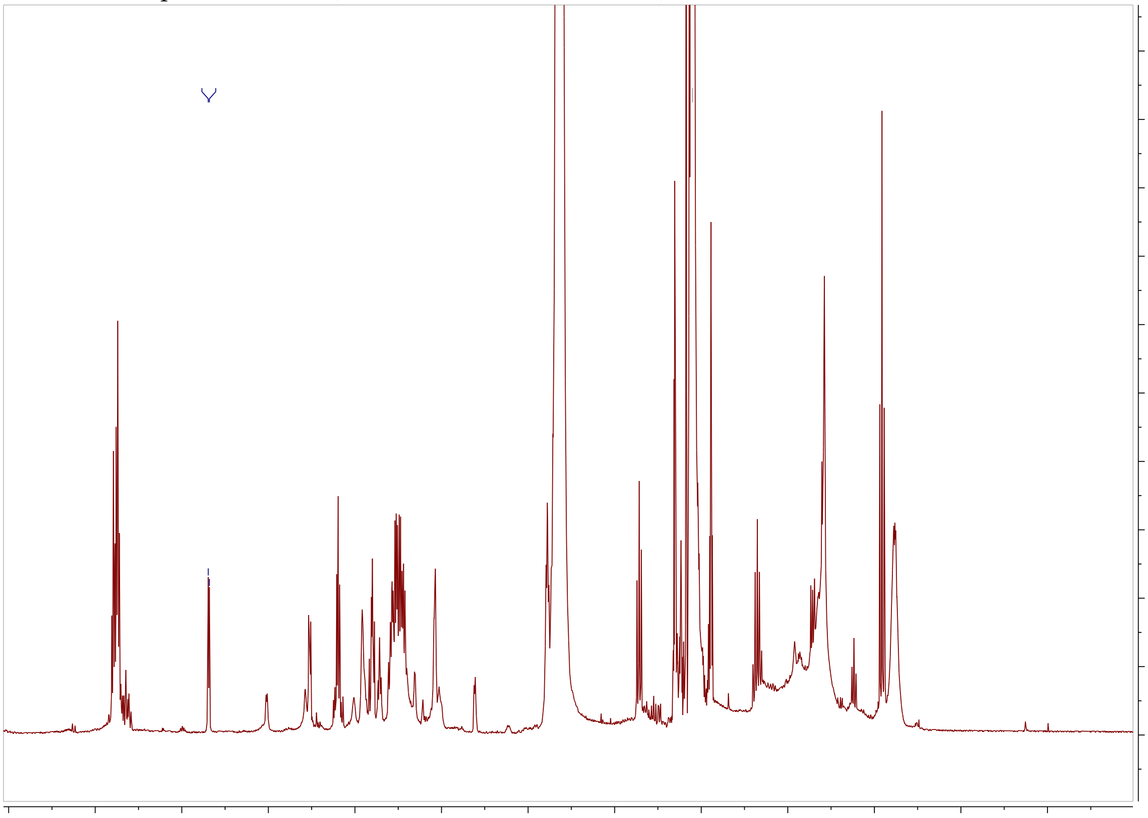

HR-ESI-MS spectra of new compounds

Figure S8. HR-ESI-MS spectra of **7**.

(a) HR-(+)ESI-MS spectrum of **7** full view.

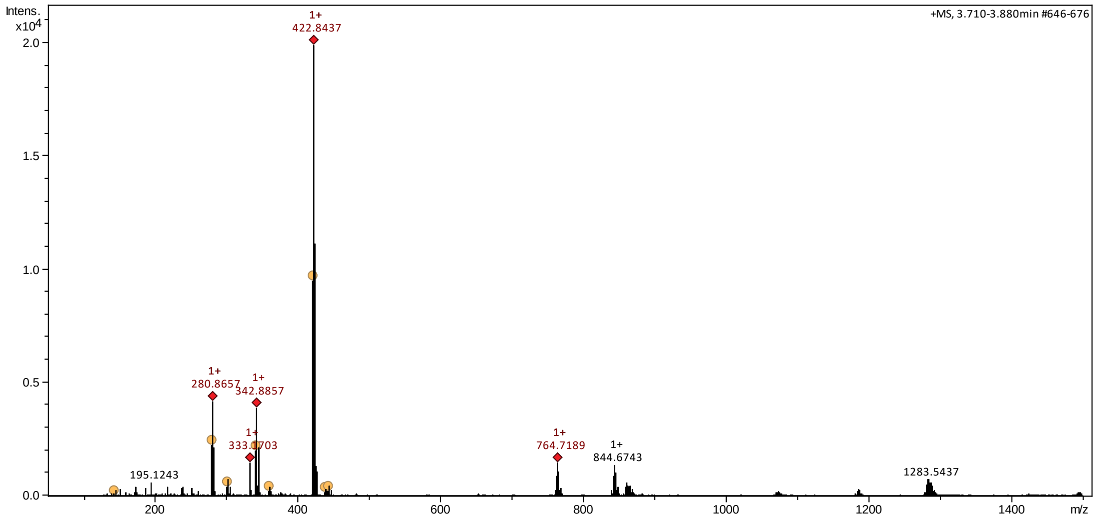

(b) HR-(+)ESI-MS spectrum of **7** zoomed in to show isotopic patterns and fragments.

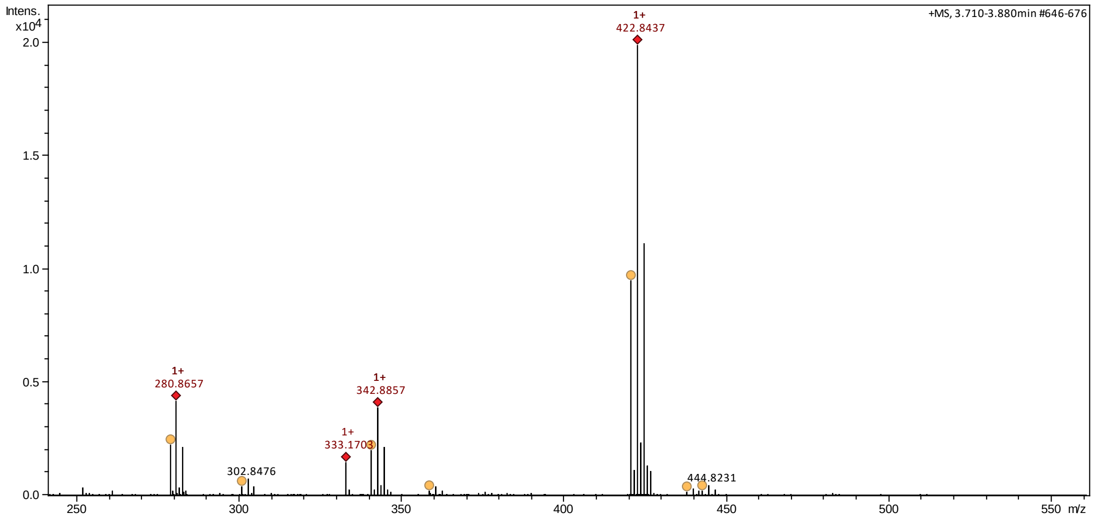

(c) HR-(-)ESI-MS spectrum of **7**.

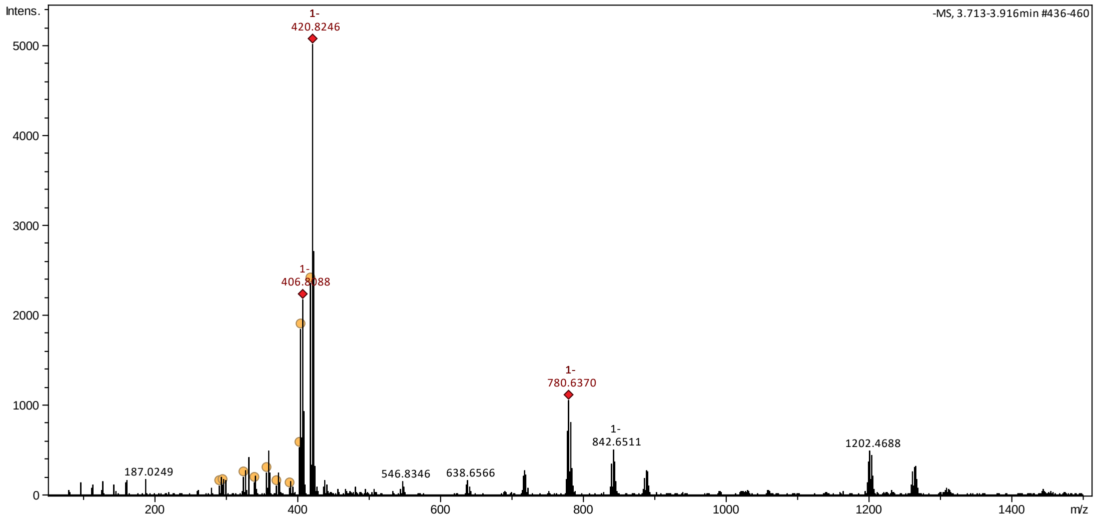

Figure S9. HR-(+)ESI-MS spectrum of **12**.

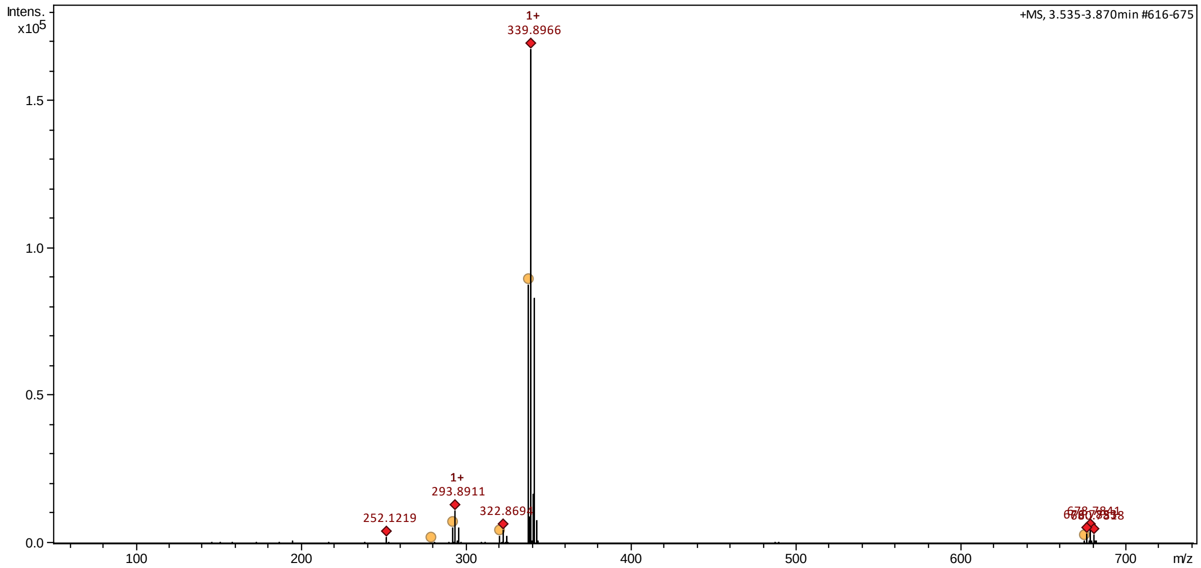

Figure S10. HR-(-)ESI-MS spectrum of **13**.

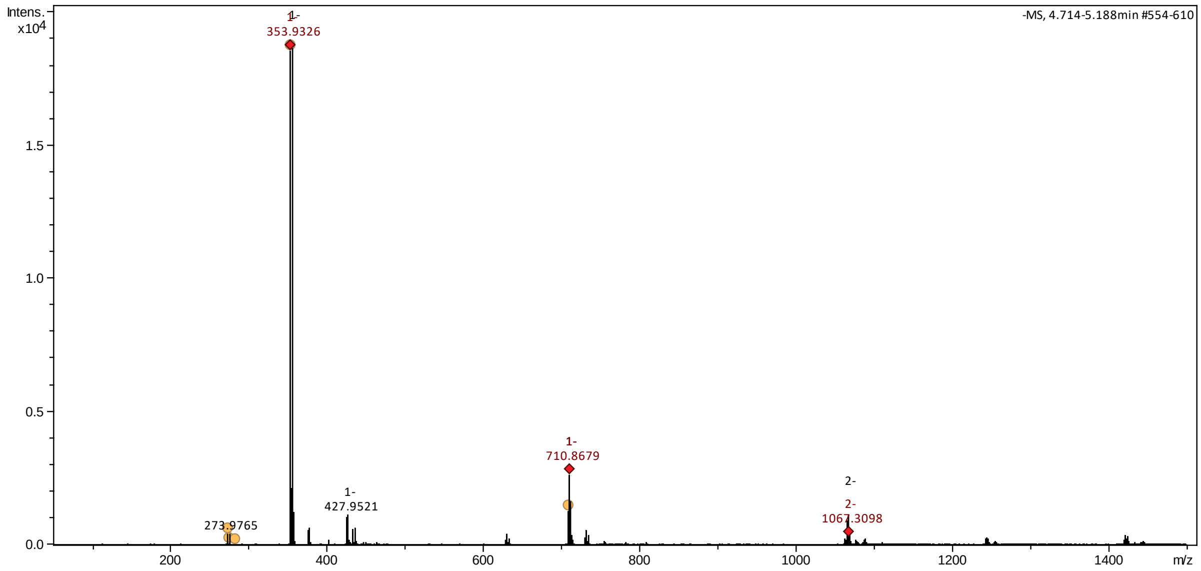

Figure S11. HR-(-)ESI-MS spectrum of **14**.

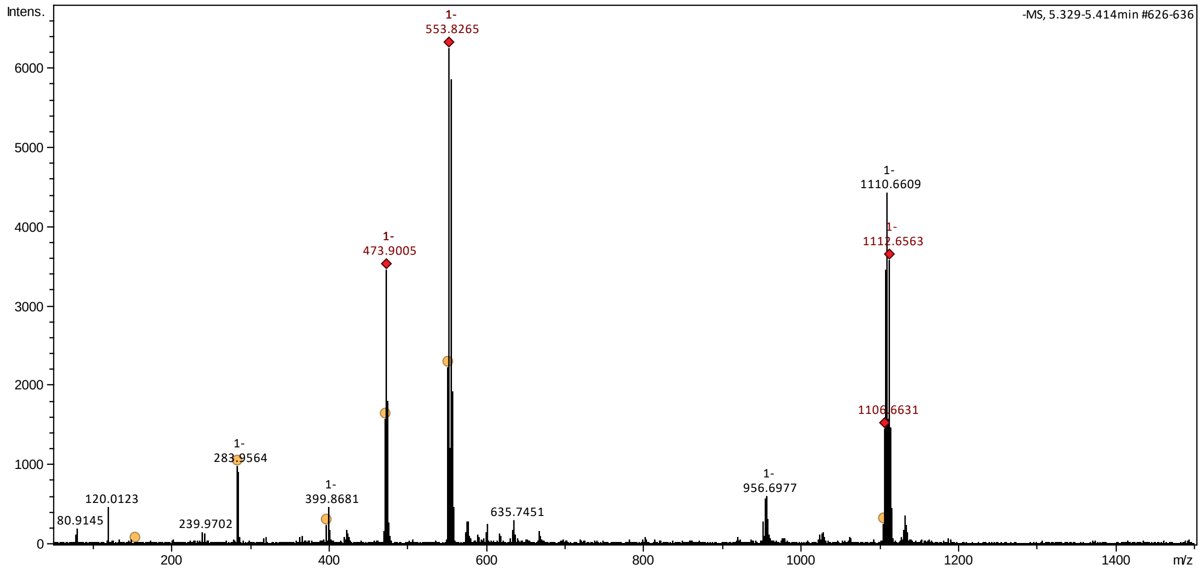

Figure S12. HR-(-)ESI-MS spectrum of **15**.

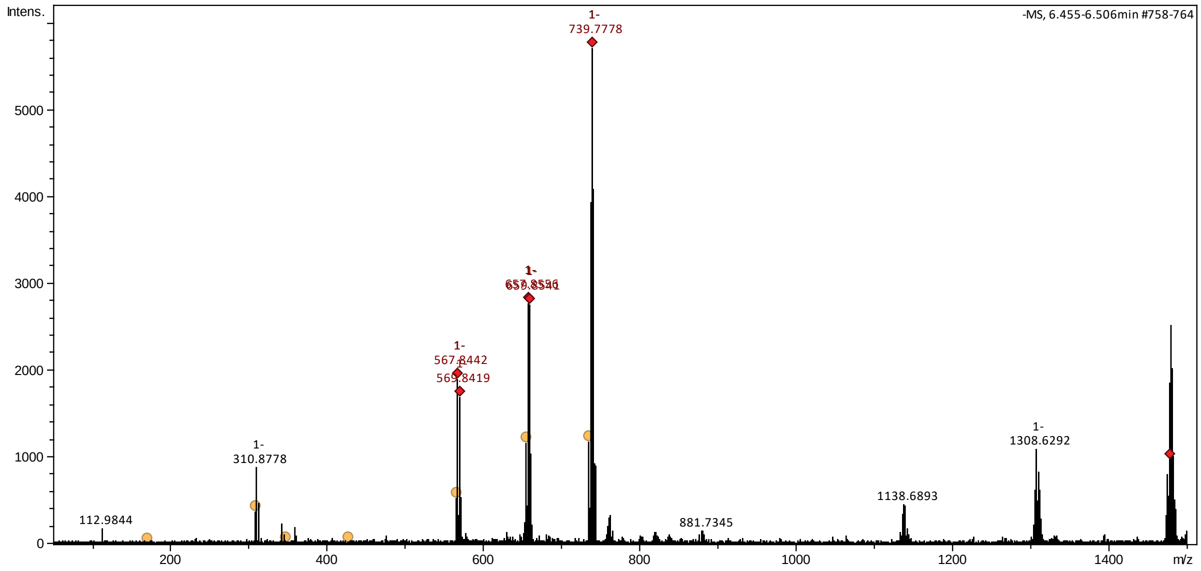

Figure S13. HR-(+)ESI-MS spectrum of **16**.

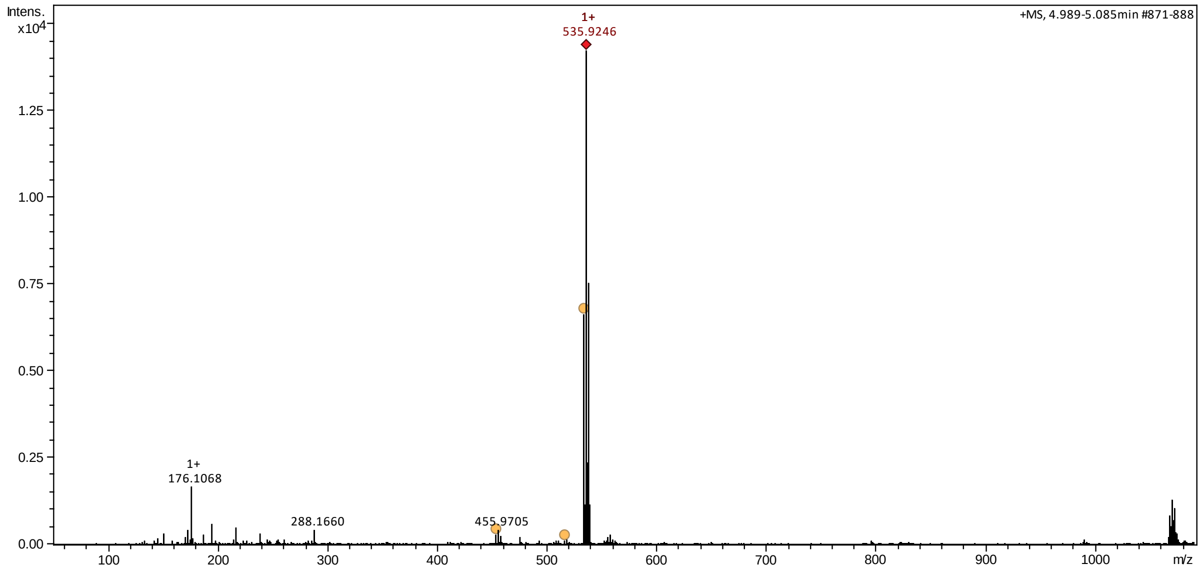

Figure S14. HR-(+)ESI-MS spectrum of **23**.

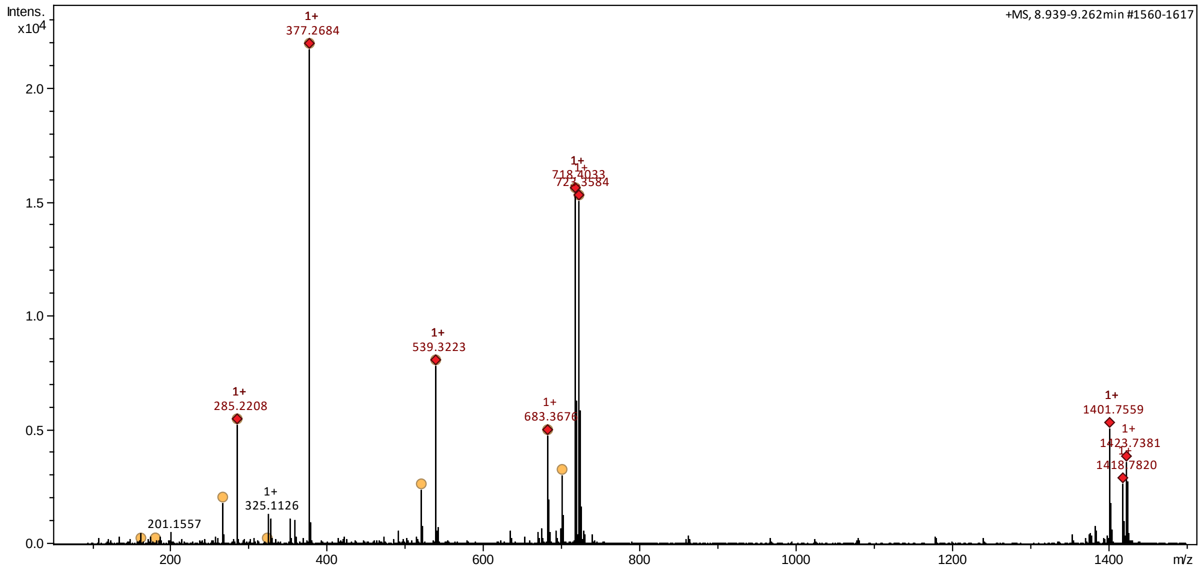

^{1}H-NMR spectra of previously reported compounds

Figure S15. 1H-NMR spectrum of **1** (methanol-d_4, 289 K, 600 MHz).

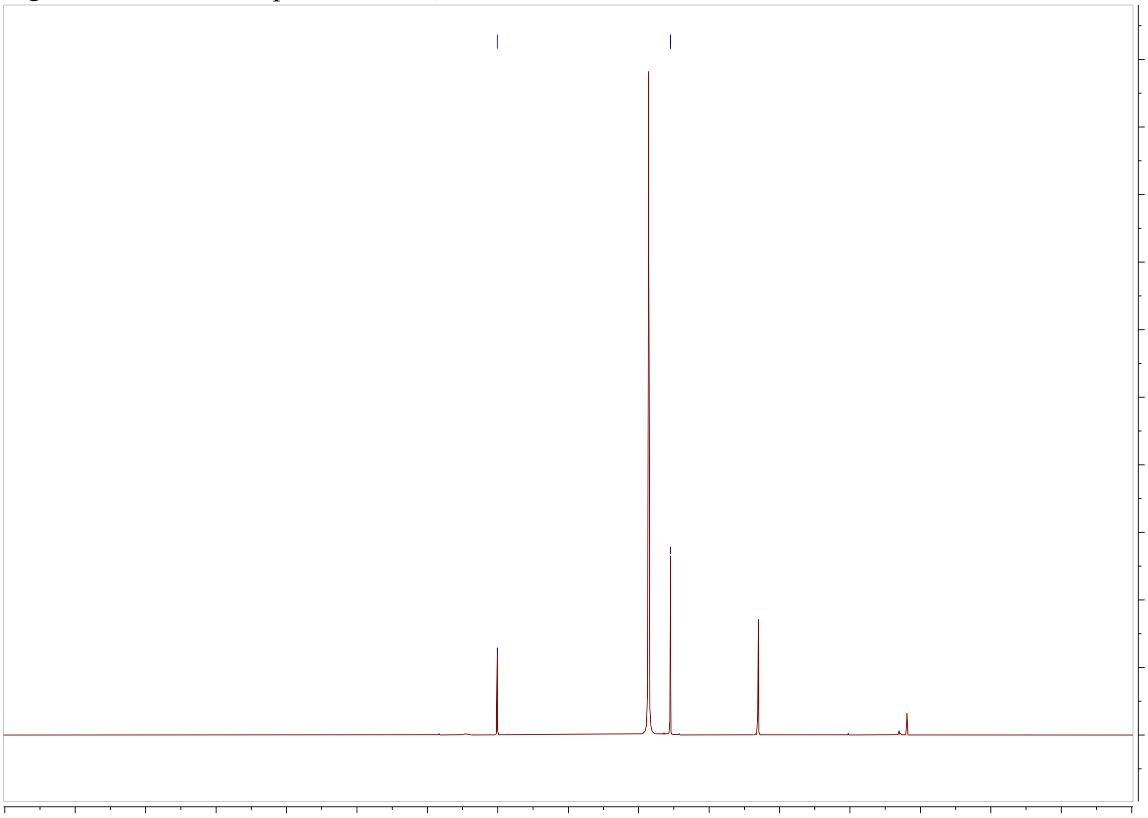

Figure S16. 1H-NMR spectrum of **2** (methanol-d_4, 289 K, 600 MHz).

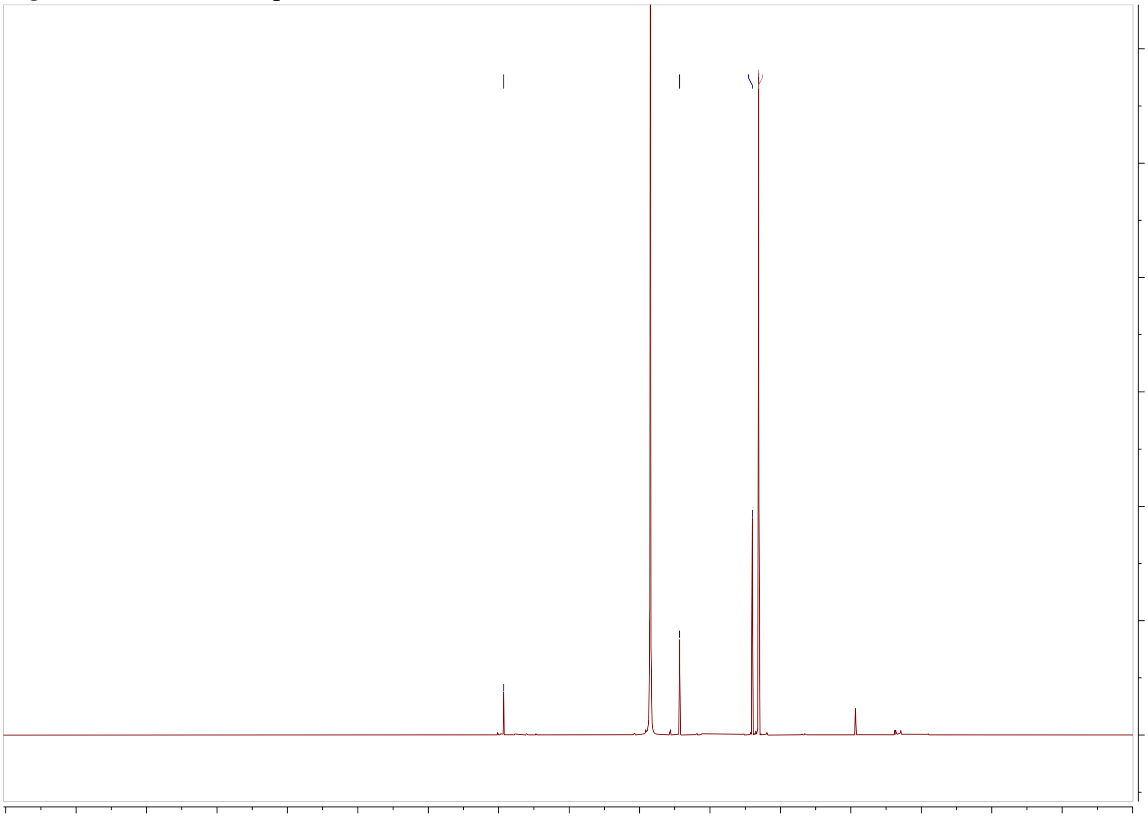

Figure S17. 1H-NMR spectrum of **3** (acetone-*d*6, 289 K, 600 MHz).

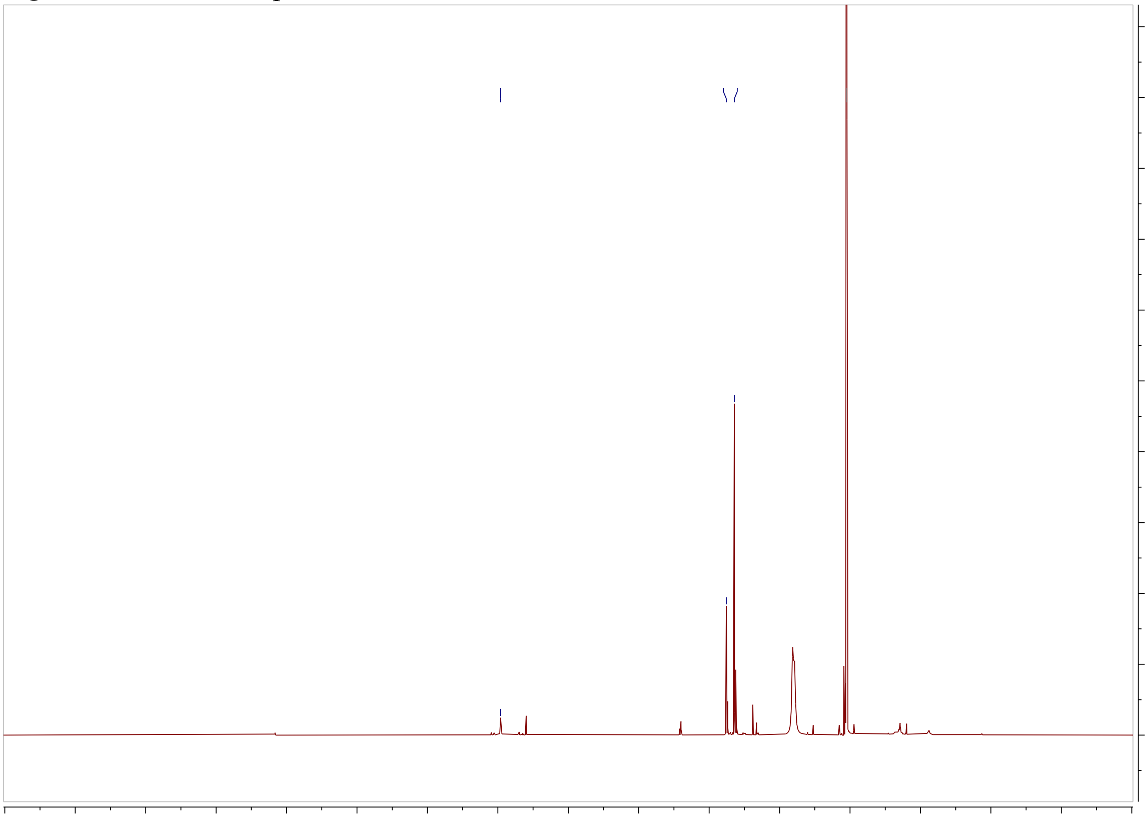

Figure S18. 1H-NMR spectrum of **4** (water-*d*2, 289 K, 400 MHz).

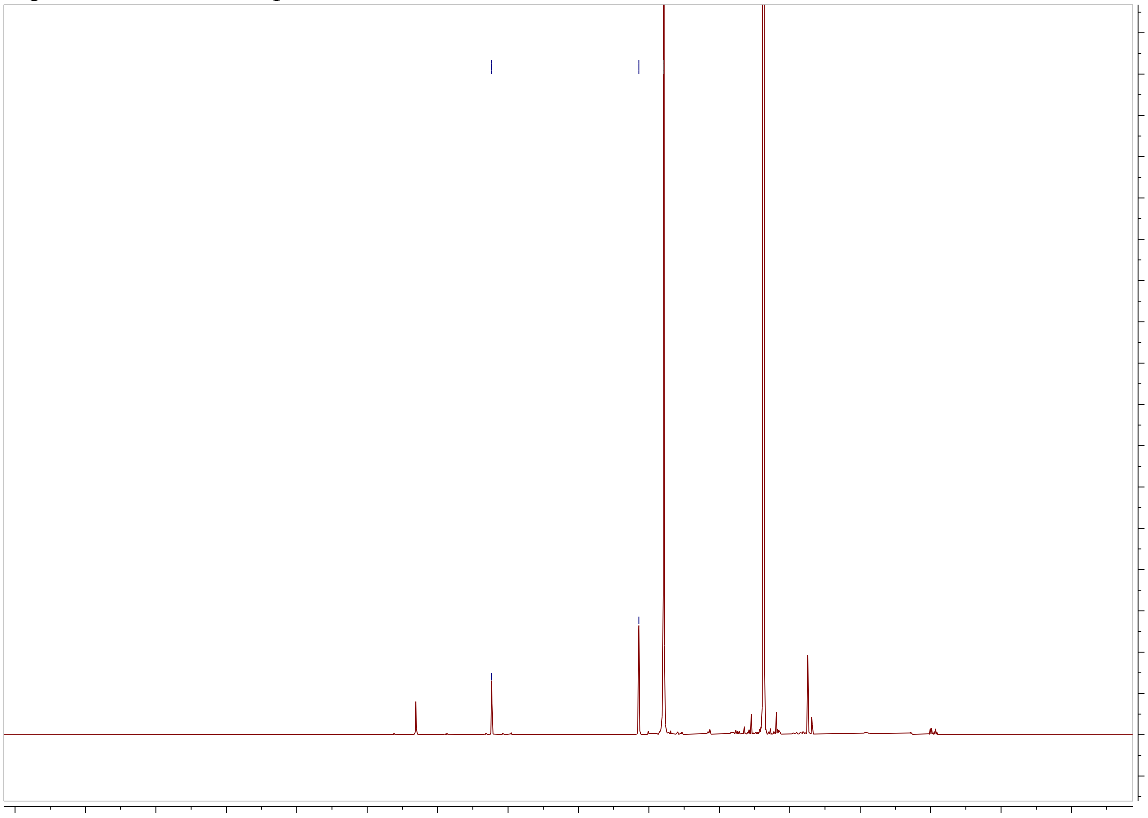

Figure S19. 1H-NMR spectrum of **5** (acetone-d_6, 289 K, 600 MHz).

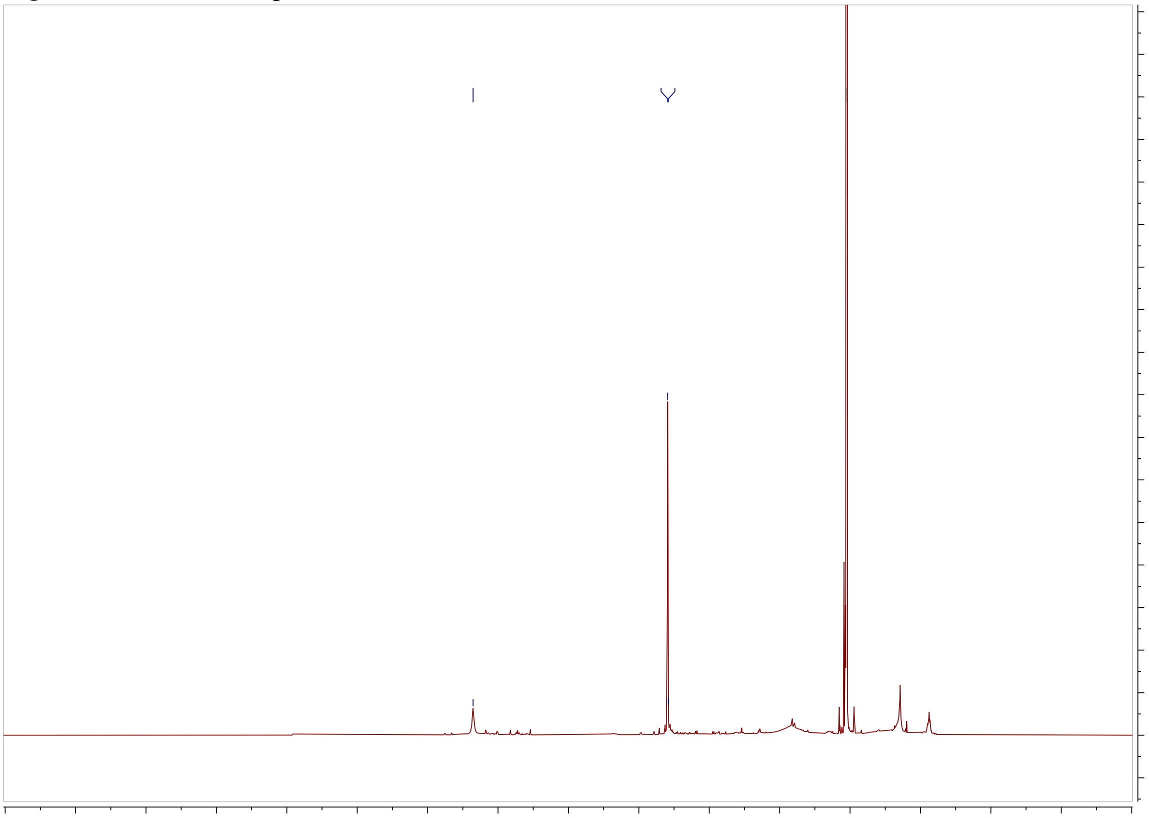

Figure S20. 1H-NMR spectrum of **6** (acetone-d_6, 289 K, 600 MHz).

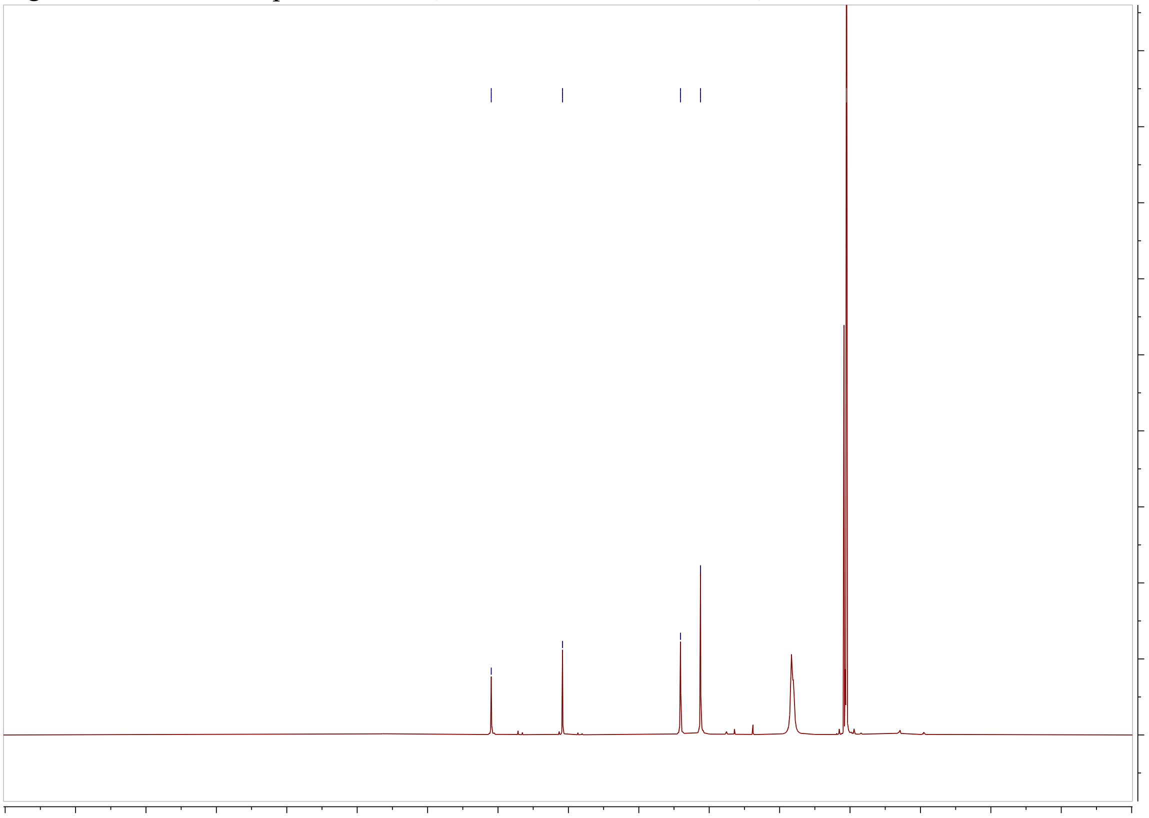

Figure S21. 1H-NMR spectrum of **8** (methanol-*d*4, 289 K, 600 MHz).

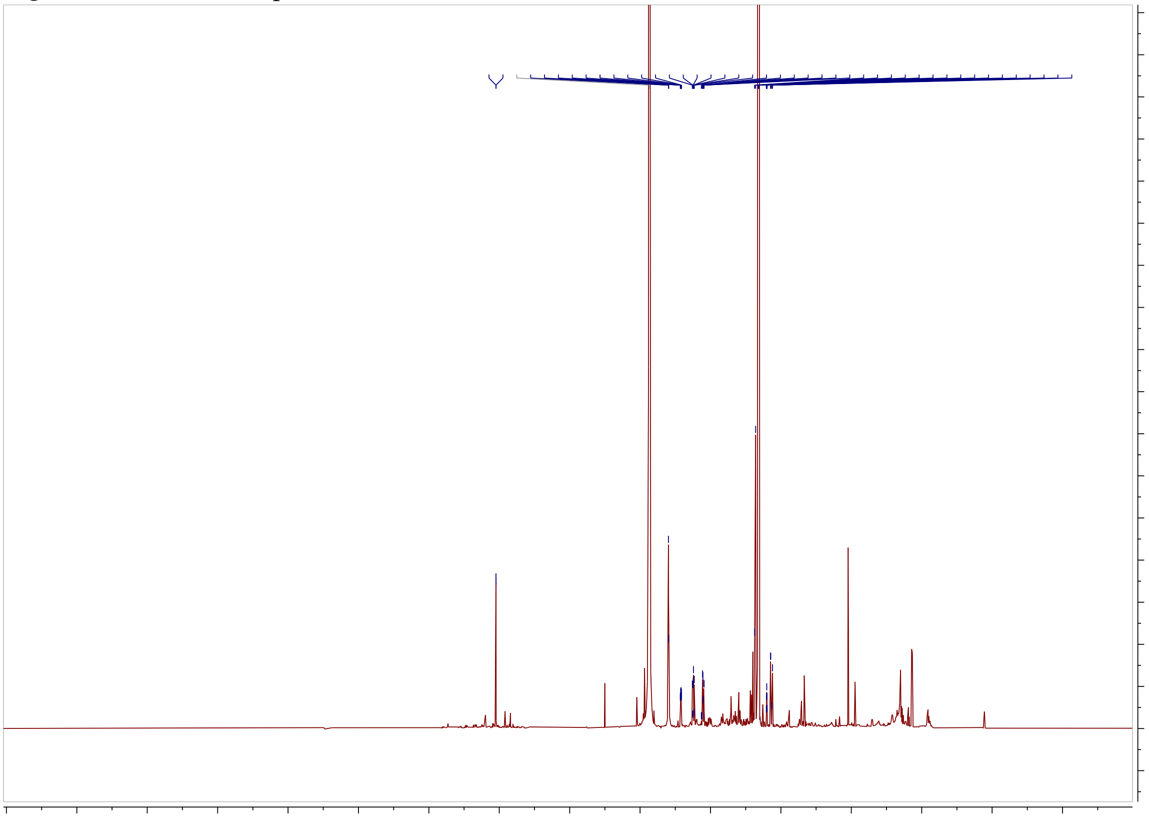

Figure S22. 1H-NMR spectrum of **9** (methanol-*d*4, 289 K, 600 MHz).

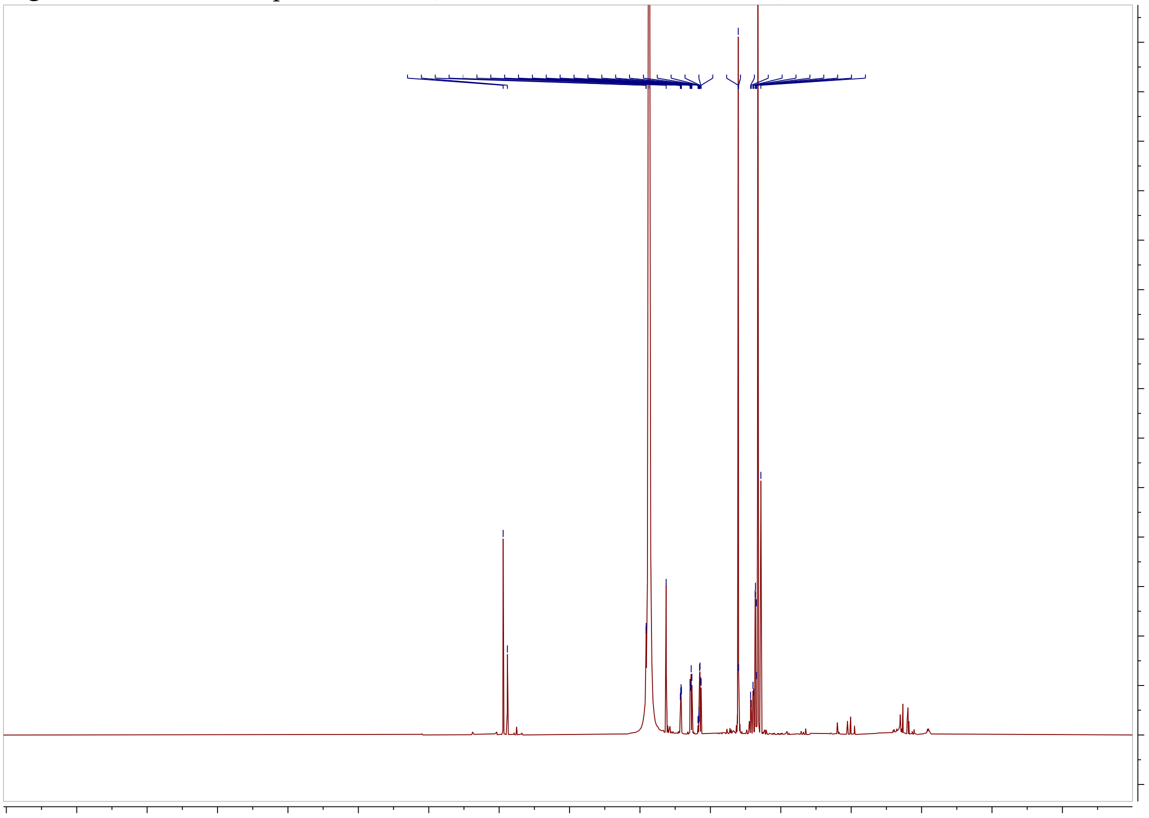

Figure S23. 1H-NMR spectrum of **10** (water-d_2, 289 K, 600 MHz).

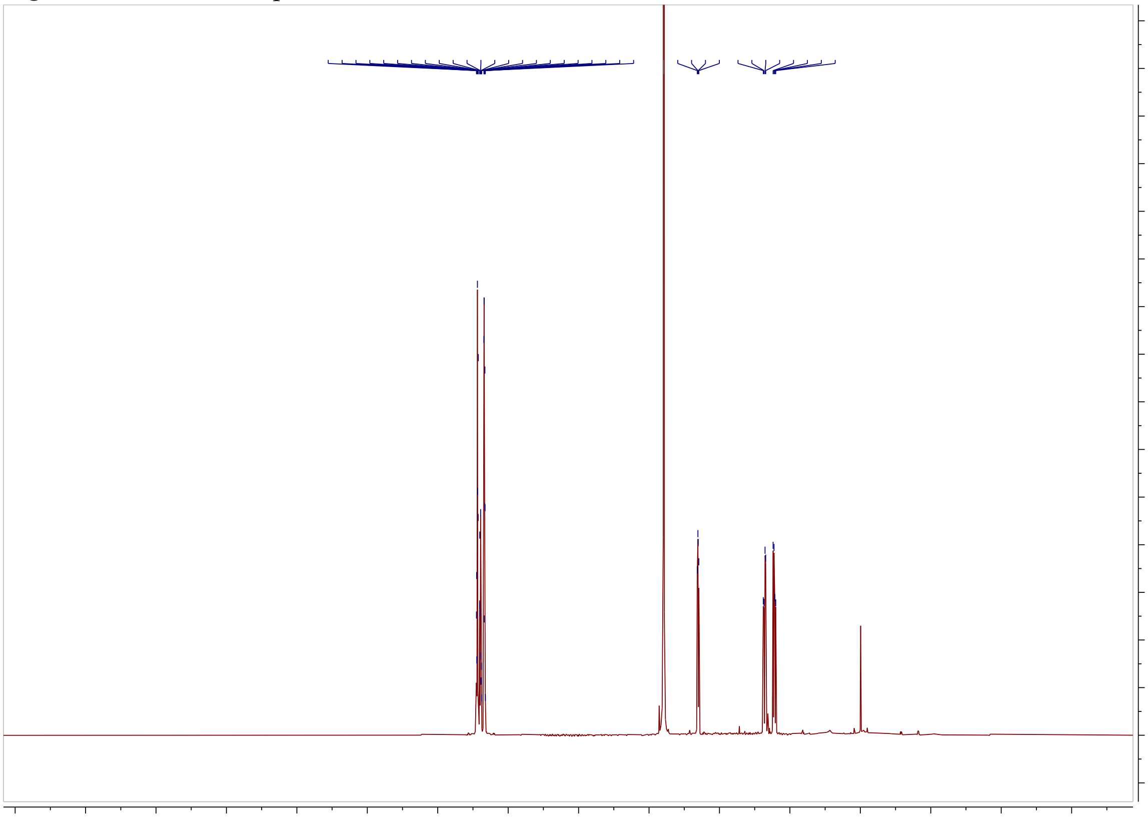

Figure S24. 1H-NMR spectrum of **11** (water-d_2, 289 K, 600 MHz).

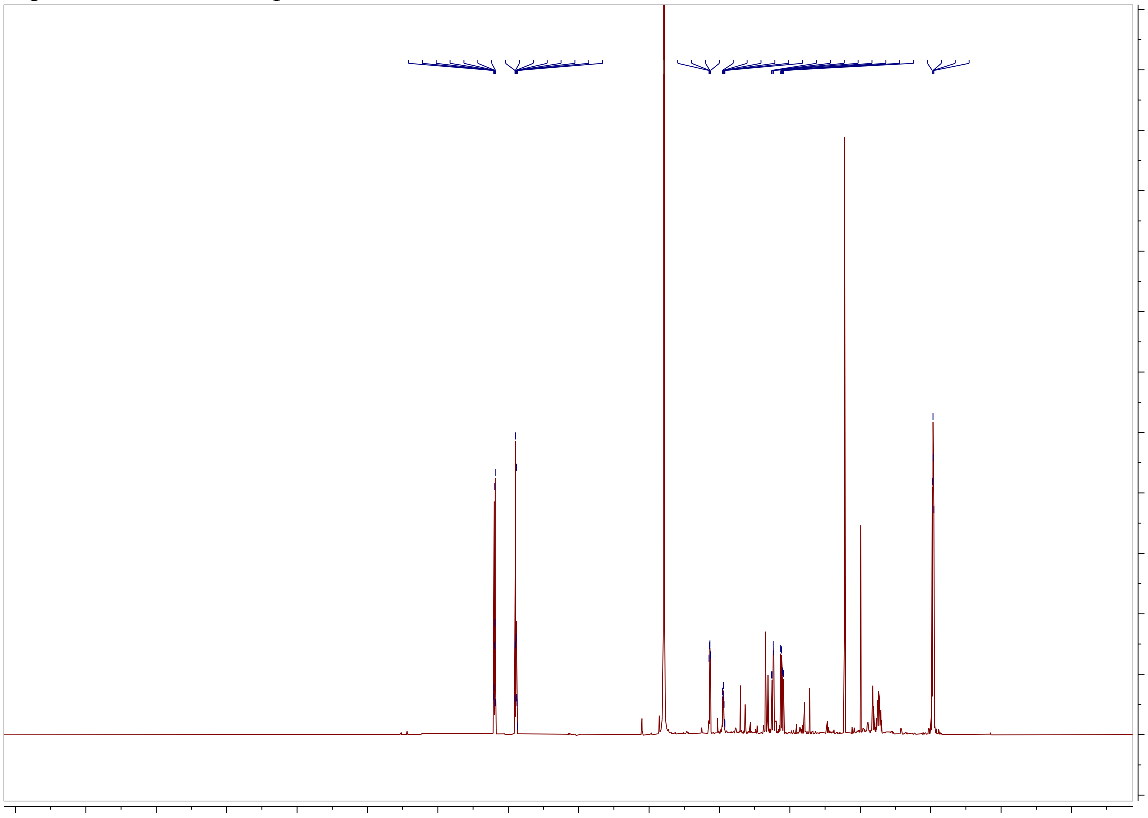

Figure S25. 1H-NMR spectrum of **17** (water-*d*₂, 289 K, 600 MHz).

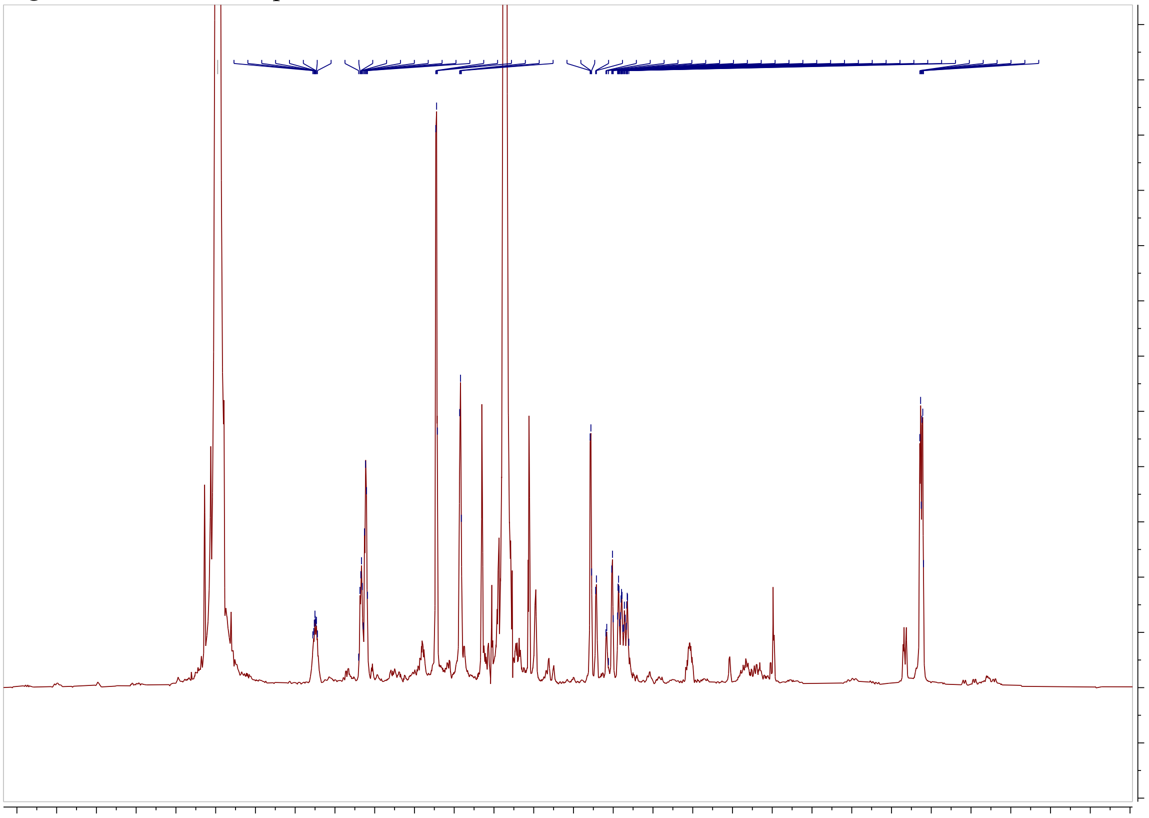

Figure S26. 1H-NMR spectrum of **18** (water-*d*₂, 289 K, 600 MHz).

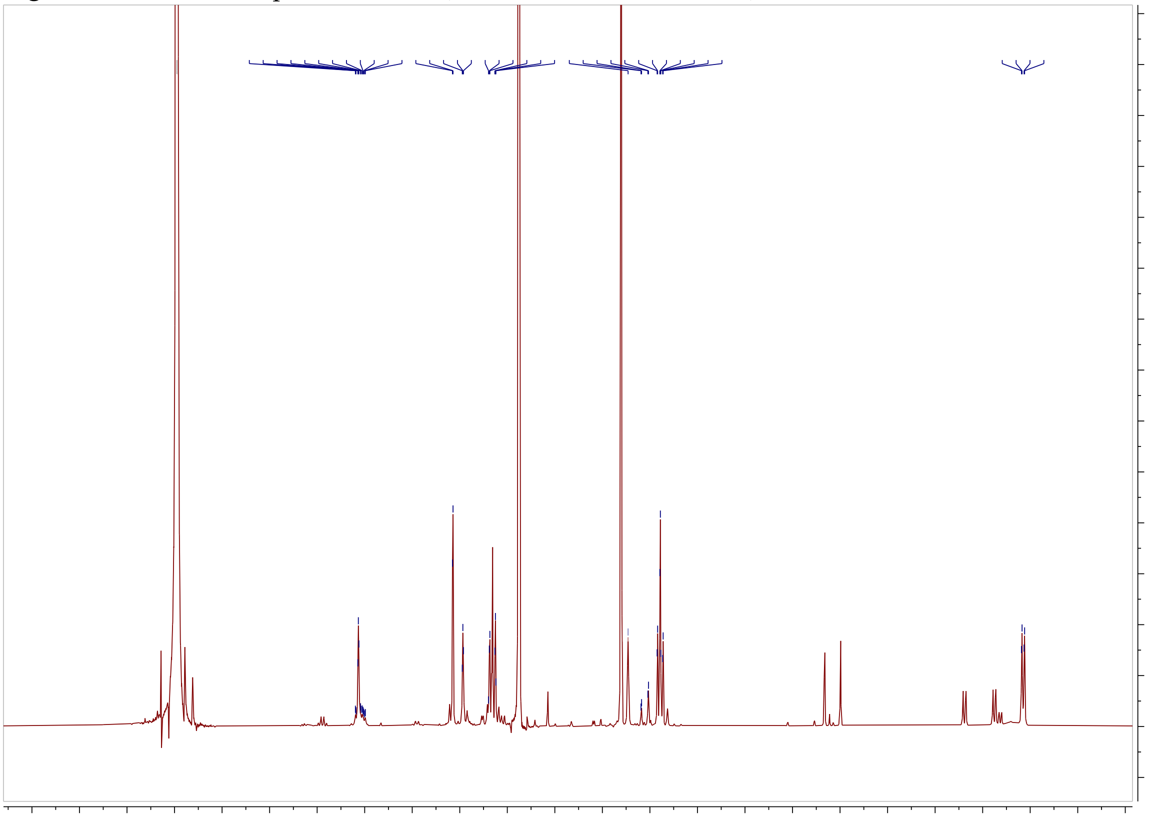

Figure S27. 1H-NMR spectrum of **19** (water-*d*$_2$, 289 K, 600 MHz).

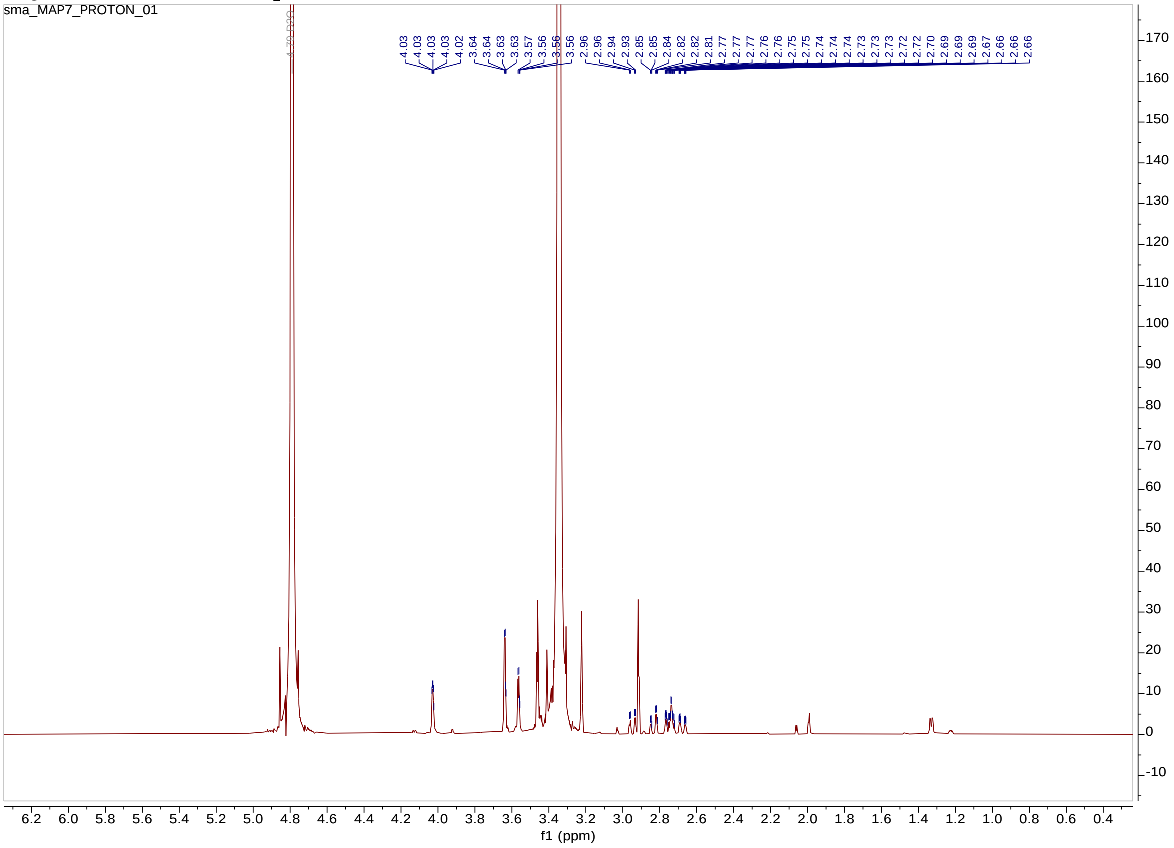

Figure S28. 1H-NMR spectrum of **20** (methanol-*d*$_4$, 289 K, 600 MHz).

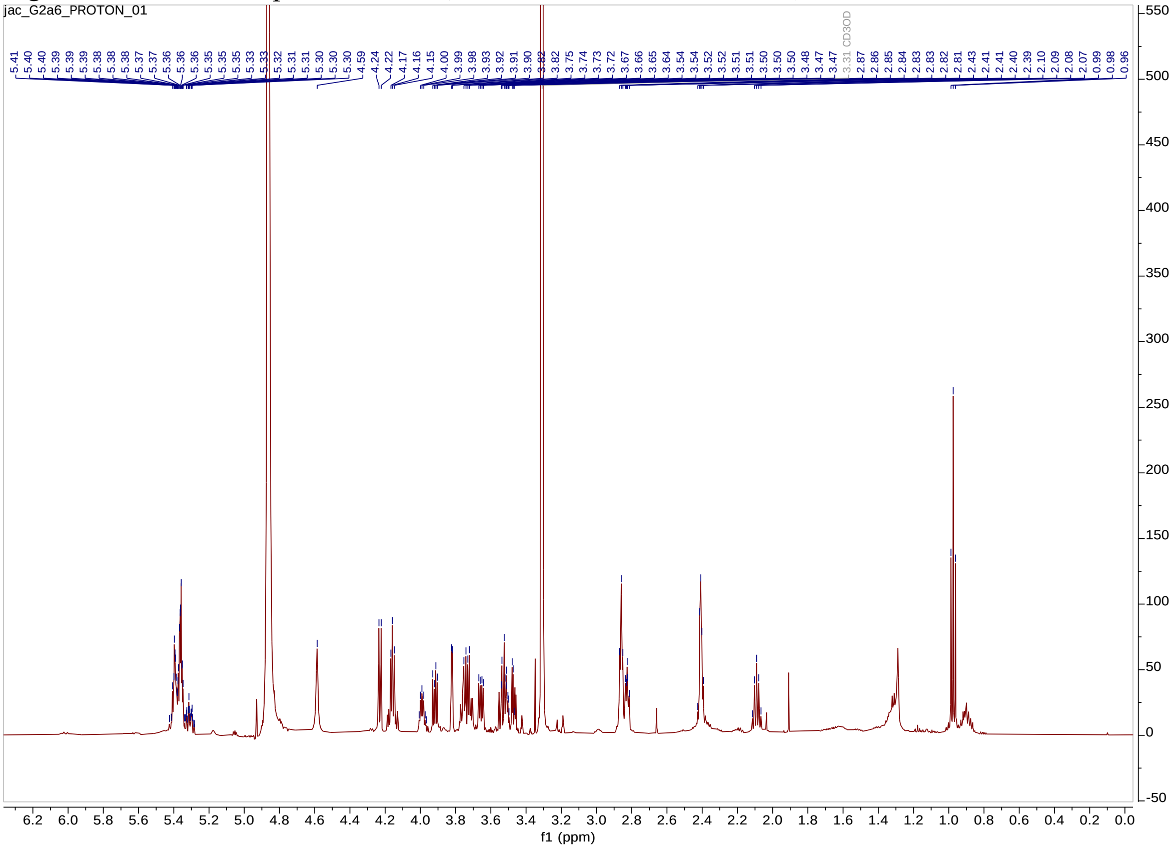

Figure S29. 1H-NMR spectrum of **21** (methanol-d_4, 289 K, 600 MHz).

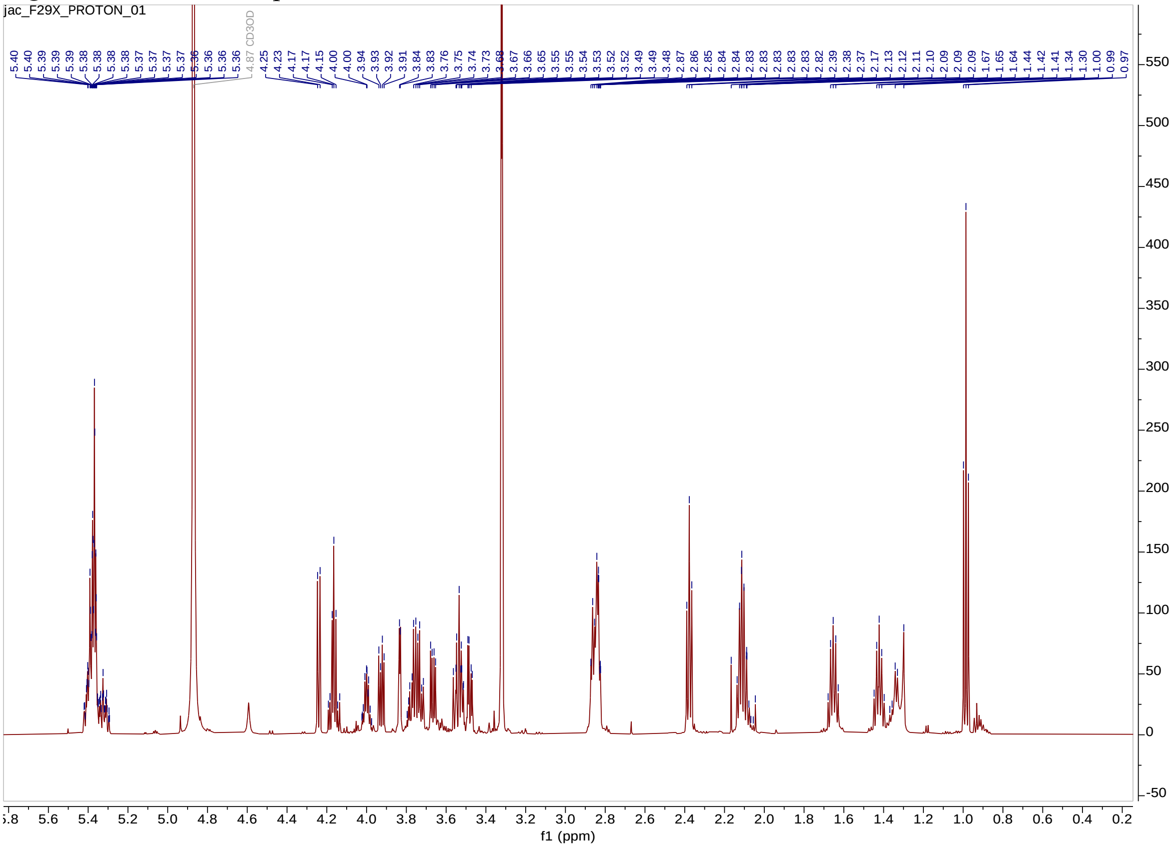

Figure S30. 1H-NMR spectrum of **22** (chloroform-d_1, 289 K, 600 MHz).

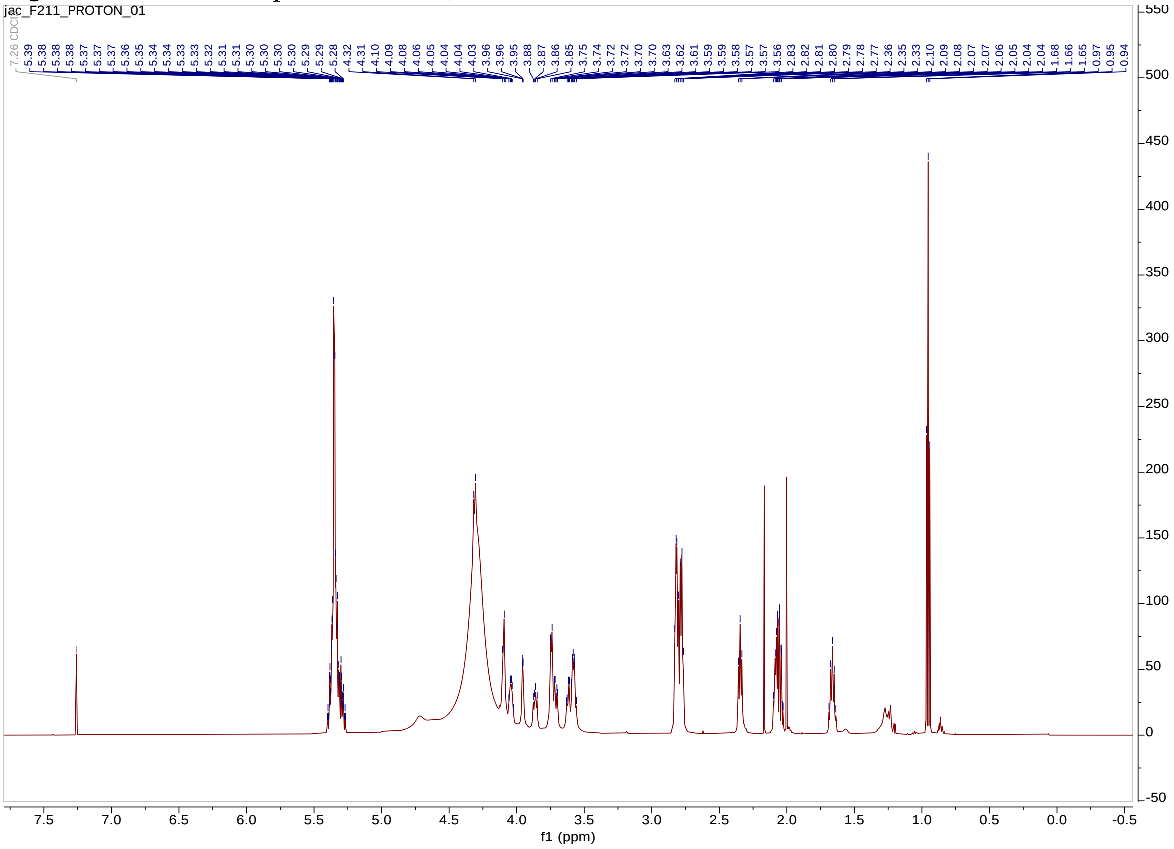

Supplement: Supplementary file 1 [file marinedrugs-20-00420-s001.zip › marinedrugs-1778309-supplementary.pdf]
